# Peptidomimetic α,β-Unsaturated Ethyl Esters Are Irreversible Inactivators of Human Cathepsin L and Are Potent Inhibitors of SARS-CoV‑2 in Cellular Models of COVID-19

**DOI:** 10.1021/acs.jmedchem.5c03172

**Published:** 2026-04-21

**Authors:** Vivek Kumar, Aleksandra Drelich, Bala C. Chenna, Andrew Rademacher, Alexandria M. Kemp, Anthony C. Bishop, Panatda Saenkham-Huntsinger, Elena Scott, A. Joshua Wand, Arthur Laganowsky, Chien-Te Tseng, Thomas D. Meek

**Affiliations:** † Department of Biochemistry and Biophysics, 14736 Texas A&M University, 301 Old Main Drive, College Station, Texas 77843, United States; ‡ Department of Chemistry, Texas A&M University, 580 Ross St. College Station, Texas 77843-3255, United States; § Department of Microbiology & Immunology Centers for Biodefense and Emerging Diseases, 12338 The University of Texas Medical Branch at Galveston, 301 University Boulevard, Galveston, Texas 77555, United States

## Abstract

Human cathepsin L (hCatL), a lysosomal cysteine protease, facilitates endosomal entry for SARS-CoV-2, making it a critical therapeutic target for COVID-19. Here, we report irreversible tripeptidomimetic inactivators containing a 3-pyridyl alanine residue at the **P**_**1**_ position, modified with either electrophilic α,β-unsaturated ethyl ester or sulfone warheads. These inhibitors had *K*_i_* values between 0.4 and 35 nM. The most potent inhibitors, **VK-168** (*K*_i_* = 0.58 nM) and **VK-261** (*K*_i_* = 0.38 nM), demonstrated robust anti-SARS-CoV-2 activity in Vero E6 (EC_50_ = 120–310 nM) and A549/ACE2 cells (EC_50_ = 78–120 nM). Additionally, **VK-80** inhibited infection (EC_50_ < 156 nM) and showed partial efficacy in a murine COVID-19 model. Native mass spectrometry and dilution assays confirmed irreversible hCatL inactivation. Notably, these enoates acted as reversible inhibitors of the homologue cathepsin B (hCatB). Molecular modeling suggests that hCatL’s active-site cysteine is better positioned for nucleophilic addition to the vinyl group than hCatB’s.

## Graphical abstract

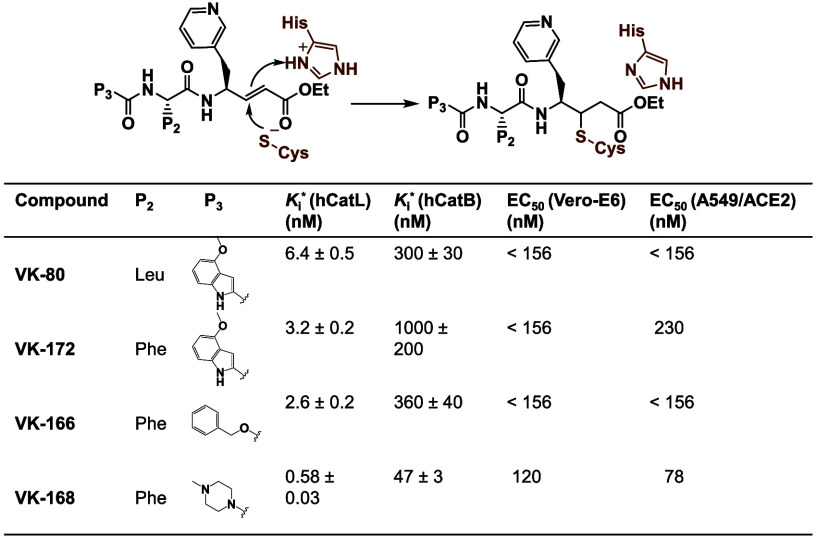

## Introduction

As of January 2025, the COVID-19 pandemic has resulted in 777 million cases and more than 7 million deaths worldwide.^1^ The FDA-approved drug Paxlovid, which was introduced for emergency use in December 2021, has successfully reduced the risk of hospitalization and death.^2,3^ Nirmatrelvir, the active component of Paxlovid, is a reversible covalent inhibitor of SARS-CoV-2 main protease (3CL-PR) in which a nitrile substituent forms a thioimidate adduct with active-site Cys_125_.^3–5^ The arrival of new strains of SARS-CoV-2 has led to diminished efficacy of Paxlovid, due to mutations appearing in 3CL-PR.^4,5^ Multiple mutations (E166V, F140L, S144A, L167F, A193P, among others) within 3CL-PR resulted in the most pronounced resistance to Nirmatrelvir.^4,5^ In addition, Nirmatrelvir is effective only in the presence of Ritonavir (the second active component of Paxlovid), which prevents the oxidation of Nirmatrelvir by human cytochrome 3A4 (CYP3A4).^6^ Accordingly, the long-term effectiveness of Paxlovid as a drug for COVID-19 is precarious, and a second generation of therapeutic agents for COVID-19 is needed.

Human cathepsin L (hCatL) is a ubiquitous endosomal cysteine protease (CP), which also is found in the cytoplasm and even outside of cells.^7^ It is associated with inflammation, certain cancers, and the cellular uptake of viruses, including SARS-CoV-1, Hendra, Nipah, Ebola, and Reoviruses.^8–12^

We demonstrated that K11777 (SLV-213), an irreversible covalent inactivator of cathepsins B, L, and S, was an exceptionally potent inhibitor (EC_50_ < 80 nM) of SARS-CoV-2 infection in Vero E6 and A549/ACE2 cells.^13^ In this study, an analogue of K11777 was used to covalently label, extract, and identify its targets as cathepsins B and L in the SARS-CoV-2-infected cells. While the actions of other cysteine proteases, such as cathepsins B and S, have been associated with the invasion of host cells by SARS-CoV-2,^14^ Ebola virus,^15^ and reoviruses,^16^ we focused on cathepsin L as a result of our earlier study^13^ because we showed that cathepsin L, but not cathepsin B, catalyzed cleavage of the SARS-CoV-2 Spike protein. We also recently demonstrated that inhibitors or inactivators of human cathepsin L are potent inhibitors of SARS-CoV-2 infection of permissive cell lines such as Vero E6 and A549/ACE2 cells.^13,17,23^ Endosomal viral entry into host cells depends upon cleavage of the SARS-CoV-2 Spike protein by cathepsin L, a crucial step in the disassembly of the virus inside of host cells.^13,18^ CatL is an important host cell factor for infection by SARS-CoV-2, in some but not all SARS-CoV-2 permissive cells.^7,18^ In addition, expression of hCatL has been shown to be upregulated in lung tissue collected from autopsy samples of COVID-19 patients,^7,18^ and the level of upregulation correlates with the extent of lung damage.^18^ When the circulating levels of hCatL were monitored in SARS-CoV-2-infected patients, the amount of hCatL expression was observed to be in correlation with the severity of COVID-19 infection.^18^ SARS-CoV-2 infection elevates the expression of hCatL, and overexpressed hCatL in turn accelerates the viral infection, forming a vicious cycle.^18^ Accordingly, hCatL has roles in both the initiation of SARS-CoV-2 infection, as well as in pulmonary pathology.^7,18^

The P_2_ and P_1_ peptide side chains most frequently found in substrates of cathepsin L include P_2_ = Phe, Leu, Trp, and Tyr, P_1_ = Lys, Arg, Gln, Leu, Phe, and no apparent selectivity at P_1′_.^19,20,23,24^ For 3CL-PR, amino acids found in these positions are P_2_ = Leu, Val, Phe, Met, P_1_ = Gln, His, and P_1′_ = Gly, Ala, and Ser (Figure 1A).^27–30,35^

**Figure 1:**
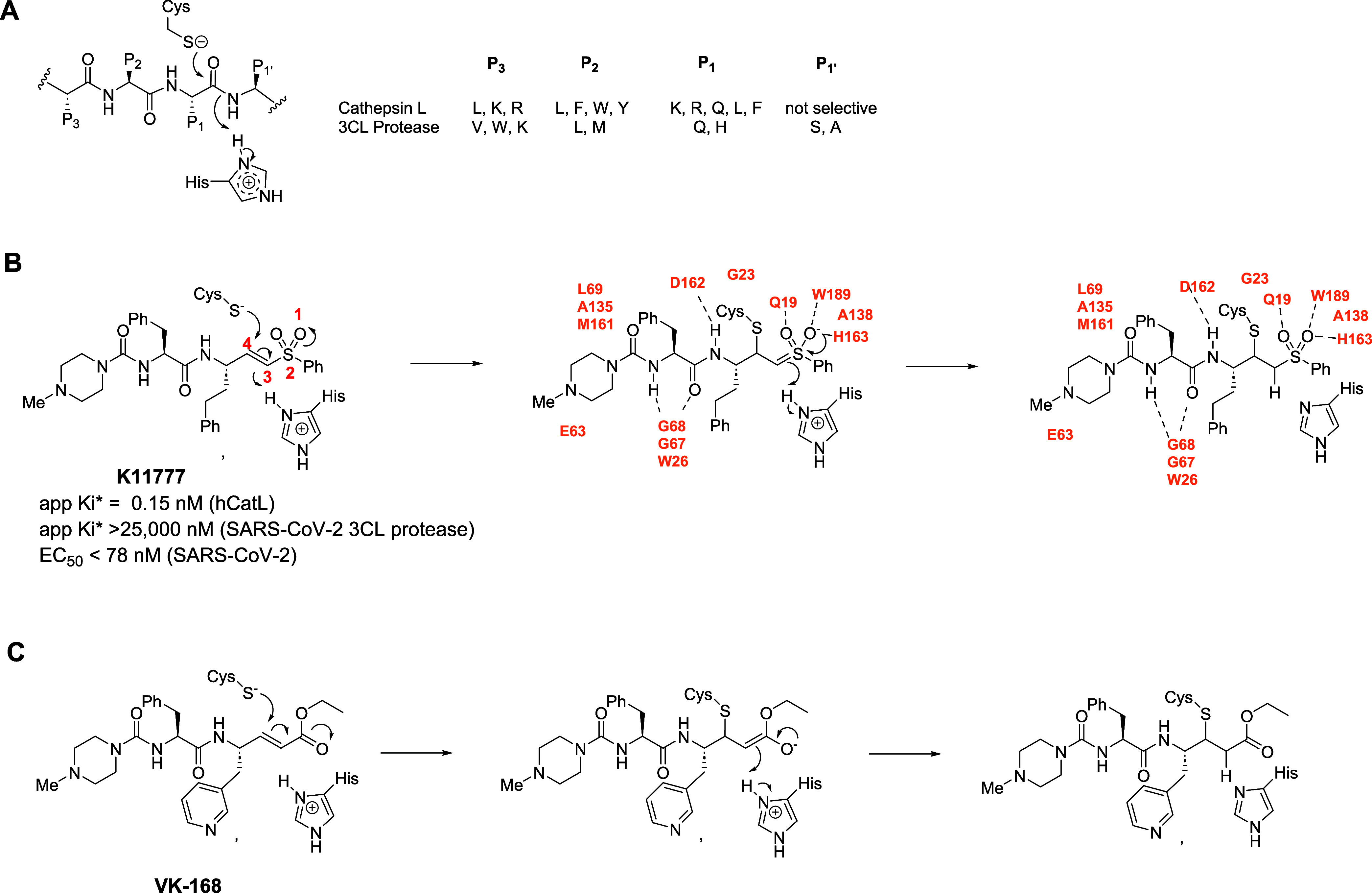
(**A**) Amino acid side chains typically found in native peptide substrates of hCatL and 3CL protease. Chemical mechanism of thia-Michael addition of active-site cysteine of hCatL to **K11777** (**B**) and the putative mechanism for this reaction with enoate **VK-168** (**C**). The apparent tight-binding inhibition constants (***K***_i_*) for hCatL and 3CL protease and the anti-SARS-CoV-2 activity (EC_50_) are included. The 1,4-thiol conjugate addition (red numbers) of the thiolate of Cys_25_ to the β-carbon of the vinyl sulfone or enoate warhead results in respective sulfonyl or enolate oxyanion intermediates, which are stabilized by hydrogen-bonding with residues in the S_1′_ subsite of hCatL. Other interactions between **K11777** and active-site residues (H-bonds are shown as dashes) are shown in orange.^36^

While the substrate specificities of 3CL-PR and cathepsin L^23,30–33,35^ differ in their preference for the P_1_ side chain, wherein the former protease has almost exclusive specificity for Gln while the latter enzyme is nonspecific, both proteases have high specificity for Leu at the P_2_ position (Figure 1A).^20,23,24,35^ We recently reported a series of peptidomimetic aldehyde inhibitors that inhibit both cathepsin L and 3CL-PR, in which we evaluated novel P_1_ side chains, namely, 2-pyridon-3-yl-alanyl, 3-pyridinyl-alanyl, and 1,3-oxazo-4-yl-alanyl groups designed to be chimeras of both Gln and Phe in order to be accommodated by both enzymes.^23^ The 3-pyridinyl-alanyl-containing inhibitors, while very potent vs hCatL, were considerably less potent vs 3CL-PR.

In this paper, we explore the use of 3-Pyr-Ala and 5-Ox-Ala as P_1_ amino acids in peptidomimetic scaffolds that contain two electrophilic warheads, α,β-unsaturated ethyl esters and α,β-unsaturated sulfones such as in **K11777** (Figure 1B), The warheads of these potential irreversible covalent inactivators are expected to also undergo thia-Michael addition to the active-site cysteines of CatL and 3CL-PR via the putative chemical mechanisms shown in Figure 1B,C.^35,36^ The rate of thiol-based 1,4-conjugate addition with these electrophiles (α,β-unsaturated carbonyls or sulfones) is expected to be enhanced by the stabilization of the α-carbanion generated after thiolate addition to the β-carbon of the electrophile.^27,28,36^ Vinyl sulfone and enoate warheads comprise “soft” electrophiles, that is, they are generally unreactive to thiols such as glutathione,^21,22,27,38^ and require the thiolate form of an active-site Cys to induce addition to the β-carbon of the vinyl group, followed by proton transfer to the α-carbon from the imidazolium form of His.^27,28,36^ From a crystal structure of the cathepsin L with bound **K11777**,^36^ the oxygen atoms of the sulfonyl group of **K11777** undergo hydrogen bonding with the Q19 side chain amide and the N–H group of W189 (Figure 1B), while A138, G23, and H163 in the S_1′_ subsite^36^ comprise the oxyanion hole, also affording stabilization of the vinyl sulfone oxyanion intermediate.^36^ From the results of molecular docking, we speculate a similar inactivation mechanism for compounds containing α,β-unsaturated esters (enoates) at the P_1′_ position (Figure 1C), and this putative mechanism is investigated here.

## Results and Discussion

### Kinetic Characterization and Anti-SARS-CoV-2 Activity of Inhibitors with Michael Acceptors

We evaluated time courses of the interactions of the compounds shown in Table 1 with cathepsins L and B and 3CL-PR. The resulting kinetic parameters (Figures 2 and S1–S6) and antiviral activities of inhibitors (Figure S10) in two cell lines (Vero E6 and A549/ACE2) are summarized in Table 1. The peptidomimetic inhibitors or inactivators reported here are analogues of tripeptides in which we focused mainly on two Michael acceptors, or “warheads”; an α,β-unsaturated ethyl ester (enoate) (for example, compound **1**) or an α,β-unsaturated phenyl sulfone (vinyl sulfone, for example, compound **2**). Our initial peptidomimetic scaffold contained the side chains: 3-Pyr-Ala (P_1_), Leu (P_2_), and 4-methoxy-indo-2-yl (4OMe-Ind; P_3_). We employed this scaffold here based on our previous work in order to develop inhibitors that would target both hCatL and 3CL-PR, owing to the fact that the pyridinyl group in the P_1_ side chain is sufficiently similar to glutamine as to be accommodated by 3CL-PR, which has stringent specificity for glutamine at the P_1_ position.^23,30–32,35^ As we showed in that study, when an aldehyde is appended to this scaffold (compound **3**, **BC-787**), which, while a potent inhibitor of hCatL (*K*_i_* = 1.7 nM), was a poor inhibitor of 3CL-PR (6.2 μM).^23^ Nevertheless, we prepared compounds **VK-80** (**1**) and **VK-192** (**2**) in which the aldehyde of **BC-787** was replaced, respectively, with an α,β-unsaturated ethyl ester and an α,β-unsaturated phenyl sulfone. Enoate **VK-80** exhibited time-dependent inhibition of hCatL (Figure 2a) with apparent values of *K*_i_ = 53 nM (obtained at 0–3 min post initiation) and *K*_i_* = 6.4 nM (obtained at 27–30 min post reaction initiation), demonstrating an approximately 10-fold increase in potency within 30 min of exposure to enzyme (Figure 2a,b). We obtained values of *k*_obs_ from time courses in Figure 2a and replotted them vs [**VK-80**] using both eqs 9 and 10 (Figure 2c). The plot is slightly downward curvilinear, indicating a transition from a first-order to zeroth-order dependence on [**VK-80**], from which we obtained the best fit using eq 9, resulting in values of *k*_inact_ = (2.1 ± 0.5) × 10^–2^ s^–1^, *K*_I_ = 320 ± 90 nM, *k*_4_ ∼ 0, and a calculated second-order rate constant of inactivation of *k*_inact_/*K*_I_ = (7 ± 2) × 10^4^ M^–1^ s^–1^ (Table S1). That *k*_4_ ∼ 0 indicated that **VK-80** is an inactivator of hCatL, and for hCatB, the value of *k*_inact_/*K*_I_ = (2 ± 1) × 10^3^ M^–1^ s^–1^ was 35-fold lower. In terms of apparent *K*_i_* values, **VK-80** is 50-fold more potent vs hCatL than its homologue hCatB. While **VK-80** was inactive against 3CL-PR, this compound was an exceptionally potent inhibitor of SARS-CoV-2 infection in both Vero and A549/ACE2 cells (EC_50_ < 156 nM, Figure S10 and Table 1), demonstrating that this anticoronaviral activity is due to its action on cathepsin L in these host cells.

**Figure 2:**
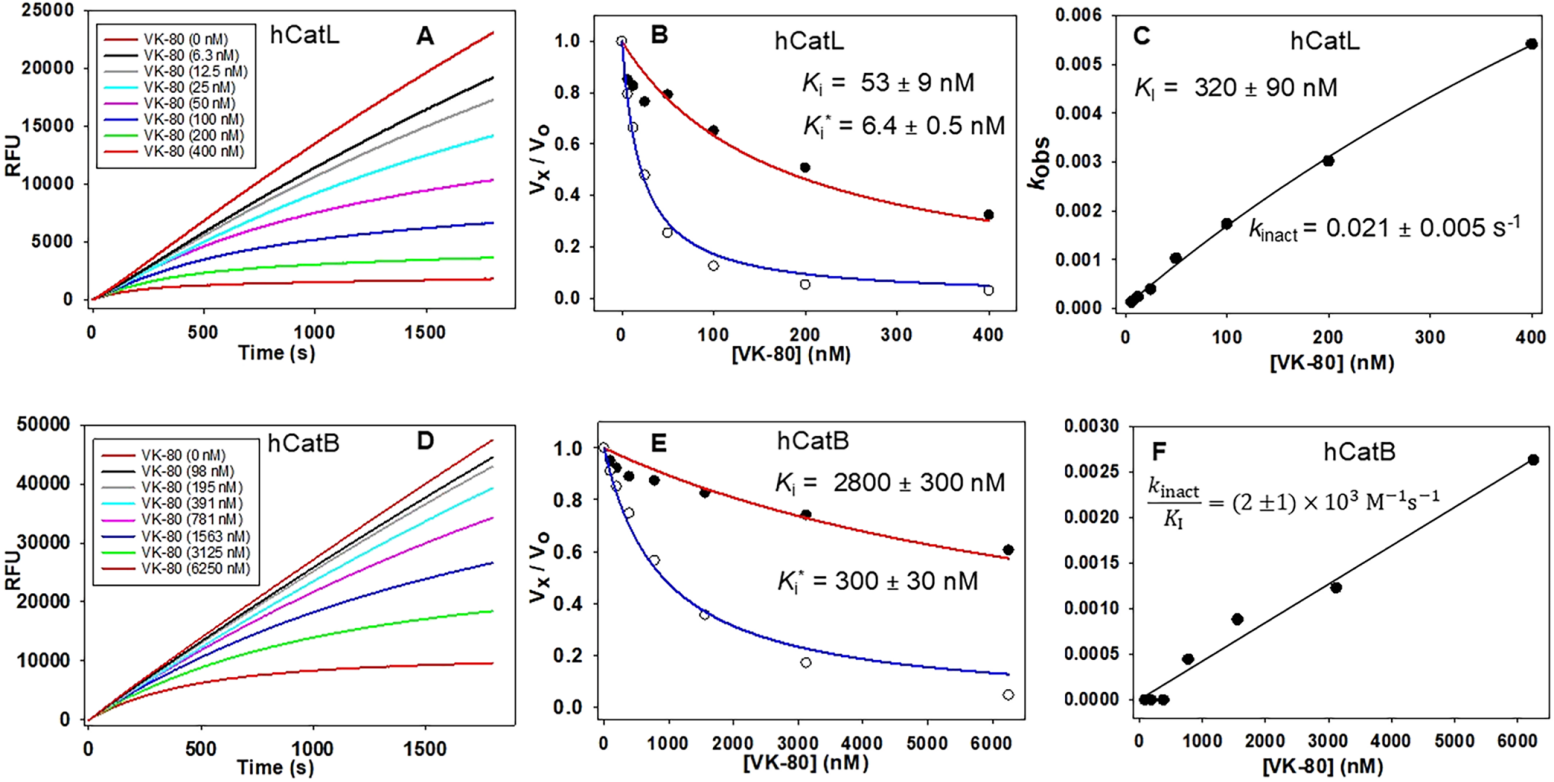
Inhibition of hCatL and hCatB by compound **1** (VK-80). Inhibition by compound **1** was observed in the time courses of peptidolysis reactions of hCatL (**A**; 0–400 nM) and hCatB (**D**; 0–6250 nM). Data from the time courses were fitted to the progress curve in eq 8, and *k*_obs_ values were determined at variable inhibitor concentrations [I]. *k*_obs_ was plotted vs [I] using eq 9 for hCatL and eq 10 for hCatB and second-order rate constants of inactivation (*k*_inact_/*K*_I_) for inhibitor **1** were determined for hCatL (**C**) and hCatB (**F**). Plotting of the fraction of remaining enzyme activity (*v*_*x*_/*v*_0_) vs inhibitor concentrations at initial rates (*t* = 0–180 s, (*v*_i_/*v*_0_)) and steady-state rates (*t* = 1620–1800 s, (*v*_s_/*v*_0_)) for hCatL (**B**) and hCatB (**E**). The lines drawn through the experimental data points resulted from fitting of data to eqs 5 and 6, from which the inhibition constants were obtained.

**Table 1:**
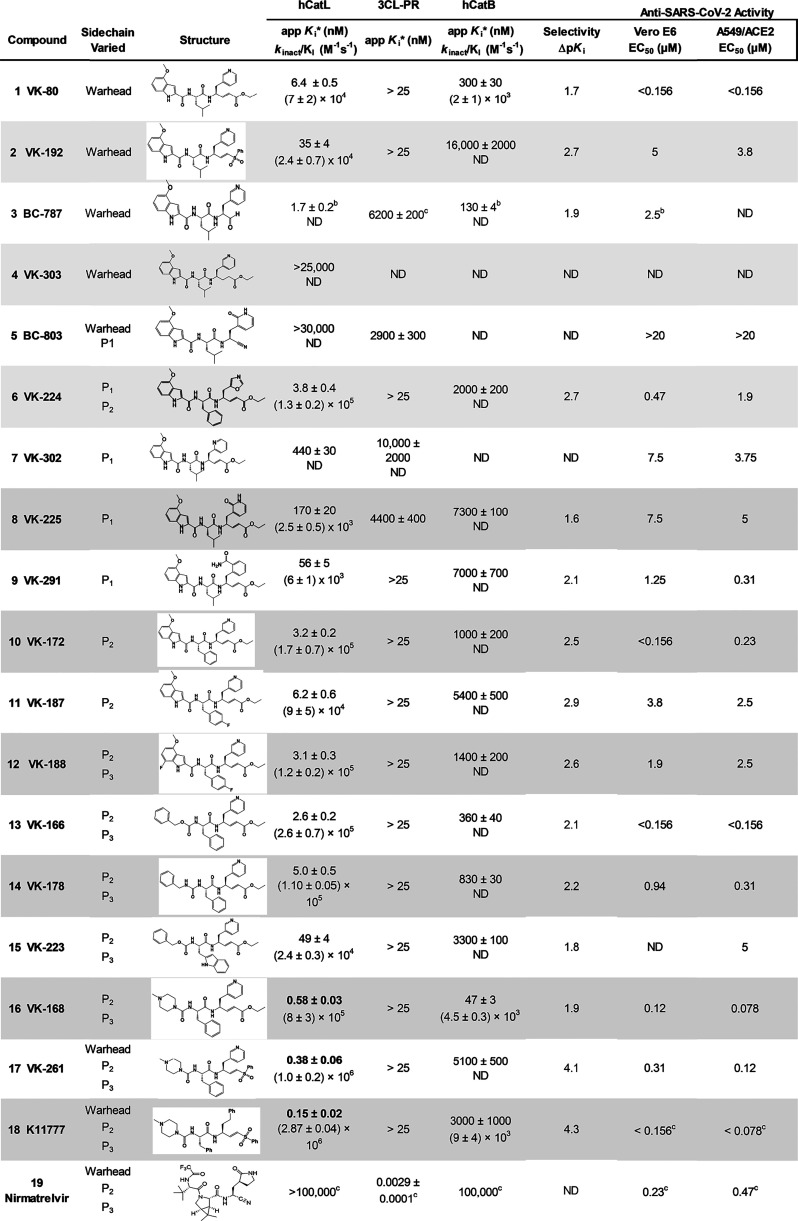
Kinetic Parameters and Anti-SARS-CoV-2 Activities of Inactivators of Human Cathepsins L and B and SARS-CoV-2 3CL-Protease^a^

a Inhibition/inactivation studies were performed at 25 °C and at pH 5.5 for human cathepsins L and B and pH 7.5 for SARS-CoV-2 3CL protease, respectively. The apparent tight-binding inhibition constants (*K*_i_*) were obtained at *t* = 27–30 min. *K*_i_* values in bold represent apparent steady-state inhibition constants calculated using eq 7, which includes the concentration of the enzyme since *E*_t_ ∼ *I*_t_. For vinyl esters and vinyl sulfones, time courses were fitted to eq 8 and values of *k*_obs_ were determined for each concentration of inhibitor [I]. Using the *k*_obs_ vs [I] plots, second-order rate constants of inactivation (*k*_inact_/*K*_I_) were calculated upon fitting to eqs 9 and 10. CP-100356 (at 2 μM), a pgp inhibitor, was included in the Vero E6 samples. Data for **K11777**, **BC-787**, and Nirmatrelvir were reported in refs 20, 23, and 30.

We prepared **VK-303** (**4**), the “saturated” analogue of enoate **VK-80**. Its lack of activity vs all three proteases demonstrated the importance of the α,β-unsaturated ethyl ester warhead in this class of inhibitors. Substitution of the α,β-unsaturated ethyl ester with a vinyl-sulfonylbenzene (**VK-192**) resulted in a time-dependent inhibitor that, like **VK-80**, became 10-fold more potent over time (*K*_i_* = 35 nM), albeit with 5-fold less potency than its enoate analogue (Table 1, Figure 2). This is surprising considering the exceptionally potent inactivation of hCatL by the vinyl sulfone moiety of **K11777** (*K*_i_* = 0.15 nM, *k*_inact_/*K*_I_ = 2.87 × 10^6^ M^–1^ s^–1^, Table 1) which forms a Michael adduct with Cys_25_ of cruzain, a close homologue of cathepsin L.^40^ Neither **VK-192** nor **K11777** inhibited 3CL-PR, and were 500- to 20,000-fold selective over hCatB, respectively. The modest anticoronaviral activities of **VK-192** (EC_50_ = 4–5 μM), again, arising solely from inhibition of cathepsin L, were commensurately less potent than those observed for **K11777** (EC_50_ < 0.156 μM). When the vinyl sulfone warhead of **VK-192** was replaced with a nitrile, the warhead found in Nirmatrelvir, the resulting compound **BC-803** (**5**, which contains the modest modification of a 2-pyridon-3-alanyl group at the P_1_ side chain) was inactive vs hCatL, and was a weaker inhibitor of 3CL-PR (***K***_**i**_ = 2.9 μM) than that of its aldehyde homologue, **BC-787**. Our findings that the enoate, vinyl sulfone, and nitrile warheads within the 4OMe-Ind-Leu-3-Pyr-Ala scaffold provided poor inhibitors of 3CL-PR led us to systematically replace the P_1_–P_3_ residues in new peptidomimetic scaffolds that contained enoate or vinyl sulfone warheads (Table 1).

For compounds **6–9**, the 3-Pyr-Ala P_1_ side chain of enoate **1** was replaced, respectively, by 1,3-oxazo-5-alanyl (5-Oxa-Ala), pyridin-2-alanyl, 2-pyridon-3-alanyl, and *o*-carbamoyl-Phe side chains. When appended to an aldehyde warhead, the P_1_ 1,3-oxazo-4-alanyl and 2-pyridon-3-alanyl groups were shown to be excellent isosteres of glutamine as well as very potent inhibitors of both hCatL and 3CL-PR due to covalent adduction to the active-site cysteines of either protease.^23^ The enoate **6** (**VK-224**), containing the P_1_ 1,3-oxazo-5-alanyl group, exhibited potent inhibition of hCatL (*K*_i_* = 3.4 nM, *k*_inact_/*K*_I_ = 1.4 × 10^5^ M^–1^ s^–1^, Table 1, Figures S3 and S6), but surprisingly, was inactive vs 3CL-PR. Nevertheless, it effected anti-SARS-CoV-2 activity in both cell lines at low micromolar concentrations. Enoate **7** (**VK-302**), containing the pyridin-2-alanyl group at P_1_, was a poor inhibitor of hCatL (*K*_i_* = 400 nM) and 3CL-PR (*K*_i_* = 10,000 nM) (Figure S4). **VK-225** contains a 2-pyridon-3-yl group at P_1_, which despite the potency of the aldehyde in this scaffold (**BC-787**), proved to be, as an enoate, a poor inhibitor of both hCatL (*K*_i_* = 170 nM) and 3CL-PR (***K***_**i**_ * = 4.4 μM), with moderate anti-SARS-CoV-2 activity in both cell lines at low micromolar concentrations (Figures S3, S5, and S6). The modified 3-carbamoyl-Phe group at the P_1_ position of **VK-291** resulted in a modest inhibitor of hCatL (*K*_i_* = 56 nM, Figure S3), which was also inactive vs 3CL-PR.

Substitution of the Leu P_2_ group in **VK-80** by a Phe (**VK-172**) and a *p*-fluoro Phe (**VK-187**) provided excellent inactivators of hCatL, (*K*_i_* = 3.2 nM, *k*_inact_/*K*_I_ = 1.7 × 10^5^ M^–1^ s^–1^, Figures S1 and S6) and (*K*_i_* = 6.2 nM, *k*_inact_/*K*_I_ = 9 × 10^4^ M^–1^ s^–1^ Figure S2, respectively), indicating that the P_2_ Leu and Phe groups are interchangeable as hCatL inhibitors. As with **VK-80**, neither **VK-172** nor **VK-187** inhibited 3CL-PR. However, both compounds inhibited cellular infection by SARS-CoV-2 (EC_50_ < 0.156, 0.32, and 2.5–3.8 μM, respectively) due to hCatL inhibition, for which the substitution of fluorine on the Phe side chain lowered the anticoronaviral activity by 10-fold. Addition of a fluorine atom to C-6 of indole **VK-187** afforded compound **12** (**VK-188**). Both compounds were equipotent vs the three cysteine proteases, and in cell infection assays (**VK-188**, EC_50_ = 1.9, 2.5 μM). Remarkably, **VK-187** and **VK-188** differ from **VK-172** only by the substitution of fluorine in the former compounds, and despite their equipotency vs hCatL, the fluorine-substituted compounds are ≥10-fold less active as anti-SARS-CoV-2 agents due to the addition of fluorine.

We next made compounds in which the P_3_ 4OMe-Ind group was substituted by Cbz (compound **13**, **VK-166**) or its urea analogue (compound **14**, **VK-178**). The latter compound was expected to be more metabolically stable than the former.^29^ The two compounds were nearly equipotent vs hCatL (*K*_i_* = 2.6 and 5.0 nM, respectively; Figure S1), neither inhibited 3CL-PR, but both were active in the two cell infection assays in which **VK-166** along with **VK-80** were highly effective anti-SARS-CoV-2 agents in our study (EC_50_ < 156 nM, Figure S10). Apparently, the urea linkage in **VK-178** impedes the ability of this compound to access the target hCatL in SARS-CoV-2-infected cells (EC_50_ = 310 and 940 nM, Figure S10). The substitution of Phe in **VK-166** with a Trp residue resulted in a poor inhibitor of hCatL (**VK-223**, *K*_i_ = 49 nM, Figure S3), which was inactive vs 3CL-PR, and poorly active in cell infection assays (EC_50_ = 5000 nM, Figure S10).

For **VK-168**, we replaced the P_3_ Cbz group with the 4-*N*-methyl-piperazinyl-carbonyl (NMePip) group found in **K11777**. The resulting enoate was an exceptionally potent inactivator of hCatL (*K*_i_* = 0.58 nM, *k*_inact_/*K*_I_ = 1.7 × 10^6^ M^–1^ s^–1^, Figures S1, S6), had no activity vs 3CL-PR, and commensurate with its potent inhibition of hCatL, was the most effective enoate in blocking SARS-CoV-2 infection (EC_50_ = 120 and 78 nM, Figure S10). Substitution of the ethyl enoate warhead of **VK-168** with the vinyl sulfone afforded compound **17** (**VK-261**), which has a very similar structure to **K11777** and VK-268, is only a 2-fold less potent inhibitor of hCatL than **K11777** (**VK-261**, *K*_i_* = 0.38 nM, *k*_inact_/*K*_I_ = 1.0 × 10^6^ M^–1^ s^–1^, Figures S2 and S5). Like **K11777**, **VK-261** had no activity vs 3CL-PR, so that its potent anti-SARS-CoV-2 activity (EC_50_ = 0.12–0.32 nM, Figure S10) was due to its inhibition of hCatL. It is noteworthy that this NMePip-Phe-3-PyrAla scaffold containing an enoate warhead was more than 2-fold more potent in the cell infection assays than the scaffold bearing a vinyl sulfone warhead and was also equipotent with the benchmark inactivator of hCatL, **K11777**. Also, the enoate and vinyl sulfone warheads on the NMePip-Phe-3-PyrAla scaffold provided inhibitors of nearly equal subnanomolar potencies, unlike the case of **VK-80** (enoate) and **VK-192** (vinyl sulfone).

For hCatL, values of *k*_inact_/*K*_I_ for the compounds in Table 1 ranged between (2.5 ± 0.5) × 10^3^ M^–1^ s^–1^ (**VK-225**) and (1.00 ± 0.2) × 10^6^ M^–1^ s^–1^ (**VK-261**), the latter of which approached the value for **K11777** (2.82 ± 0.04) × 10^6^ M^–1^ s^–1^ (Figures S3 and S6). For these fittings, values of *k*_4_ ranged from 0 to 2.0 × 10^–4^ s^–1^. However, in the control sample (no inhibitor) a value of *k*_4_ of 1.4 × 10^–4^ s^–1^ was obtained, indicating that these other values of *k*_4_ are not significantly different than the control value. This indicated that the compounds evaluated in this manner were kinetically irreversible inactivators of hCatL. For hCatB, we were only able to obtain values for *k*_inact_/*K*_I_ for **VK-80** ((2 ± 1) × 10^3^ M^–1^ s^–1^), **VK-168** ((4.5 ± 0.3) × 10^3^ M^–1^ s^–1^), and **K11777** ((9 ± 4) × 10^3^ M^–1^ s^–1^), and these parameters were 35-fold to 1,000-fold lower for hCatB compared to hCatL.

The rate constants of inactivation for compounds in Table 1 were in a limited range, *k*_inact_ = 0.009–0.056 s^–1^, in which the lowest value was of the enoate **VK-223**, which contains a Trp group as the P_2_ group, while the largest value was for **VK-188**, which contains a 4-fluoro-Phe group in this side chain. The Trp side chain may not be well accommodated in the S_2_ site (app *K*_i_ = 280 nM), which possibly retards its ability to react with the active-site cysteine. The largest value of *k*_inact_ = 380 s^–1^ was obtained for aldehyde **BC-787**, which is consistent with its rapid addition to Cys_25_ in the formation of the thiohemiacetal.

We conclude from this that the peptidomimetic scaffold affects the reactivities of the enoate and vinyl sulfone warheads in terms of covalent addition to Cys_25_ of hCatL, and that the 3-Pyr-Ala and 5-Oxa-Ala P_1_ groups apparently do not interfere with this adduction chemistry, such that a homo-Phe group as found in **K11777** is not required to overcome any steric hindrance.

### Interaction of Nirmatrelvir and **VK-168** in SARS-CoV-2-Infective Cells

Independent evaluation in SARS-CoV-2-infected Vero E6 and A549/ACE2cells of variable concentrations of nirmatrelvir and **VK-168** resulted in respective values of EC_50_ = 120 and 78 nM (Figure S10). In another sample containing the same variable concentrations of **VK-168** and including 25 nM nirmatrelvir in each sample, the EC_50_ value for **VK-168** was reduced by 2-fold (60 nM in both cell lines, Figure S10). This indicated an additive interaction between the two compounds.

### Preincubation Studies

We utilized preincubation data to determine the reversibility of our inhibitors with hCaL and hCatB, from which we expected to ascertain reliable values of *k*_4_. Here, 500 nM of each inhibitor was preincubated with 100 nM hCatL for 1 and 12 h each, followed by 100-fold dilution into reaction mixtures containing 20 μM Cbz-Leu-Arg-AMC (5*K*_m_). For hCatB, the preincubation solutions were comprised of 250 nM hCatB and 2500 nM inhibitors, and after a 12 h period of preincubation, these were diluted 100-fold into reaction mixtures containing 50 μM Cbz-Leu-Arg-AMC. Control samples contained DMSO. The resulting time courses are found in Figure 3 for both preincubation times for hCatL. Other data showing time courses of recovery of enzyme activity and comparisons of time courses are found in Figures S7–S9.

**Figure 3:**
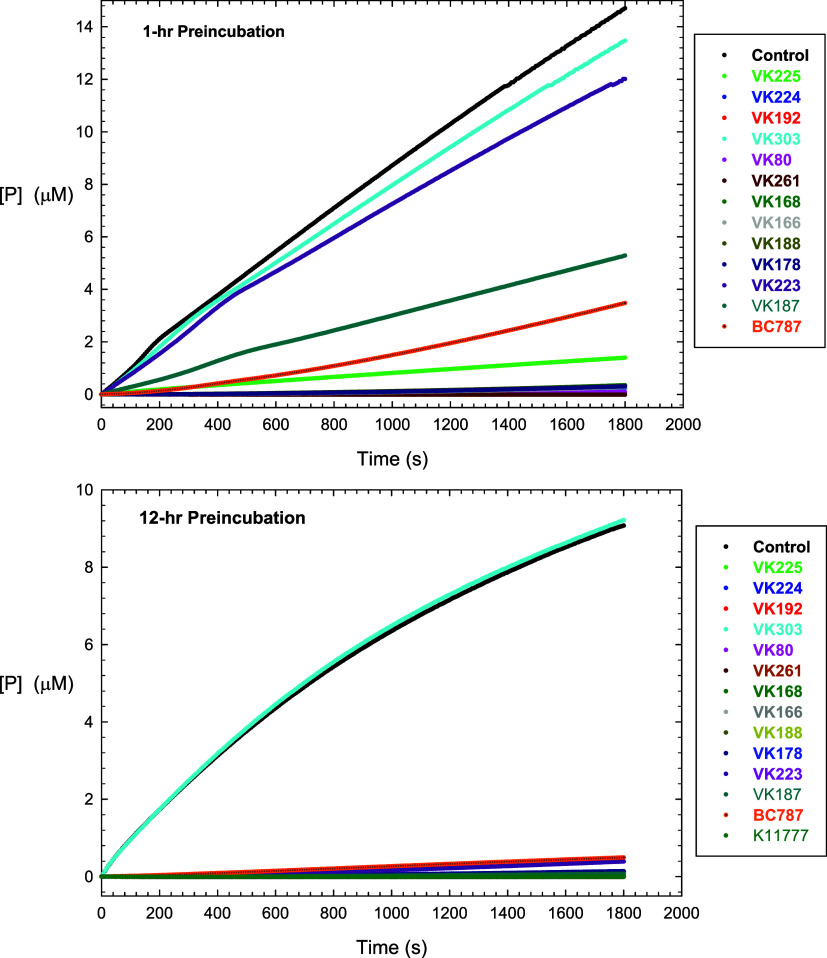
Time courses of residual enzyme activity of cathepsin L (100 nM) in preincubation mixture with 500 nM of each inhibitor for 1 h (**top**) or 12 h (**bottom**), followed by 100-fold dilution into reaction mixtures containing 20 μM Cbz-Leu-Arg-AMC. Control (DMSO, black), **VK-225** (light green), **VK-224** (blue), **VK-192** (red), **VK-303**, (cyan), **VK-80** (magenta), **VK-261** (dark red), **VK-168** (dark green), **VK-166** (gray), **VK-188** (dark yellow), **VK-178** (dark magenta), **VK-187** (dark cyan), and **BC-787** (orange).

Data fitting is found in Table S2. Residual hCatL activities following preincubation at both time points with **VK-303** were nearly superimposable with that of the control sample, as expected for a compound that is not an inhibitor of hCatL. However, the values of *v*_s_/*E*_t_ = 7.4–8.07 s^–1^ obtained at 1 h preincubation were larger than the samples after 12 h of preincubation (*v*_s_/*E*_t_ = 4.8–4.9 s^–1^), indicating >40% loss of hCatL activity following the lengthy preincubation period. For the peptidomimetic aldehyde **BC-787**, a recovery of hCatL activity was observed after 400 s for the 1 h preincubation sample (*k*_4_ = 2.3 × 10^–3^ s^–1^, *v*_s_/*E*_t_ = 2.2 s^–1^; eq 11), while recovery of enzymatic activity was less for the 12 h sample (*k*_4_ = 1.1 × 10^–3^ s^–1^, *v*_s_/*E*_t_ = 0.29 s^–1^), indicating that for this aldehyde, equilibrium between **EI** and EI* was not achieved in an hour. This demonstrated the slow reversibility of thiohemiacetal formation with hCatL. Fitting of the time courses after 1 h preincubation for **VK-80**, **VK-261**, **VK-166**, **VK-168**, and **K11777** to eq 11 provided no values of *v*_s_ or *k*_4_, indicating that these compounds effected irreversible inactivation of hCatL, as has been previously demonstrated for **K11777**. No restoration of activity was observed for these compounds in the 12-h preincubation samples. This irreversible inactivation is undoubtedly due to the covalent adduction of these compounds with active-site Cys_25_.

Enzyme treated with the vinyl sulfone **VK-192** had residual activity after 1 h of preincubation (*k*_4_ = 2.07 × 10^–3^ s^–1^, *v*_s_/*E*_t_ = 0.038 s^–1^, 1% activity), but no activity following 12 h of preincubation. This demonstrated that the enoate (**VK-60**) achieved inactivation of hCatL much faster than its vinyl sulfone analogue **VK-192**. Compounds **VK-225**, **VK-187**, **VK-188**, **VK-178**, **VK-223**, and **VK-224** all also showed significant residual activity (*v*_s_/*E*_t_ = 0.35–6.2 s^–1^, 5–86% residual activity) after the 1 h preincubation. This is notable since time-dependent inhibition was observed for these compounds in which residual enzyme activity for each compound after 30 min was very low. In other words, the EI* complexes for these compounds were well established within 30 min, but irreversible inactivation (*k*_4_ approaches 0, *k*_5_ > 0) with these compounds was not evident until a preincubation period of 12 h, in which *v*_s_/*E*_t_ = 0 for **VK-225**, while the other compounds retained minimal activity (*v*_s_/*E*_t_ = 0.084–0.287 s^–1^, 2–6% residual activity), indicating incomplete inactivation, or partial recovery of activity. For **VK-166**, while activity of hCatL was negligible after 1 h (*k*_4_ = *v*_s_/*E*_t_ = 0 s^–1^) of preincubation, this complete inactivation was partially reversed after 12 h of preincubation (*k*_4_ = 13 s^–1^ and *v*_s_/*E*_t_ = 0.3 s^–1^, 6% residual activity). This suggested that the putative covalent adduct of **VK-166** to Cys_25_ is readily formed with **VK-166** but is slowly reversible over time.

We also performed preincubation studies (12 h) with hCatB (Figure S9). Results of these studies provided a different view of the selectivity of our compounds for hCatL vs hCatB. In all cases, residual activities of hCatB remained after 12 h of preincubation, for which values of *k*_4_ = 81–310 s^–1^ and *v*_s_/*E*_t_ = 0.87–2.52 s^–1^ (31–107% residual activity) were obtained. Despite the fact that our compounds were inhibitors, albeit poor ones, of hCatB (*K*_i_* = 47–16,000 nM), none of these inhibitors achieved inactivation with hCatB, while **VK-80**, **VK-192**, **VK-168**, **VK-225**, **VK-261**, and **VK-188** resulted in 0–2% residual activity of hCatL under the same reaction conditions. At early interaction times (*t* < 30 min) between the majority of our compounds and the two cathepsins, apparent inhibition was observed for both proteases, albeit with considerably weaker inhibition of hCatB. However, beyond 1 h of interaction, preincubation studies demonstrated that residual activities for hCatL were either low (1–35%) or negligible (0%), while hCatB in the same time scale had recovered 100% of its activity. Accordingly, hCatL inhibition or inactivation from these compounds will persist long after these inhibitors have been extruded from cells, while this will not be the case for hCatB. Our study provides an excellent example of the importance of drug-residence time on a primary target (in this case, hCatL), and how differences in residence times contribute to selectivity vs off-targets (hCatB).^43^

### Selectivity of Compounds between hCatL and hCatB

Cathepsins L and B are both lysosomal proteases that are found ubiquitously in human cells. While these cysteine proteases share 16% amino acid identity and pH optima, they have different substrate specificities.^19,20,37^ Inhibition of compounds against hCatB (Figures S1–S4) indicated that, unlike hCatL, the S_1_, S_2_, S_3_, and S_1′_ subsites of hCatB are more selective and sensitive to small or moderate changes at P_1_, P_2_, P_3_, and P_1′_ subgroups of inhibitors.^19,20,36^ This was reflected in the apparent *K*_i_* values of hCatB that were considerably larger than those of hCatL. Figure S11 shows plots of p*K*_i_ for compounds in Table 1 for both proteases, and the differences in these values, and the selectivity differences in these compounds are reported in Table 1. The lowest difference in selectivity between the two proteases was found for **VK-80**, **VK-223**, **VK-225**, and **VK-168**, in which these compounds were 50-fold to 81-fold less potent for hCatB than hCatL (Δp*K*_i_ = 1.8–2.7). The selectivity for other compounds ranged from 81-fold (**VK-168**) to 13,000-fold (**VK-261**), and for values of *k*_inact_/*K*_I_, **VK-80** and **VK-168** were 83-fold and 830-fold higher, respectively, for hCatL. In general, the vinyl sulfone-containing compounds showed greater selectivity for hCatL over hCatB, more than the corresponding enoates. The enoates **VK-224**, **VK-172**, **VK-187**, **VK-188**, **VK-178**, and **VK-166** all exhibited >100-fold selectivity for hCatL vs hCatB.

Given that the compounds described above have greater potencies vs hCatL than hCatB, we evaluated E-64 and CA-074 methyl ester, both epoxide-containing inactivators, in SARS-CoV-2-infected Vero E6 and A549/ACE2 cells. E-64, a general inactivator of cysteine proteases, is equipotent in terms of enzyme inactivation vs hCatL and hCatB (respective values of IC_50_ = 2.5 and 4.1 nM, Biotechne Tocris datasheet). CA-074 methyl ester is a cell-penetrant inactivator that, after metabolic removal of its ester, is a specific inactivator of hCatB (enzyme inactivation: IC_50_ = 2–5 nM, for hCatL (IC_50_ > 40 μM), Biotechne Tocris datasheet). E-64 had no effect at concentrations of ≤20 μM in either SARS-CoV-2-infected Vero E6 or A549/ACE2 cells. CA-074 methyl ester demonstrated modest anti-SARS-CoV-2 activity in infected Vero E6 (EC_50_ = 2.5 μM) and had no activity in A549/ACE2 cells (EC_50_ > 20 μM), in comparison to the hCatL-specific inactivator **K11777** (Vero E6 (EC_50_ = 0.156 μM) and A549/ACE2 cells (EC_50_ = 0.31 μM)) in the same set of studies. This indicated that the action of hCatB, particularly in Vero E6 cells, may contribute in a minor capacity to the viral infectivity of SARS-CoV-2. In terms of the side chains that contributed to specificity between the two proteases, for the P_3_ side chains, the 4OMe-Ind group displayed the highest selectivity (specificity ratio (SR): *K*_i_*_hCatB_/*K*_i_*_hCatL_ = 300; Figure S12), and selectivity decreased in the order of 4OMe-Ind > Cmz > Cbz > *N*-Me-Pip, with *N*-Me-Pip being the least selective with a specificity ratio (SR) equal to 80. Comparing the P_2_ amino acids in terms of selectivity, 4F-Phe displayed the highest selectivity (SR = 870), which decreased in the order 4F-Phe > Phe > Leu, with Leu being the least selective with an SR = 50. Hence, hCatB is more selective for Leu in the S_2_ subsite, and affinity decreased with the increasing size of the P_2_ side chain from Leu > Phe > 4F-Phe > Trp. In contrast, Phe appears to be optimal for the S_2_ subsite of hCatL, and replacing Phe with Trp resulted in a 10-fold decrease in the level of time-dependent inactivation. In terms of the P_1_ subgroups, 5-Ox-Ala and 3-Pyr displayed similar *K*_i_*_hCatL_ values, but 5-Ox-Ala was 2-fold more selective for hCatL vs hCatB. 2-Pyrd-Ala and 2-CB-Phe, on the other hand, displayed poor *K*_i_*s vs both proteases. Inhibition/inactivation by the vinyl ethyl ester and the vinyl phenyl sulfone indicated that the S_1′_ subsite of hCatB is highly selective compared to hCatL. Selectivity of the two most potent hCatL inhibitors, which differ structurally only in their P_1′_ covalent warheads, **VK-168** (*K*_i_* = 0.58 ± 0.03) and **VK-261** (*K*_i_* = 0.38 ± 0.06), exhibited 80-fold and 5250-fold specificity, respectively. Structural features of these selective enoates will inform the design of the next generation of inhibitors.

### Native Mass Spectrometric Evaluation of the Interaction of Inhibitors with hCatL

We employed native mass spectrometry to investigate the nature of the binding of **VK-80**, **VK-261**, and **BC-787** to hCatL (Figure 4). After buffer exchange of the enzyme–inhibitor complex into ammonium acetate, we observed a +33 Da adduct on the protein that increased as time progressed (Figure 4). This may be due to a change in hCatL conformation after buffer exchange along with the deprotonation of a few of the acidic amino acids and addition of ammonium ions that led to the shift of +33 Da over time. The molecular weight of apo hCatL was found to be 24 162 Da. Upon preincubating hCatL with a 2.5-molar excess of **VK-80** and **VK-261** for 1 h, we observed mass shifts of +508 Da and +563, respectively (Figure 4). These mass shifts matched the molecular weight of hCatL 1 plus the molecular weights of the respective inhibitors, and the inhibitors remained bound with an increased collision energy of 150.0 eV (Figure 4). These mass shifts indicated that a stable covalent complex had formed, suggesting that the mechanism of conjugate-1,4 thiolate addition followed by protonation of the α-carbon of the corresponding α,β-unsaturated ethyl ester in **VK-80** had occurred, as was the same for the α-carbon of the α,β-phenyl sulfone in **VK-261** (Figure 5). Increasing collision energy resulted in the same intensities of peaks in both spectra of inhibitors **VK-80** and **VK-261**, which indicated that the covalent adducts of hCatL-**VK-80** and hCatL-**VK-261** were stable and apparently irreversible, which supported the mechanism of Michael addition (Figure 5).

**Figure 4:**
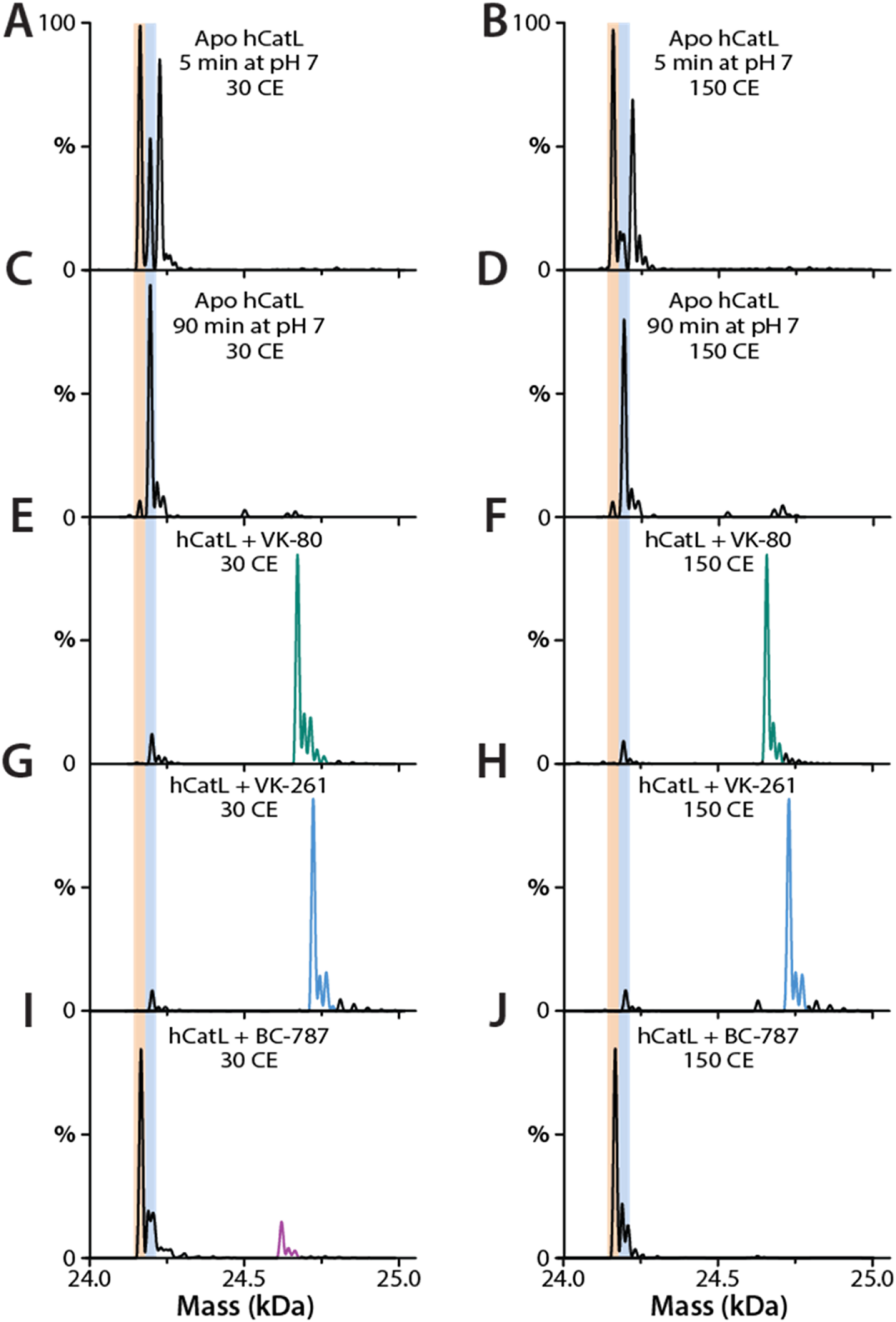
Native mass spectrometric analysis of hCatL with **VK-80**, **VK-261**, and **BC-787**. (A, B) Deconvolution of the native mass spectrum of apo hCatL 5 min after buffer exchange into 200 mM ammonium acetate (pH 7.0) (A) at 30 CE and (B) at 150 CE. (C, D) Deconvolution of the native mass spectrum of apo hCatL 90 min after buffer exchange into 200 mM ammonium acetate (pH 7.0) (C) at 30 CE and (D) at 150 CE. Peaks shaded light orange depict species of the 24,162 Da protein, and peaks shaded with light blue depict a 24,195 Da species (+33 adduct) that increased over incubation time. (E, F) Deconvolution of the native mass spectrum of hCatL with a 5-fold molar excess of **VK-80** inhibitor showed a molecular weight increase of +508 Da (green) (E) at 30 CE and (F) at 150 CE. Excess inhibitor not bound to the protein was removed during the buffer exchange. (G, H) Deconvolution of the native mass spectrum of hCatL with a 5-fold molar excess of **VK-261** showed a molecular weight increase of +563 Da (blue) (G) at 30 CE and (H) at 150 CE. (I) Deconvolution of the native mass spectrum of hCatL with a 5-fold molar excess of the **VK-787** inhibitor showed a molecular weight increase of +438 Da (pink) at 30 CE and (J) a lack of a +438 Da peak at 150 CE.

**Figure 5:**
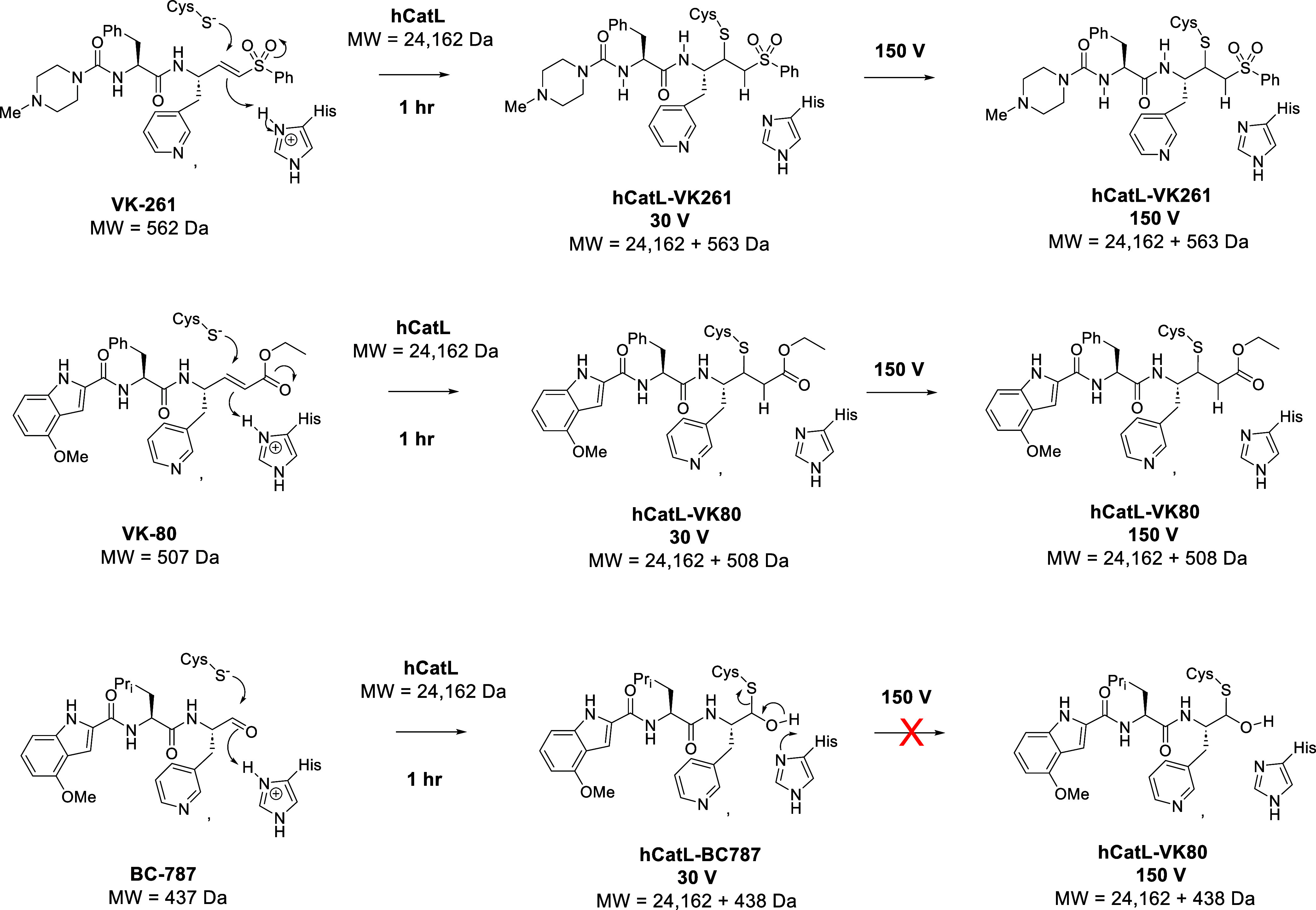
Putative mechanism of hCatL inactivators by inhibitors **VK-80** (A), **VK-261** (B), and **BC-787** (C) based on native MS mass shifts at lower CE (30.0 eV) and higher CE (150.0 eV).

Through preincubating hCatL with a 2.5-molar excess of **BC-787**, we were able to observe a +438 Da adduct at a lower energy (30 CE), corresponding to the hCatL + molecular weight of the inhibitor (Figure 4). This mass shift, in addition to preincubation data, supported the mechanism of reversible thiohemiacetal formation (Figure 5). We observed a less intense adduct peak most likely because of exchange of buffer with 200 mM ammonium acetate at pH = 7.0, which may have resulted in the reduction of hCatL activity. However, at increased energy (150 CE), we were unable to observe any adducts (Figure 4), which clearly suggested that the hCatL/BC-787 adduct was reversible at higher collision energy. This result supported the mechanism of reversible thiohemiacetal complex formation (Figure 5).

### *In Vivo* Activity of **VK-80** in a SARS-CoV-2 Infection Study

Two groups of five Balb/c mice were infected intranasally with SARS-CoV-2 (MA-10 strain; 10^5^ TCID) 2 h after intraperitoneal administration of vehicle. One group of 5 mock-challenged mice received identical doses of 40 mg/kg **VK-80** + 20 mg/kg RTV. In an earlier study, mice were infected as above and treated with vehicle containing 40 mg/kg **Nirmatrelvir** + 20 mg/kg ritonavir (RTV).

The results of this study are shown in Figure 6. Mouse weight was increased slightly for mock-infected mice treated with vehicle and **VK-80** + RTV (101 ± 1% on day 4 and 103 ± 1% on day 6). Therefore, the treatment of mice with **VK-80** and RTV (40 and 20 mg/kg, respectively) indicated little to no drug toxicity. In SARS-CoV-2-infected mice, changes in mouse weight after 4 and 6 days for vehicle-treated animals were (87 ± 4% (day 4) and 91 ± 4% (day 6)), and for infected mice treated with **VK-80** + RTV, changes in mouse weight were 90 ± 3% (day 4) and 95 ± 2% (day 6), demonstrating a partial therapeutic effect exhibited for **VK-80**. Changes in mouse weight for infected mice treated with Nirmatrelvir with ritonavir at identical doses were 93 ± 5% (day 4) and 99 ± 2% (day 6), demonstrating its higher effectiveness in this infection model. Treatment with **VK-80** significantly diminished clinical signs of SARS-CoV-2 infection compared to those in untreated, infected mice. At 4 days postinfection (dpi) and thereafter at 7 dpi, the percentage (%) of survival rate of infected mice treated with **VK-80** was 100%, like that of mock-infected mice, whereas vehicle-treated and infected mice had merely a 75% survival rate. While we have yet to optimize the formulation of **VK-80** in mice with respect to (oral) bioavailability and pharmacokinetics, these results clearly demonstrate that **VK-80** is effective in limiting disease in SARS-CoV-2-infected mice. Again, the beneficial effects seen with **VK-80** in SARS-CoV-2-infected mice are due to the inactivation of hCatL and not inhibition or inactivation of 3CL-PR.

**Figure 6:**
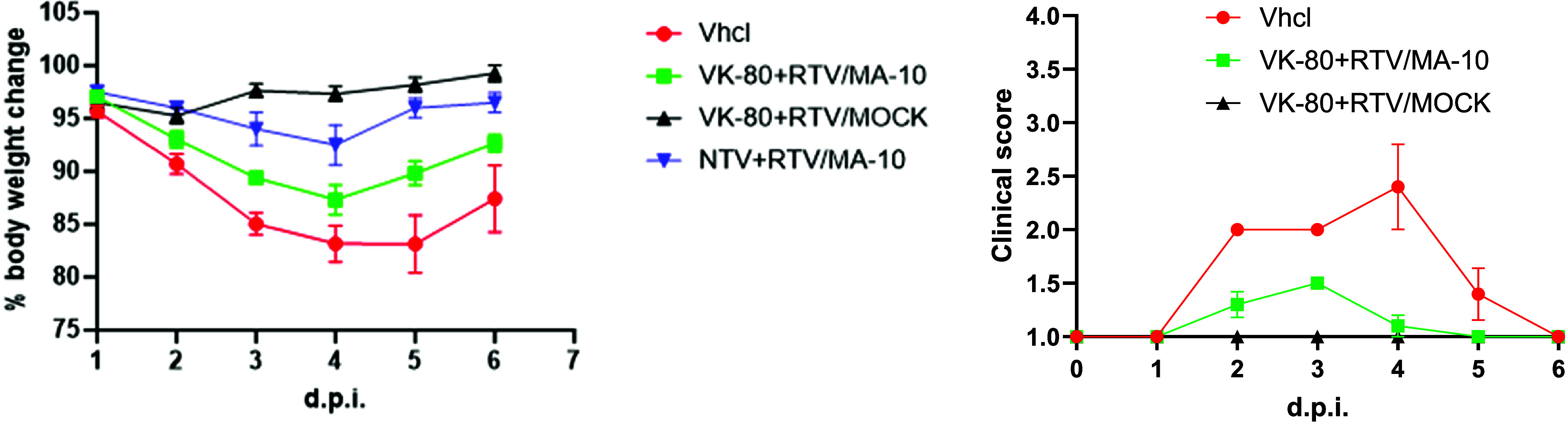
(**Left**) Changes in percent body weights of uninfected mice following 4 days treatment with vehicle + 40 mg/kg **VK-80** + 20 mg/kg ritonavir only (**VK-80** + RTV/MOCK), CoV-2-infected mice treated with vehicle only (**Vhcl + MA-10**), vehicle + 40 mg/kg **NTV** + 20 mg/kg RTV (**NTV+RTV/MA-10**), and vehicle + 40 mg/kg **VK-80** + 20 mg/kg ritonavir **(VK-80 + RTV/MA-10**). (**Right**): Changes in clinical signs in untreated (**Vhcl+MA-10**), **VK-80**-treated (**VK-80+RTV/MA-10**), and mock-infected mice (**VK-80+RTV/MOCK**).

### Ligand Docking

We also characterized the differences between the binding of **VK-80** to hCatB and hCatL. Ligand-bound structures were generated for both proteins, and several binding distances were analyzed. The docked structures displayed a distance of 3.4 Å between the β-carbon of the α,β-unsaturated ester and the thiolate of the hCatL active-site Cys_25_, as seen in Figure 7. This differed by 0.3 Å from the 3.7 Å-distance observed in the structure of hCatB. The difference in the modeled ligand placement indicates a higher likelihood of interaction between the inhibitor and Cys_25_ of hCatL than that of the Cys_29_ of hCatB. The differences in the modeled binding distance and the increased likelihood of interactions with hCatL support the finding that **VK-80** had a significantly lower *K*_i_* for hCatL than hCatB (*K*_i_* = 6.4 and 300 nM, respectively, Table 1), suggesting that the formation of the thia-Michael adduct is more easily achieved with hCatL.

**Figure 7:**
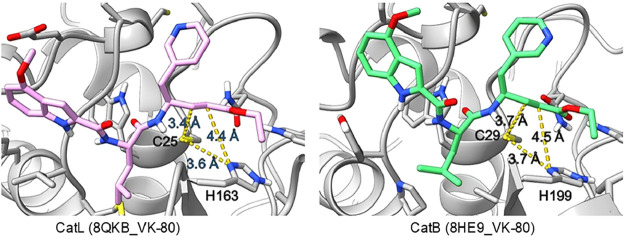
**(Left)** Predicted distances for hCatL-bound **VK-80** between the active-site Cys_25_ thiolate and the β-carbon of the unsaturated ester, and between the δ1 nitrogen of active-site His_163_ and the α-carbon of unsaturated ester using ligand docking with PDB structure 8QKB.^36^ **(Right)** Predicted interatomic distances for hCatB-bound **VK-80** between the thiolate of active-site Cys_29_ and the β-carbon of the α,β-unsaturated ester, and between the δ1 nitrogen of active-site His_199_ and the α-carbon of the unsaturated ester, using ligand docking with PDB structure 8HE9.^36^

### Chemistry

The chemical structures and synthetic routes of inhibitors 1–17 are shown in Schemes 1–8. **S1c** (Boc-5-Ox-Ala) and **S1d** (Boc-2Pyrd-Ala) were synthesized from our previously reported procedure.^23,26,34^ Boc-3-Pyr-Ala (**S1a**) and Boc-2CB-Phe (**S1b**) were acquired commercially. **S1** (a–d) were converted into respective Weinreb amides by coupling each of these with *N*,*O*-dimethyl hydroxylamine using coupling reagent HATU, resulting in yields ranging from 70 to 80%. Boc deprotection with yield > 95% was performed using 10%TFA in DCM (v/v) for 2 h (Scheme 1). Variable amino acids found at the P_3_–P_2_ groups within scaffolds were synthesized by coupling the P_3_ acid (4OMe-Ind, 7F-4OMe-Ind) to the P_2_ methyl ester (l-Leu-OMe, l-Phe-OMe, L-4F-Phe-OMe) using HATU chemistry with overall yields ranging from 60 to 80%. P_3_–P_2_-OMe were hydrolyzed to their respective acids P_3_–P_2_–OH using 1 N NaOH with yields greater than 90% (Scheme 4). Cbz-Trp and Cbz-Phe were acquired commercially. Benzylcarbamoyl-l-Phe (Cmz-Phe) (**S2c**) was synthesized by coupling benzyl amine and l-Phe-OMe to form a urea linkage using *N*,*N*′-carbonyldiimidazole (CDI), followed by hydrolysis of the methyl ester (∼63% overall yield; Scheme 2). For the synthesis of N-Me-Pip-Phe (**S3d**), l-Phe-OBn was first converted into isocyanate (**S1d**) using triphosgene and aqueous sodium carbonate and then coupled with *N*-methylpiperazine using DIPEA as base. This was followed by the removal of the benzyl ester of **S2d** using Pd/C-mediated hydrogenation in methanol that resulted in **S3d** with an overall yield of 60% (Scheme 3). Using HATU as a reagent, P_3_–P_2_ acids were coupled to the α-amino groups of P_1_-amino acids bearing a Weinreb amide, to generate P_3_–P_2_–P_1_ peptidomimetic scaffolds with yields ranging from 40 to 70% (Scheme 5). Reduction of the Weinreb amides using LiAlH_4_ afforded the desired aldehydes (Scheme 5). All peptidomimetics aldehydes were purified by prep-HPLC using an acetonitrile/water solvent system with 0.1% formic acid (v/v) and lyophilized afterward. Yields ranged from 30 to 60%.

**Scheme 1:**
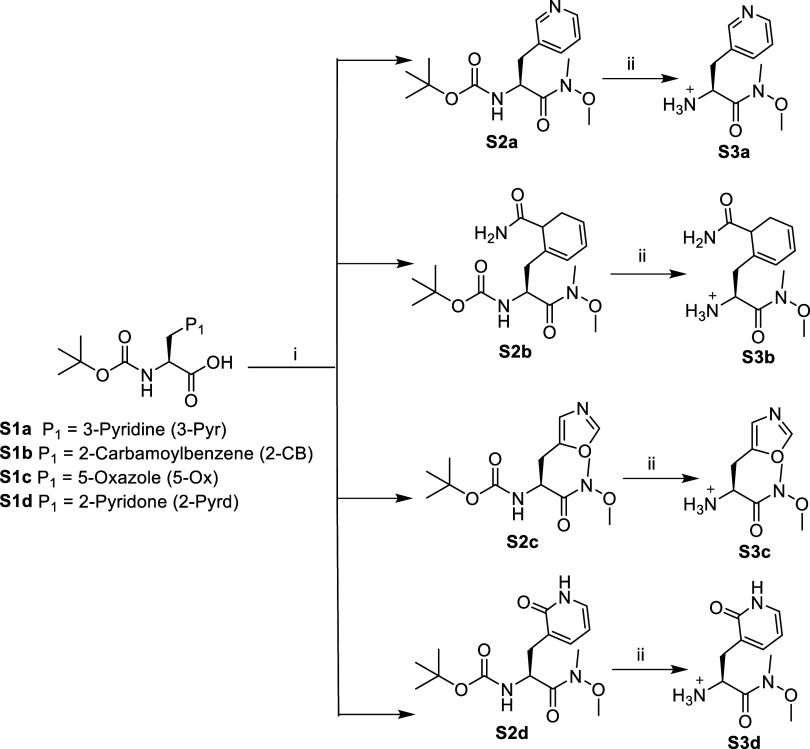
Synthesis of Compounds **S3a**, **S3b**, **S3c**, and **S3d** ^a^

a (i) *N*,*O*-Dimethyl hydroxylamine, HATU, DIPEA, DMF, 0 °C, 12 h and (ii) 10% TFA/DCM, 1 h.

**Scheme 2:**
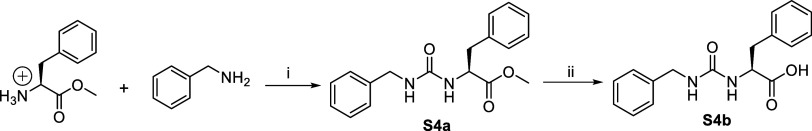
Synthesis of Compounds **S4b** ^a^

a (i) *N*,*N*′-Carbonyldiimidazole (CDI), DMF/ACN, Et_3_N, RT, 24 h and (ii) 1 N NaOH in MeOH, RT, 2 h.

**Scheme 3:**
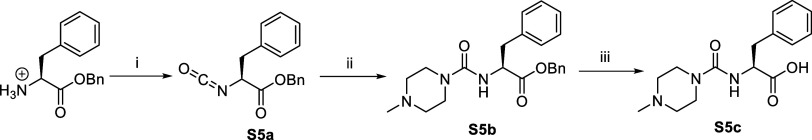
Synthesis of Compound **S5c** ^a^

a (i) Triphosgene, aq Na_2_CO_3_, DCM, 0 °C, 30 min; (ii) *N*-methylpiperazine, DIPEA, THF, RT, 24 h; (iii) H_2_, 10% Pd/C by mol, MeOH, RT, 24 h.

**Scheme 4:**
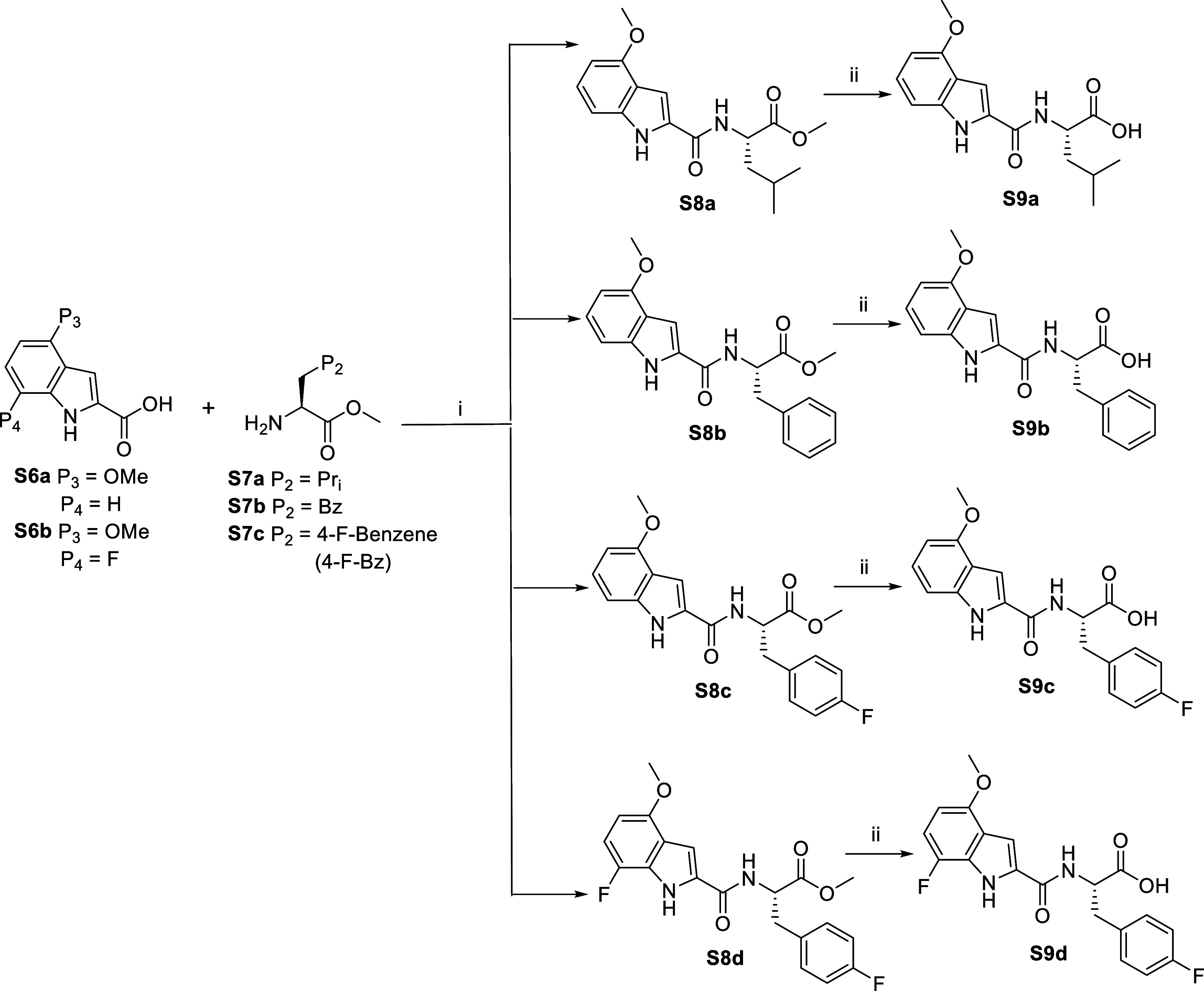
Synthesis of Compounds **S9** (a–d)^a^

a (i) HATU, DIPEA, DMF, 0 °C, 12 h and (ii) 1 N NaOH in MeOH, RT, 2 h.

**Scheme 5:**
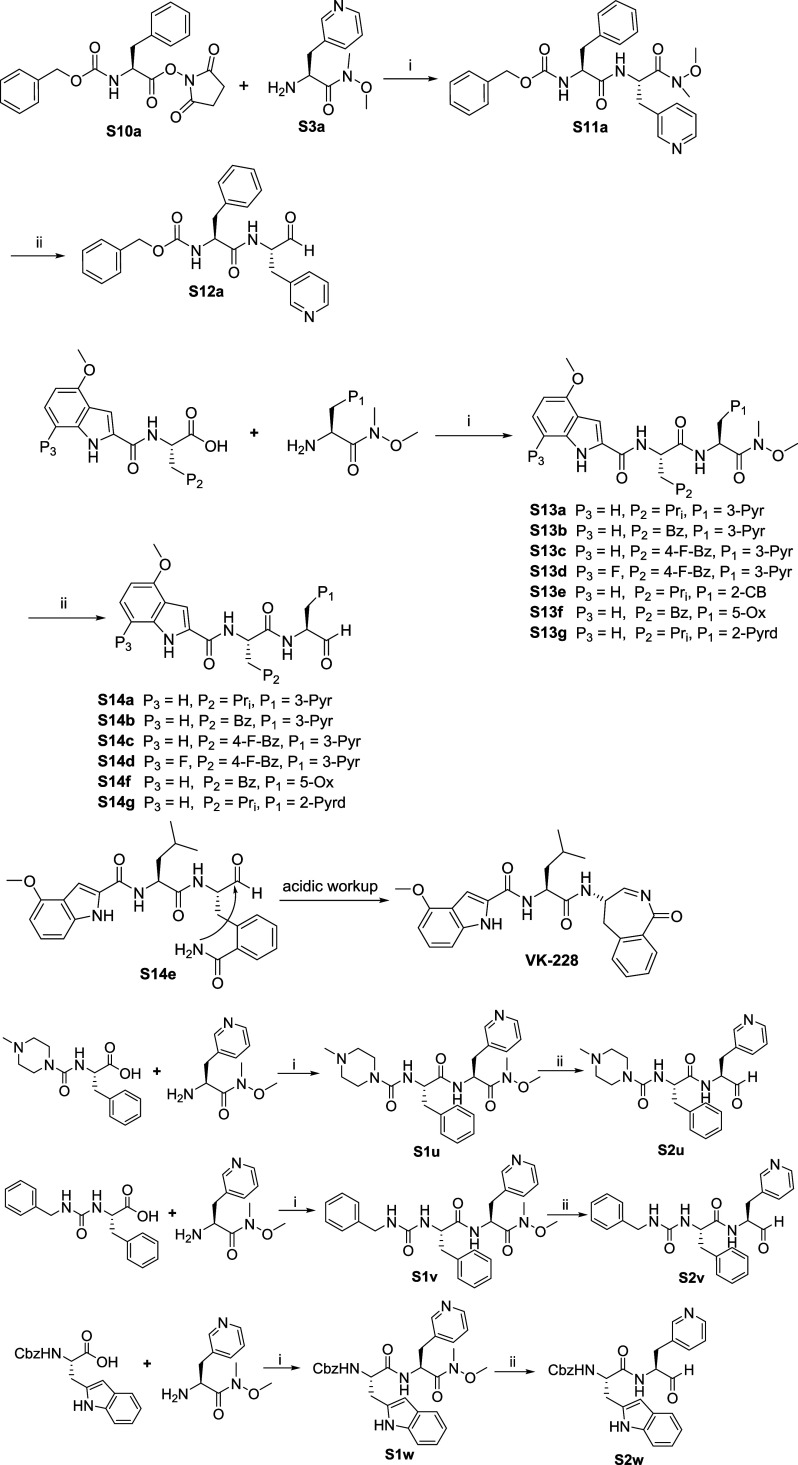
Synthesis of Peptidomimetics Aldehydes^a^

a (i) HATU, DIPEA, DMF, 0 °C, 12 h and (ii) 2 M LiAlH_4_ in THF, −10 to 0 °C, 30 min.

**Scheme 6:**
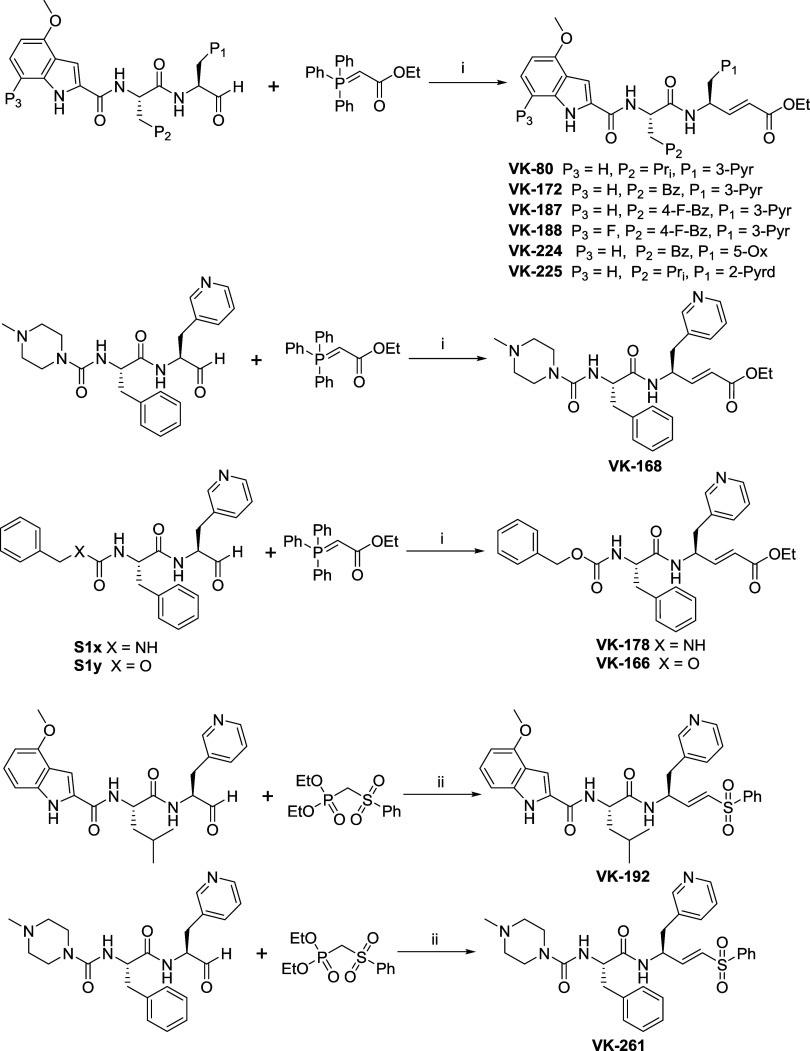
Synthesis of Peptidomimetics α,β-Unsaturated Esters and Vinyl Sulfone^a^

a (i) THF and (ii) LHMDS, dry THF, −78 to 0 °C, 30 min.

**Scheme 7:**
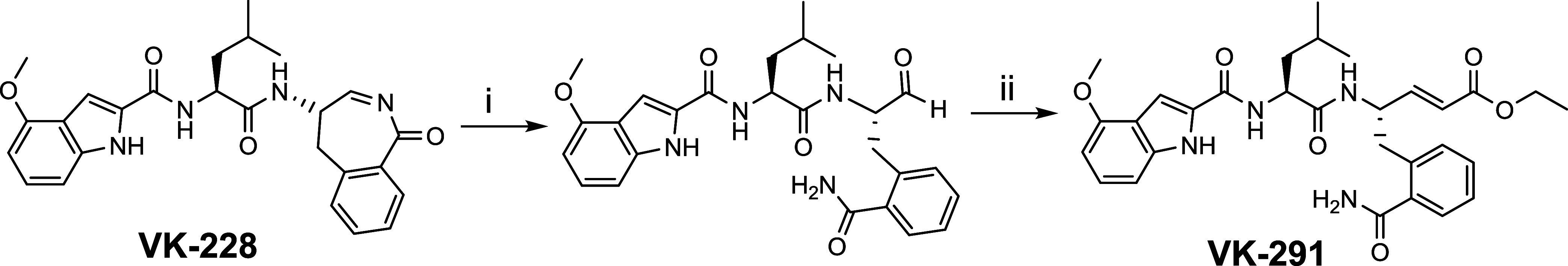
Synthesis of Compound **VK-291** ^a^

a (i) TFA, pH = 5–7, THF, 0 °C to RT, 2 h and (ii) ethyl 2-(triphenyl-λ^5^-phosphaneylidene) acetate, 24 h, RT.

Peptidomimetics aldehydes were converted to their respective α,β-unsaturated ester using the Wittig reaction with ethyl 2-(triphenyl-λ^5^-phosphaneylidene) acetate in THF with yields ranging from 85 to 95% (Scheme 6). Both the (*E*)-isomer (yield 55:70%) and (*Z*)-isomer (yield 30:45%) were isolated by prep-HPLC purification. During prep-HPLC purification using the ACN/water solvent system with 0.1% (v/v) formic acid, the (*Z*)-isomer was found to be relatively more polar and with a lower elution time than the (*E*)-isomer, which led to the effective separation of both isomers. **VK-192** and **VK-261** were synthesized with yields ranging from 50 to 70% using the Horner–Wadsworth–Emmons reaction (Scheme 6). ((Phenylsulfonyl)­methyl) phosphonate was activated using the base LHMDS at −78 °C for 10–20 min, followed by the addition of a THF solution containing the appropriate peptidomimetic aldehyde, and the reaction was stirred for 30–40 min (Scheme 6). Both (*E*)-isomer (yield, 60–80%) and (*Z*)-isomer (yield, 20–40%) were isolated using prep-HPLC purification. Similar to the polarity difference of the (*E*) and (*Z*) isomers of the result in vinyl esters, the (*Z*) isomer of the vinyl sulfone was found to be more polar and have a lower elution time compared to the (*E*)-isomer of the vinyl sulfone. **VK-228** was converted to **VK-291** (yield ∼ 10%) by treating it with ethyl 2-(triphenyl-λ^5^-phosphaneylidene) acetate at pH 5–7, which was achieved by adding a few drops of TFA. The poor yield most likely resulted from acidic pH since the Wittig ylide is protonated and deactivated at acidic pH (Scheme 7). **SM-1** was converted to **BC-803** with a yield of ∼34% by dehydrating the primary amide of SM-1 to afford the nitrile using Burgess reagent in DCM (stirred at 25 °C for 7 h) (Scheme 8).

**Scheme 8:**
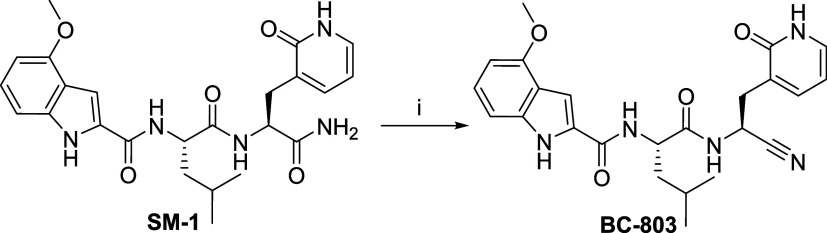
Synthesis of **BC-803** ^a^

a (i) Burgess reagent, DCM, 25 °C, 7 h.

## Conclusions

In this study, we have shown that tripeptide α,β-unsaturated ethyl esters are irreversible inactivators of human cathepsin L, while they are poor, reversible inhibitors of the homologous cysteine protease, human cathepsin B, and with few exceptions, inactive vs 3CL-PR. For compounds that differed structurally only by the enoate (**VK-80**, **VK-168**) or vinyl sulfone warhead (**VK-192** and **VK-261**), those with vinyl sulfone warheads (**VK-192** and **VK-261**) were significantly more selective for hCatL over hCatB than the enoates **VK-80** and **VK-168**. The enoates are considered to be mildly reactive electrophiles such that they likely only react with active-site thiolate groups and not thiols.^21,22,27,38^ Despite the fact that a vinyl sulfone is approximately 7-fold more electrophilic than a vinyl ester with a thiolate group,^27^ here we observed that the inactivation kinetics of compounds containing the two warheads do not reflect this difference in electrophilicity; indeed, the enoate compounds have similar or better activity than their counterparts with vinyl sulfone groups. Native mass spectrometric data corroborated the kinetic irreversibility observed for **VK-80** and **VK-261** with hCatL as their enzyme-inactivator adducts remained present under high-energy conditions. Enoate inactivators of hCatL identified here are in some cases among the most potent anti-SARS-CoV-2 compounds yet characterized and, given their selectivity for hCatL vs hCatB, may prove useful as anti-COVID-19 agents, as suggested by the favorable *in vivo* activity of **VK-80**. This is reflected in the anti-SARS-CoV-2 activity of **VK-80** in a mouse model of SARS-CoV-2 infection. Lead optimization of some of these compounds is likely to afford drug-quality candidates for the treatment of COVID-19.

## Experimental Section

### Materials

Deuterium oxide (99.9+ % gram atom D) was purchased from Cambridge Isotope Laboratories. Deuterium chloride (35% w/w, 99% D), sodium deuterium oxide (40% w/w, 99% D), and sodium acetate were purchased from Millipore Sigma. Disodium EDTA was obtained from Fluka. Dithiothreitol and CHAPS were purchased from Goldbio. Cbz-Leu-Arg-AMC was obtained from R&D Systems. All other chemicals were of reagent grade or better and used without further purification. The irreversible covalent inactivator 4-methyl-*N*-[(1*S*)-2-oxo-2-[[(1*S*)-1-(2-phenylethyl)-3-(phenylsulfonyl)-2-propen-1-yl]­amino]-1-(phenylmethyl)­ethyl]-1-piperazinecarboxamide (**K11777**, MePip-Phe-hPhe-VSPh) was prepared as previously described.^27^

### General Considerations of Synthesis

Starting materials and reagents were purchased from commercial sources and used without further purification. Reactions were carried out under an inert atmosphere of nitrogen unless otherwise specified. The progress of the reactions was monitored using thin-layer chromatography (TLC) and LC-MS (liquid chromatography–mass spectrometry) analysis, by employing an HPLC-MS (UltiMate 3000 equipped with a diode array coupled to a MSQ Plus Single Quadrupole Mass Spectrometer, ThermoFisher Scientific) using electrospray positive and negative ionization detectors. HPLC conditions used: Column: Phenomenex Luna 5 μm C18(2) 100 Å, 4.6 mm × 50 mm, Mobile phase A: water with 0.1% formic acid (v/v). Mobile phase B: MeCN with 0.1% formic acid (v/v). Temperature: 25 °C. Gradient: 0–100% B over 6 min, then a 2 min hold at 100% B. Flow rate: 1 mL/min. Detection: MS and UV at 254 nm.

Compounds were purified by flash column chromatography (FCC) on silica gel (200–300 mesh) with different solvent systems. Most of the final compounds were purified by semipreparative HPLC (Prep-HPLC) on the same UltiMate 3000 HPLC system, which was connected to a fraction collector. The typical settings for Prep-HPLC were as follows: Column used: Phenomenex Luna 5 μm C18(2) 100 Å, 21.2 mm × 250 mm; mobile phases A and B were the same as those for analytical HPLC; method: gradient elution at 10–100% B over 25 min, then isocratic elution at 100% B for 5 min; flow rate: 21.2 mL/min. NMR spectra were obtained in DMSO-*d*_6_ at 400 MHz at 298 K on a Bruker Avance III NanoBay console with an Ascend magnet. The following abbreviations were utilized to describe peak patterns when appropriate: br = broad, s = singlet, d = doublet, q = quartet, t = triplet, and m = multiplet. The compounds used for testing in assays and biological studies were determined to be >95% pure as evaluated by their proton NMR spectra and their HPLC/MS based on ultraviolet detection at 254 nm.

#### Synthesis of Intermediates **S3a** and **S3b**

Boc-l-3-pyridyl alanine (**S1a**, Boc-3Pyr-Ala), Boc-l-2-carbamoylphenylalanine (**S1b**, Boc-2CB-Phe), and Boc-l-5-Oxazolyl alanine (**S1c**, Boc-5-Ox-Ala) were acquired commercially from Combi-Blocks, Peptech Corporations, and ChemSpace, respectively. Boc-2-pyridnoylalanine (**S1d**, Boc-2-Pyrd-Ala) was synthesized using the reported procedure.^23,26,34^ Intermediates **S3­(a–d)** were synthesized according to Scheme 1.

##### *tert*-Butyl (*S*)-(1-(Methoxy­(methyl)­amino)-1-oxo-3-(pyridin-3-yl)­propan-2-yl)­carbamate (**S2a**, P_1_ = 3-Pyridine)

Boc-3Pyr-Ala (**S1a**) was coupled with *N*,*O*-dimethyl hydroxylamine to convert it to a Weinreb amide. **S1a** (10 mmol, 1 equiv, 2660 mg) and HATU (12 mmol, 1.2 equiv, 4560 mg) were dissolved in 5 mL of DMF in a 25 mL round-bottom (RB) flask, which was cooled to 0 °C. To this ice-cold solution, DIPEA (20 mmol, 2 equiv, 3.6 mL) was added slowly with stirring of the solution for 2 min to activate acid into an HOAt ester. *N*,*O*-Dimethyl hydroxylamine hydrochloride (12 mmol, 1.2 equiv, 1180 mg) was first neutralized with DIPEA (12 mmol, 1 equiv, 2.1 mL) and then added into the reaction mixture at 0 °C, and stirred overnight. Reaction completion was verified with TLC and LC-MS. Excess water was added to the mixture and the product was extracted three times with EtOAc (100 × 3 mL). The organic layer was washed three times with 10% Na_2_CO_3_ solution (w/v) (100 × 3 mL) and with brine solution (100 × 3 mL). The organic layer was dried by adding anhydrous Na_2_SO_4_ and concentrated under a vacuum. The compound **S2a** was purified by silica gel column chromatography and eluted using a 50–60% gradient of ethyl acetate (EA)/hexane solvent system (v/v). Compound **S2a** after concentrating under vacuum resulted in approximately 79% yield (2450 mg). **S2a** (LC-MS: *t*_R_ = 2.66 min); C_15_H_23_N_3_O_4_ (*m*/*z* calcd 309.2, found 310.3 (M + H)). Similarly, **S2b**, **S2c**, and **S2d** were prepared with yields 80% (2800 mg), 85% (2550 mg), and 75% (2650 mg), respectively. **S2b** (LC-MS: *t*_R_ = 3.36 min); C_17_H_25_N_3_O_5_ (*m*/*z* calcd 351.2, found 352.2 (M + H), 251.3 (M-Boc)). **S2c** (LC-MS: *t*_R_ = 3.15 min); C_13_H_21_N_3_O_5_ (*m*/*z* calcd 299.1, found 300.2 (M + H), 199.2 (M-Boc)). **S2d** (LC-MS: *t*_R_ = 3.01 min); C_15_H_23_N_3_O_5_ (*m*/*z* calcd 325.2, found 326.3 (M + H)).

In a 250 mL RB flask, compound **S2a** (7.9 mmol, 1 equiv, 2450 mg) was dissolved in 4 mL of TFA and 16 mL of DCM, which was stirred for 2 h. After reaction completion, DCM was removed under vacuum to give a white gummy compound. The compound was solubilized in 50 mL of water and lyophilized, resulting in a white powder (TFA salt of **S3a**) (2500 mg, yield > 95%). TFA salts of **S3b**, **S3c**, and **S3d** were prepared in a similar manner with yields >95% and used further without purification.

##### (Benzylcarbamoyl)-l-phenylalanine (**S4b**)

In a 250 mL RB flask, *N*,*N*′-carbonyldiimidazole (CDI) (3.28 g, 20.2 mmol, 1.09 equiv) and l-phenylalanine methyl ester hydrochloride (4.01 g, 18.6 mmol, 1 equiv) were dissolved in 80 mL of ACN and 15 mL of DMF. To this solution, Et_3_N (5.2 mL, 37.2 mmol, 2 equiv) was added slowly, and the reaction was stirred for 30 min at RT. Afterward, benzyl amine (2.03 mL, 1 equiv) was added to the reaction mixture and stirred for more than 25 h. After reaction completion, ACN was evaporated under vacuum, and excess water was added to the solution. Compound (**S4a**) was extracted with EtOAc three times (3 × 100 mL). The organic layer was washed with 0.1 N HCl three times (3 × 100 mL) and washed with brine solution three times (3 × 100 mL). The compound was dried by adding anhydrous Na_2_SO_4_ and concentrated under a vacuum, which resulted in a white gummy solid. The compound (**S4a**) was purified using silica gel column chromatography using increasing gradient of EtOAc in an EtOAc/Hexane (v/v) solvent system and eluted at 25–30% EtOAc/Hexane (v/v). The compound was concentrated under vacuum, resulting in an approximately 70% yield (3.9 g). Compound (**S4a**) (3.9 g, 12.6 mmol) was subjected to hydrolysis by stirring it in 10 mL of MeOH and 40 mL of 1 N NaOH solution for 2 h. After reaction completion was confirmed by TLC and LC-MS, MeOH was evaporated, and the solution was acidified by the addition of 0.1 N HCl solution until the pH of the solution reached below 4. The compound was extracted three times with EtOAc (3 × 50 mL) and washed with brine solution three times (3 × 100 mL). Evaporation of EtOAc resulted in a white powdered compound (**S2c**) with a yield > 90% (3.45 g), which is used without further purification. **S4b** (LC-MS: *t*_R_ = 4.4 min); C_17_H_18_N_2_O_3_ (*m*/*z* calcd 298.3, found 299.1 (M + H)).

##### (4-Methylpiperazine-1-carbonyl)-l-phenylalanine (**S5c**)

In a 250 mL round-bottom flask, benzyl l-phenylalaninate (10 mmol, 1 equiv, 2250 mg) is dissolved in 40 mL of DCM containing aqueous sodium carbonate (10 mL of 10% Na_2_CO_3_ in water (w/v)), and the mixture is cooled to 0 °C in ice. To this solution, triphosgene (4 mmol, 0.4 equiv, 670 μL) is added slowly, and the reaction mixture is stirred for 30 min at 0 °C. After reaction completion, 200 mL of water is added to the mixture and benzyl (*S*)-2-isocyanato-3-phenylpropanoate (**S5a**) was extracted 3 times with EtOAc (50 × 3 mL). The organic layer was washed three times with 0.1 N HCl (30 × 3 mL), followed by washing three times with 10% Na_2_CO_3_ (w/v) (30 × 3 mL) and finally washed with brine three times (50 × 3 mL). The organic layer was dried over Na_2_SO_4_ and concentrated under a vacuum. The concentrated compound was dissolved in 10 mL THF containing *N*-methylpiperizine (6 mmol, 1.5 equiv, 668 μL). To this solution, DIPEA (8 mmol, 2 equiv, 1500 μL) was added slowly, and the reaction was stirred for 24 h. After reaction completion was confirmed by TLC and LC-MS, 200 mL of water was added to the mixture. The compound was extracted three times with EtOAc (3 × 50 mL). The organic layer was washed five times with water (5 × 3 mL), followed by washing three times with 10% Na_2_CO_3_ (3 × 50 mL) (w/v) and 3 times with brine (3 × 50 mL). Finally, the organic layer was dried over Na_2_SO_4_ and concentrated under vacuum. The compound (**S5b**) was purified using silica gel column chromatography with increasing polarity using MeOH/DCM solvent system and eluted at 2–4% methanol (v/v) in methanol/DCM, the same question as above. Evaporation of solvents resulted in an approximately 62% yield (950 mg). **S5b** (LC-MS: *t*_R_ = 3.42 min); C_22_H_27_N_3_O_3_ (*m*/*z* calcd 381.2, found 382.2 (M + H), 383.3 (M + 2H)).

In a 250 mL RB flask, the compound was dried under a high vacuum. Pd/C (20 mol %, 150 mg) was added to the flask under nitrogen. To this reaction mixture was added 25 mL of dry MeOH and the reaction was stirred under hydrogen for 12 h. The benzyl ester was hydrolyzed to acid (**S5c**). The Pd/C was filtered out through Celite. The compound was concentrated *in vacuo* and used further without purification. **S5c** (LC-MS: *t*_R_ = 1.8 min); C_15_H_21_N_3_O_3_ (*m*/*z* calcd 291.1, found 290.2 (M – H), 314.2 (M + Na)).

##### (4-Methoxy-1*H*-indole-2-carbonyl)-l-phenylalanine (P_2_ = Bz) (**S9b**)

In a 25 mL round-bottom flask, **S6a** (5 mmol, 5 equiv, 955 mg) and HATU (6 mmol, 1.2 equiv, 2280 mg) were dissolved in 4 mL of DMF and cooled to 0 °C. To this solution, DIPEA (10 mmol, 2 equiv, 1.6 mL) was added slowly. l-Phe methyl ester (HCl salt) **S7b** (6 mmol, 1.2 equiv, 1278 mg) was first dissolved in 1 mL of DMF and 1 mL of DIPEA. This solution was added slowly to the reaction mixture and stirred overnight. After completion of the reaction, 200 mL of water was added to the reaction mixture, followed by the addition of 100 mL EtOAc. The product compound was extracted with EtOAc (3 × 100 mL). The organic layer was washed three times with 0.1 N HCl solution (3 × 100 mL) and three times with 10% (w/v) Na_2_CO_3_ solution (3 × 100 mL), and finally washed with brine (3 × 100 mL). The organic layer was dried over Na_2_SO_4_. The product was purified by silica gel-based column chromatography using a EtOAc/hexane solvent system with elution at (40% EtOAc in hexane, (v/v)), the same question as above. Solvent was evaporated to give a white powder (**S8b**); yield 1.2 g (∼70%). In a 250 mL round-bottom flask, whole compound **S8b** (1.2 g, 3.5 mmol) was dissolved in 30 mL of MeOH. 50 mL of 1 N NaOH in water was added slowly to the reaction mixture, followed by stirring for 2 h. After complete hydrolysis, as confirmed by TLC and LC-MS, the MeOH was removed under vacuum, and the reaction mixture was acidified with 1 N HCl solution. The mixture was extracted three times with EtOAc (3 × 100 mL) and washed three times with brine (3 × 100 mL). EtOAc was removed under vacuum to give a pure white powder (**S9b**; ∼1150 mg, >95%). (LC-MS: *t*_R_ = 4.49 min); C_19_H_18_N_2_O_4_ (*m*/*z*: calcd 338.1; found 339.1 (M + H)). Similarly, intermediates **S9** (**a**–**d**) were prepared.

##### 4-Methoxy-*N*-((*S*)-1-(((*S*)-1-(methoxy­(methyl)­amino)-1-oxo-3-(pyridin-3-yl)­propan-2-yl)­amino)-1-oxo-3-phenylpropan-2-yl)-1*H*-indole-2-carboxamide (**S13b**, P_1_ = 3-Pyr)

In a 25 mL round-bottom flask, **S9b** (1 mmol, 1 equiv, 339 mg) and HATU (1.2 mmol, 1.2 equiv, 456 mg) were dissolved in 5 mL of DMF and cooled to 0 °C. The acid was converted to its HOAt ester by adding DIPEA (2 mmol, 2 equiv, 0.35 mL) into the reaction mixture, followed by stirring for 1–2 min. The TFA salt of **S3a** (1.2 mmol, 1.2 equiv, 407 mg) was neutralized with a few drops of DIPEA and dissolved in 1 mL of DMF. The **S3a** solution was added dropwise into a reaction mixture with the activated ester at 0 °C, which was stirred for 12 h. After completion of the reaction, the mixture was added to 100 mL of water, followed by the addition of 100 mL EtOAc. The product was extracted with EtOAc (3 × 100 mL). The organic layer was washed three times with a 10% Na_2_CO_3_ solution (3 × 100 mL) and washed with brine (3 × 100 mL). The organic layer was dried over Na_2_SO_4_. The compound was purified by Silica gel column chromatography using increasing polarity of EtOAc/Hexane solvent system and eluted at 90–100% EA in Hexane, v/v. The solvent was evaporated to give a yellowish gummy compound **S13b** (250 mg, ∼50%). (LC-MS: *t*_R_ = 3.82 min); C_29_H_31_N_5_O_5_, *m*/*z*: calcd 529.2; found 530.2 (M + H). Similarly, intermediates **S13** (**a**–**g**) were prepared.

##### 4-Methoxy-*N*-((*S*)-1-oxo-1-(((*S*)-1-oxo-3-(pyridin-3-yl)­propan-2-yl)­amino)-3-phenylpropan-2-yl)-1*H*-indole-2-carboxamide (**S14b**)

In a 100 mL round-bottom flask, **S13b** (0.47 mmol, 1 equiv, 250 mg) was dissolved in 5 mL dry THF and cooled to −10 °C. Under nitrogen, 2 M LiAlH_4_ in THF (1 mmol, 2 equiv, 500 μL) was added dropwise into the reaction mixture. The reaction mixture was stirred for 20–30 min. After completion of the reaction, it was slowly quenched with a few drops of 0.1 N HCl solution at 0 °C. The THF was removed under vacuum, excess water was added to the mixture, and the reaction was quenched by adding aq 10% sodium carbonate (w/v). The aldehyde was extracted with EtOAc (3 × 50 mL). The organic layer was washed with brine solution (3 × 50 mL). The organic layer was concentrated under vacuum, and the residue was dissolved in 5 mL DMF. Compound **S14b** was purified by preparative HPLC using a linear gradient of (40–60% acetonitrile in water (v/v)) with 0.1% formic acid (v/v) in the mobile phase. Lyophilization resulted in a white powder; **S14b** (yield: 80 mg, 34%). (LC-MS: *t*_R_ = 3.35 min; C_27_H_26_N_4_O_4_, *m*/*z*: calcd 470.1; found 470.2 (M)).

##### 4-Methoxy-*N*-((*S*)-4-methyl-1-oxo-1-(((*S*)-1-oxo-4,5-dihydro-1*H*-benzo­[*c*]­azepin-4-yl)­amino)­pentan-2-yl)-1*H*-indole-2-carboxamide (**VK-228**)

**S13e** (0.5 mmol, 1 equiv, 268 mg) was dissolved in 5 mL of dry THF and cooled to −10 °C. Under nitrogen, 2 M LiAlH_4_ in THF (1 mmol, 2 equiv, 500 μL) was added dropwise into the reaction mixture. The reaction was stirred for 20–30 min. After completion of the reaction, it was slowly quenched with 20 mL of 0.1 N HCl solution at 0 °C and the solution was stirred for 30 min. THF was removed by evaporation, and excess water was added to the mixture. The compound **VK-228** was extracted with EtOAc (3 × 50 mL). The organic layer was washed with brine solution (3 × 50 mL). The organic layer was concentrated under reduced pressure and dissolved in 5 mL DMF. The compound was purified by preparative HPLC using a linear gradient of (50–75% acetonitrile in water (v/v)) with 0.1% formic acid (v/v) in the mobile phase. Lyophilization resulted in a white powder; **VK-228** (yield: 120 mg, 52%). (LC-MS: *t*_R_ = 4.33 min; C_26_H_28_N_4_O_4_, *m*/*z*: calcd 460.2; found 461.2 (M + H)). The cyclized imine product was confirmed by ^1^H and ^13^C NMR. ^1^H NMR (400 MHz, DMSO-D_6_) δ 0.89 (m, 6H), 1.52 (m, 1H), 1.71 (td, 2H), 2.76 (m, 1H), 2.94 (m, 1H), 3.89 (s, 3H), 4.12 (m, 1H), 4.51 (m, 2H), 6.12 (dd, 1H), 6.51 (d, 1H), 7.02 (dd, 1H), 7.11 (m, 2H), 7.23 (dd, 1H), 7.33 (m, 3H), 7.51 (m, 1H), 8.13 (d, 1H), 8.20 (d, 1H), 8.30 (dd, 1H), 11.54 (d, 1H). ^13^C NMR (100 MHz, DMSO-D_6_) δ (21.97, 23.55, 24.89, 36.50, 51.88, 55.57, 57.89, 79.81, 99.75, 101.54, 105.86, 118.57, 124.86, 127.28, 128.26, 129.88, 130.48, 131.07, 135.70, 135.83, 136.93, 138.31, 154.12, 161.20, 161.34, 169.72, 172.58).

#### Synthesis of α,β-Unsaturated Esters

##### Ethyl (*S*,*E*)-4-((*S*)-2-(4-Methoxy-1*H*-indole-2-carboxamido)-3-phenylpropanamido)-5-(pyridin-3-yl)­pent-2-enoate (**VK-172**)

In a 25 mL round-bottom flask, **S14b** (0.2 mmol, 1 equiv, 94 mg) was dissolved in 5 mL of THF containing ethyl 2-(triphenyl phosphaneylidene)­acetate (0.22 mmol, 1.1 equiv, 77 mg), and the reaction was stirred overnight at room temperature. After reaction completion, THF was removed under vacuum and the resulting solid was dissolved in 4 mL DMF, which was eluted by preparative HPLC using a linear gradient of (45–65% acetonitrile in water (v/v)) with 0.1% formic acid (v/v) in the mobile phase. Lyophilization resulted in a white powder of **VK-172** (yield: 95 mg, 90%). (LC-MS: *t*_R_ = 4.11 min; C_31_H_32_N_4_O_5_, *m*/*z*: calcd 541.2; found 542.3 (M + H)). ^1^H NMR (400 MHz, DMSO-D_6_) δ 1.21 (td, 3H), 2.75 (m, 2H), 2.93 (m, 1H), 3.01 (m, 1H), 3.89 (d, 3H), 4.13 (m, 2H), 4.69 (m, 1H), 5.78 (dd, 1H), 5.97 (dd, 1H), 6.50 (dd, 1H), 7.12 (m, 11H), 7.67 (m, 1H), 8.33 (m, 1H), 8.41 (m, 1H), 8.48 (m, 2H), 11.47 (dd, 1H).

##### Ethyl (*S*,*E*)-4-((*S*)-2-(4-Methoxy-1*H*-indole-2-carboxamido)-4-methylpentanamido)-5-(pyridin-3-yl)­pent-2-enoate (**VK-80**)

(Yield: 160 mg, 88%). (LC-MS: *t*_R_ = 3.97 min; C_28_H_34_N_4_O_5_, *m*/*z*: calcd 507.2; found 508.3 (M + H)). ^1^H NMR (400 MHz, DMSO-D_6_) δ 0.85 (m, 6H), 1.19 (dt, 4H), 1.40 (m, 1H), 1.59 (m, 1H), 2.84 (dd, 1H), 3.00 (m, 1H), 3.89 (d, 3H), 4.11 (m, 2H), 4.42 (m, 1H), 4.74 (m, 1H), 5.86 (dd, 1H), 6.51 (dd, 1H), 6.92 (m, 1H), 7.03 (m, 1H), 7.10 (td, 1H), 7.23 (m, 1H), 7.33 (dd, 1H), 7.66 (dt, 1H), 8.20 (d, 1H), 8.36 (m, 3H), 11.53 (dd, 1H).

##### Ethyl (*S*,*E*)-4-((*S*)-2-(((Benzyloxy)­carbonyl)­amino)-3-phenylpropanamido)-5-(pyridin-3-yl)­pent-2-enoate (**VK-166**)

(Yield: 90 mg, 91%). (LC-MS: *t*_R_ = 4.04 min; C_29_H_31_N_3_O_5_, *m*/*z*: calcd 502.2; found 503.3 (M + H)). ^1^H NMR (400 MHz, DMSO-D_6_) δ 1.21 (td, 3H), 2.56 (dt, 1H), 2.71 (dd, 1H), 2.82 (m, 1H), 2.98 (m, 1H), 3.29 (m, 2H), 4.20 (m, 3H), 4.96 (d, 2H), 5.97 (dd, 1H), 6.84 (dd, 1H), 6.95 (dd, 1H), 7.26 (m, 9H), 7.42 (d, 1H), 7.64 (dt, 1H), 8.22 (d, 1H), 8.31 (d, 1H), 8.42 (m, 2H).

##### Ethyl (*S*,*E*)-4-((*S*)-2-(4-Methylpiperazine-1-carboxamido)-3-phenylpropanamido)-5-(pyridin-3-yl)­pent-2-enoate (**VK-168**)

(Yield 30 mg, 80%). (LC-MS: *t*_R_ = 2.70 min; C_27_H_35_N_5_O_4_, *m*/*z*: calcd 493.3; found 494.3 (M + H)). ^1^H NMR (400 MHz, DMSO-D_6_) δ 1.20 (m, 3H), 2.15 (m, 8H), 2.70 (m, 3H), 2.98 (m, 2H), 3.20 (m, 5H), 3.66 (m, 1H), 4.15 (m, 3H), 4.71 (s, 1H), 5.99 (dd, 1H), 6.48 (dd, 1H), 6.89 (m, 1H), 7.16 (m, 2H), 7.21 (t, 2H), 7.26 (m, 1H), 7.65 (dt, 1H), 8.39 (q, 1H), 8.43 (m, 1H).

##### Ethyl (*S*,*E*)-4-((*S*)-2-(3-Benzylureido)-3-phenylpropanamido)-5-(pyridin-3-yl)­pent-2-enoate (**VK-178**)

(Yield 55 mg, 78%). (LC-MS: *t*_R_ = 4.02 min; C_29_H_32_N_4_O_4_, *m*/*z*: calcd 500.2; found 501.2 (M + H)). ^1^H NMR (400 MHz, DMSO-D_6_) δ 1.22 (t, 3H), 2.77 (m, 2H), 2.97 (m, 1H), 3.26 (s, 3H), 4.16 (m, 4H), 4.38 (td, 1H), 5.70 (dd, 1H), 6.08 (m, 1H), 6.47 (q, 1H), 6.80 (dd, 1H), 7.21 (m, 11H), 7.63 (dt, 1H), 8.22 (d, 1H), 8.35 (m, 1H), 8.43 (m, 1H).

##### Ethyl (*S*,*E*)-4-((*S*)-3-(4-Fluorophenyl)-2-(4-methoxy-1*H*-indole-2-carboxamido)­propanamido)-5-(pyridin-3-yl)­pent-2-enoate (**VK-187**)

(Yield 15 mg, 95%). (LC-MS: *t*_R_ = 4.26 min; C_31_H_31_FN_4_O_5_, *m*/*z*: calcd 558.2; found 559.2 (M + H)). ^1^H NMR (400 MHz, DMSO-D_6_) δ 1.14 (t, 3H), 2.55 (dd, 1H), 2.66 (dd, 2H), 2.92 (s, 1H), 2.98 (dd, 1H), 3.20 (s, 2H), 3.81 (s, 3H), 4.53 (ddd, 1H), 4.69 (m, 1H), 5.90 (dd, 1H), 6.42 (d, 1H), 6.92 (m, 2H), 7.01 (m, 3H), 7.22 (m, 3H), 7.61 (dt, 1H), 8.37 (m, 3H), 11.39 (d, 1H).

##### Ethyl (*S*,*E*)-4-((*S*)-2-(7-Fluoro-4-methoxy-1*H*-indole-2-carboxamido)-3-(4-fluorophenyl)­propanamido)-5-(pyridin-3-yl)­pent-2-enoate (**VK-188**)

(Yield 20 mg, 89%). (LC-MS: *t*_R_ = 4.37 min; C_31_H_30_F_2_N_4_O_5_, *m*/*z*: calcd 576.2; found 577.1 (M + H)). ^1^H NMR (400 MHz, DMSO-D_6_) δ 1.14 (t, 3H), 2.62 (m, 3H), 2.98 (dd, 1H), 3.20 (s, 1H), 3.80 (s, 3H), 4.06 (qd, 2H), 4.56 (ddd, 1H), 4.71 (m, 1H), 5.89 (dd, 1H), 6.33 (dd, 1H), 6.86 (ddd, 2H), 6.99 (m, 2H), 7.22 (m, 4H), 7.61 (dt, 1H), 8.34 (dd, 1H), 8.41­(m, 2H), 8.46 (d, 1H), 11.87 (d, 1H).

##### Ethyl (*S*,*E*)-4-((*S*)-2-(((Benzyloxy)­carbonyl)­amino)-3-(1*H*-indol-2-yl)­propanamido)-5-(pyridin-3-yl)­pent-2-enoate (**VK-223**)

(Yield 115 mg, 88%). (LC-MS: *t*_R_ = 4.07 min; C_31_H_32_N_4_O_5_, *m*/*z*: calcd 540.2; found 541.2 (M + H)). ^1^H NMR (400 MHz, DMSO-D_6_) δ 1.20 (t, 3H), 2.82 (td, 2H), 2.94 (dd, 2H), 3.27 (s, 1H), 4.11 (m, 2H), 4.23 (m, 1H), 5.00 (m, 2H), 5.77 (dd, 1H), 6.86 (dd, 1H), 6.96 (ddd, 1H), 7.05 (m, 2H), 7.27 (m, 8H), 7.57 (d, 1H), 7.64 (d, 1H), 8.24 (m, 1H), 8.41­(m, 2H), 10.76 (s, 1H).

##### Ethyl (*S*,*E*)-4-((*S*)-2-(4-Methoxy-1*H*-indole-2-carboxamido)-3-phenylpropanamido)-5-(oxazol-5-yl)­pent-2-enoate (**VK-224**)

(Yield 10 mg, 85%). (LC-MS: *t*_R_ = 4.96 min; C_29_H_30_N_4_O_6_, *m*/*z*: calcd 530.2; found 531.3 (M + H)). ^1^H NMR (400 MHz, DMSO-D_6_) δ 1.20 (td, 3H), 2.98 (m, 4H), 3.27 (s, 1H), 3.89 (d, 3H), 4.13 (m, 2H), 4.73 (dq, 2H), 5.82 (dd, 1H), 6.50 (dd, 1H), 6.88 (m, 2H), 6.98 (dd, 1H), 7.11 (m, 2H), 7.28 (m, 5H), 8.20 (d, 1H), 8.40 (t, 1H), 8.52 (dd, 1H), 11.47 (dd, 1H).

##### Ethyl (*S*,*E*)-4-((*S*)-2-(4-Methoxy-1*H*-indole-2-carboxamido)-4-methylpentanamido)-5-(2-oxo-1,2-dihydropyridin-3-yl) pent-2-enoate (**VK-225**)

(Yield 12 mg, 75%). (LC-MS: *t*_R_ = 4.58 min; C_28_H_34_N_4_O_6_, *m*/*z*: calcd 522.3; found 523.4 (M + H)). ^1^H NMR (400 MHz, DMSO-D_6_) δ 0.87 (ddd, 6H), 1.18 (td, 3H), 1.56 (m, 2H), 2.69 (dd, 1H), 2.78 (td, 1H), 3.26 (s, 2H), 3.89 (d, 3H), 4.10 (pd, 2H), 4.44 (td, 1H), 4.72 (d, 1H), 5.78 (dd, 1H), 6.04 (dt, 1H), 6.50 (m, 1H), 6.84 (ddd, 1H), 7.01 (dd, 1H), 7.09 (td, 1H), 7.28 (m, 3H), 8.22 (dd, 1H), 8.30 (m, 1H), 11.52 (d, 2H).

##### Ethyl (*S*,*E*)-5-(2-Carbamoylphenyl)-4-((*S*)-2-(4-methoxy-1*H*-indole-2-carboxamido)-4-methylpentanamido) pent-2-enoate (**VK-291**)

(Yield 5 mg, 10%). (LC-MS: *t*_R_ = 5.17 min; C_30_H_36_N_4_O_6_, *m*/*z*: calcd 548.3; found 548.3 (M + H)). ^1^H NMR (400 MHz, DMSO-D_6_) δ 0.75 (m, 6H), 0.96 (m, 1H), 1.14 (m, 4H), 1.31 (m, 1H), 2.71 (dd, 1H), 3.06 (td, 1H), 3.81 (s, 3H), 4.04 (qd, 2H), 4.25 (s, 1H), 4.57 (s, 1H), 5.85 (dd, 1H), 6.44 (dd, 1H), 6.81 (m, 1H), 6.94 (d, 1H), 7.03 (m, 4H), 7.23 (m, 1H), 7.34 (dt, 1H), 7.45 (d, 1H), 7.55 (s, 1H), 7.86 (s, 1H), 8.17 (t, 1H), 8.61 (d, 1H), 11.43 (d, 1H).

#### Synthesis of Vinyl Sulfones (**VK-192** and **VK-261**)

##### 4-Methyl-*N*-((*S*)-1-oxo-3-phenyl-1-(((*S*,*E*)-4-(phenylsulfonyl)-1-(pyridin-3-yl)­but-3-en-2-yl)­amino)­propan-2-yl)­piperazine-1-carboxamide (**VK-261**)

In a 100 mL round-bottom@@ flask, diethyl ((phenylsulfonyl)­methyl) phosphonate (0.12 mmol, 1.2 equiv, 35 mg) is dissolved in 10 mL of dry THF under N_2_ and cooled to −78 °C in dry ice. To this solution, LHMDS (0.15 mmol, 1.5 equiv, 30 μL) is added and the solution is stirred for 10–15 min. **S2u** (0.1 mmol, 1 equiv, 45 mg) is dissolved in 2–5 mL of dry THF and then added to the reaction mixture, and the reaction is stirred for 30–40 min. After reaction completion, LHDMS is quenched with 1–2 mL acetic acid. THF was removed under vacuum, and the solution was basified with saturated Na_2_CO_3_ solution. The compound was extracted twice with EtOAc (2 × 25 mL), and the organic layer was washed with brine (2 × 50 mL) and finally dried over anhydrous Na_2_SO_4_. The compound (**VK-261**) was concentrated under vacuum, dissolved in 3 mL of DMF and purified using prep-HPLC using a linear gradient of (5–40% acetonitrile in water (v/v)) with 0.1% formic acid (v/v) in the mobile phase. Lyophilization resulted in a white powder: **VK-261** (yield: 26 mg, 50%). (LC-MS: *t*_R_ = 2.75 min; C_30_H_35_N_5_O_4_S, *m*/*z*: calcd 561.2; found 562.2 (M + H)). ^1^H NMR (400 MHz, DMSO-D_6_) δ: 2.16 (s, 3H), 2.20 (m, 5H), 2.46 (d, 1H), 2.68 (m, 2H), 3.04 (dd, 1H), 3.19 (m, 4H), 4.12 (ddd, 1H), 4.75 (td, 1H), 6.53 (d, 1H), 6.95 (m, 2H), 7.15 (m, 3H), 7.24 (m, 3H), 7.63 (m, 3H), 7.73 (m, 1H), 7.83 (m, 2H), 8.39 (m, 3H). The singlet peak at δ 8.15 (s,1H) corresponds to the presence of 1 equiv of formic acid.

##### 4-Methoxy-*N*-((*S*)-4-methyl-1-oxo-1-(((*S*,*E*)-4-(phenylsulfonyl)-1-(pyridin-3-yl)­but-3-en-2-yl)­amino)­pentan-2-yl)-1*H*-indole-2-carboxamide (**VK-192**)

(yield: 40 mg, 70%). (LC-MS: *t*_R_ = 4.28 min; C_31_H_34_N_4_O_5_S, *m*/*z*: calcd 574.2; found 575.2 (M + H)). ^1^H NMR (400 MHz, DMSO-D_6_) δ: 0.83 (m, 6H), 1.20 (m, 1H), 1.48 (s, 1H), 1.50 (m, 2H), 2.28 (m, 2H), 2.92 (m, 1H), 3.26 (s, 2H), 3.37 (td, 2H), 3.62 (q, 1H), 3.89 (d, 2H), 4.01 (qd, 1H), 4.41 (m, 1H), 5.09 (t, 1H), 6.52 (d, 1H), 6.76 (dt, 1H), 7.09 (m, 3H), 7.34 (m, 1H), 7.63 (m, 6H), 7.84 (m, 1H), 7.88 (m, 1H), 8.32 (m, 1H), 8.40 (m, 1H), 9.01 (s, 1H), 11.55 (d, 1H).

#### Synthesis of **BC-803**

##### *N*-((*S*)-1-(((*S*)-1-Cyano-2-(2-oxo-1,2-dihydropyridin-3-yl)­ethyl)­amino)-4-methyl-1-oxopentan-2-yl)-4-methoxy-1*H*-indole-2-carboxamide (**BC-803**)

To a suspension of SM-1 (232 mg, 0.497 mmol) in anhydrous DCM (15 mL) at room temperature was added Burgess reagent (237 mg, 0.993 mmol, 2 equiv) at once and stirred at RT for 7 h under N_2_ atmosphere. After completion of the reaction, confirmed by LC-MS, it was quenched with 1 mL water and DCM was evaporated. The product **BC-803** was purified using Silica gel Column Chromatography using 0.5–7% MeOH/DCM and further purified by Preparative HPLC using 55–75% ACN/H_2_O. (Yield 76 mg, 34%). (LC-MS: *t*_R_ = 2.75 min; C_24_H_27_N_5_O_4_, *m*/*z*: calcd 449.2; found 550.1 (M + H)). ^1^H NMR (400 MHz, DMSO-D_6_) δ 0.81 (ddd, 6H), 1.40 (m, 1H), 1.57 (m, 2H), 2.79 (m, 1H), 2.89 (dd, 1H), 3.82 (s, 3H), 4.39 (m, 1H), 5.00 (qd, 1H), 6.07 (td, 1H), 6.44 (d, 1H), 6.95 (d, 1H), 7.03 (t, 1H), 7.26 (m, 2H), 7.34 (td, 1H), 8.36 (dd, 1H), 8.86 (t, 1H), 11.48 (d, 1H), 11.65 (s, 1H).

### Cathepsins L and B

Recombinantly produced human cathepsins L and B were obtained from Millipore Sigma or R&D Systems Inc. and used without further purification. Proteins were aliquoted or diluted into 1× Cathepsin buffer (50 mM sodium acetate (pH 5.5), 1 mM Na_2_EDTA, 1 mM CHAPS, and 5 mM DTT) to final protein concentrations of 100–1000 nM aliquots and stored at 4 °C until use. These protein samples were then diluted into the same buffer to concentrations of ∼2 nM for hCatL and ∼100 nM for hCatB, and these dilutions were stored at 4 °C and used daily until depletion. Finally, these proteins were diluted 20-fold for kinetic assays, such that final concentrations used were ∼0.1 nM for hCatL and ∼5 nM for hCatB.

### Expression and Purification of SARS-CoV-2 3CL protease (3CL-PR)

The plasmid vector used to design the construct for 3CL-PR is pE-SUMO.^39^ The plasmid encodes a reading frame that expresses a protein with the N-terminal His-SUMO tag; the SUMO moiety is recognized by the *Saccharomyces cerevisiae* Ulp1 protease, and the cleavage site recognized by this protease is G*SGF-3CL. This construct was transformed into *Escherichia coli* BL21 - DE3 cells for protein expression. A 25 mL starter culture of LB media containing 100 μg/mL ampicillin was inoculated with a glycerol stock of *E*. *coli* at 23 °C overnight. The next morning, the original media was removed after centrifugation at 3000*g*. The cells were resuspended in LB media before being transferred to 1 L of LB media containing 100 μg/mL ampicillin for large-scale growth. Once OD_600_ had reached 0.6–0.8, the culture was allowed to cool to room temperature before IPTG (1 mM) was added to induce protein expression at 23 °C. Bacterial cells were centrifuged at 3000*g* and 4 °C for 30 min. EDTA-free protease inhibitor cocktail tablets were added to cell pellets suspended in 20–40 mL Lysis Buffer (50 mM sodium phosphate (pH 8.0), 300 mM NaCl, 5 mM imidazole, and 5% (v/v) glycerol, 10 mM β-ME) and were either immediately lysed by sonication or stored under −80 °C for future use. Lysates were centrifuged in an Oakridge tube at 32,000*g* for 30 min (4 °C) to remove cell debris. Each supernatant was passed through a 0.45-μm filter prior to application to a 5 mL column of nickel-NTA resin, which was then equilibrated with at least two column volumes of the Lysis Buffer, followed by washings with 2 column volumes (CVs) of 90:10 Lysis Buffer: Elution Buffer (50 mM sodium phosphate (pH 8.0), 300 mM NaCl, 500 mM imidazole, 5% (v/v) glycerol, and 10 mM β-mercaptoethanol). Elution of resin-bound protein was achieved by a stepwise increase to 40% of the Elution buffer (60:40 Lysis Buffer: Elution Buffer), as indicated by absorbance at 280 nm in the eluted fractions. 3CL-PR was dialyzed overnight in Dialysis Buffer (50 mM sodium phosphate (pH 8.0), 300 mM NaCl, 5% (v/v) glycerol, and 10 mM β-mercatoethanol) at 4 °C. The cleavage of the N-terminal His-SUMO tag was initiated by adding 0.5 mg of His_6_-Ulp1 protease into the pooled eluates comprising the peak at A_280_ nm and corresponding bands observed on SDS-PAGE. The cleaved His-SUMO tag, Ulp1, and any other impurities from the 3CL-PR were trapped in a second HisTrap Ni column. After Ulp1 protease-catalyzed cleavage, the activity of 3CL-PR was confirmed with an activity assay. The concentration of the flow-through from the second column containing pure SARS-CoV-2 3CL-PR was determined by recording the A_280_ on a NanoDrop device and calculated with a reliable extinction coefficient (32,890 M^–1^ cm^–1^), followed by storage in Dialysis Buffer at 4 °C. The typical yield for this construct is around 50 mg protein per liter of bacterial culture.

### Kinetic Assays of Inhibition of Human Cathepsins L and B

Human cathepsin L and cathepsin B assays were conducted using Cbz-Leu-Arg-AMC (Z-LR-AMC) as the substrate. 1 mM solutions of Cbz-Leu-Arg-AMC of substrate were prepared in 100% DMSO, from which dilutions to 10 μM final substrate concentration for hCatL assays and 50 μM final substrate concentration for hCatB were used in the assays performed. Both cathepsins shared the same buffer components and reaction conditions. Reaction buffer consisted of 50 mM sodium acetate (pH = 5.5), 1 mM CHAPS, 1 mM disodium EDTA, and 5 mM DTT at 25 °C. During each study, variable concentrations of inhibitor were added to the reaction buffer containing substrate to a final volume of 250 μL such that the concentration of DMSO in the reaction mixture remained fixed at 10% (v/v). Reactions were initiated by the addition of the protease to a final concentration of 0.1 nM for hCatL and 5 nM for hCatB. Michaelis–Menten constants for Z-LR-AMC were determined for hCatL (*K*_m_ = 4.4 ± 0.4 μM) and for hCatB (*K*_m_ = 25 ± 2 μM), and fixed concentrations of 2× *K*_m_ of substrate (10 μM for hCatL and 50 μM for hCatB) were used to evaluate inhibitors. In a 96-well, clear black-bottom (Greiner) plate, time courses of fluorescence that correspond to the formation of AMC product were measured for 0–30 min at 6-s intervals. Peptidolysis of the AMC substrate was measured in a Synergy MX microplate reader (Biotek, Winooski, VT) using an excitation wavelength of λ_ex_ = 360 nm with detection of emission at λ_em_ = 460 nm. The measured relative fluorescence units (RFUs) were converted into reaction rates of μM s^–1^ by using a standard curve of known concentrations of the product AMC.

### Kinetic Assays of Inhibition of SARS-CoV-2 3CL-PR

3CL-PR assays were conducted in 100-μL reaction mixtures containing final concentrations of 20 mM Tris-HCl (pH = 7.5), 150 mM NaCl, 0.1 mM EDTA, 2 mM DTT, and a 50 μM final concentration of the FRET-based substrate Dabcyl-Lys-Thr-Ser-Ala-Val-Leu-Gln*Ser-Gly-Phe-Arg-Lys-Met-Glu-Edans (Dabcyl-KTSAVLQSGFRKME-Edans), added from a 10 mM stock solution prepared in 100% DMSO. Variable concentrations of inhibitors were added from stock solutions prepared in 100% (v/v) DMSO, so that the final concentration of DMSO in all samples was 10% (v/v).

Reaction was initiated by the addition of 3CL-PR to a final concentration of 5 nM. Kinetic parameters for this substrate were ascertained: (*K*_m_ = 28.0 ± 3.7 μM). Time courses of fluorescence were acquired for 30 min in 8 s intervals in a 96-well plate (clear-bottom, half-volume black-bottom, Greiner) using a Synergy HTX (Biotek, Winooski, VT) microplate reader. The excitation and emission wavelengths for this FRET substrate were λ_ex_ = 342 nm and λ_em_ = 520 nm, respectively. The measured relative fluorescence units (RFUs) were converted into reaction rates of μM s^–1^ by using a standard curve of known concentration of fully hydrolyzed substrate.

### Molecular Docking

Covalent docking was performed on Schrodinger Release 2023-1, Maestro version 13.5.132 (Schrodinger LLC, NY) of selected compounds with human Cathepsin L (PDB: 8QKB, 1.60 Å).^36,37^ The 8QKB crystal structure was bound with a vinyl sulfone inhibitor, **K11777**, and prepared through the protein preparation wizard tool for docking studies.^36^ The preparation of hCatL consisted of removing all water molecules, removing the crystallization solvents, and changing the protonation state of residues to be consistent with a pH of 5.5. **K11777** was used as the center region of docking studies with default settings, with Cys_25_ identified as the receptor in chemistry. Ligands were constructed using ChemDraw 22.2.0 (Revvity Signals Software Inc., MA) and prepared using the ligand preparation wizard. Ligands were energetically minimized and protonated at pH 5.5 ± 2. Covalent docking was carried out using the default docking settings of CovDoc and the built-in chemistry reaction for Michael addition to a Cys residue in the Thorough pose prediction docking mode. Generated poses were evaluated by the docking score parameter and exported as PDB files for visualization in ChimeraX v1.9 (UCSF, CA). LigPlot+ version 2.2.9 (EMBL-EBI, UK) was used to plot ligand-residue interactions. The bond parameters used were as follows: hydrogen-bond: 2.70 Å maximum H-acceptor distance, 3.35 Å maximum donor–acceptor distance. Nonbonded/hydrophobic contact: 2.90 Å minimum contact distance, 3.90 Å maximum contact distance.

The 8HE9 crystal structure was bound with a vinyl sulfone inhibitor, **K11777**, and prepared through the protein preparation wizard tool for docking studies.^36^ The preparation was carried out for both proteins, as mentioned above. **K11777** was used as the center of the Glide receptor grid for receptor grid generation, under standard settings, for both 8QKB and 8HE9. Ligands were constructed using ChemDraw 22.2.0 (Revvity Signals Software Inc., MA) and prepared using the ligand preparation wizard. Ligands were energetically minimized and protonated at pH 5.5 ± 2. Ligand docking was carried out using the default docking settings of LigDoc in standard pose prediction docking mode.

### Ethics Statement

All experiments involving animal and infectious virus were carried out at the Galveston National Laboratory (GNL) at The University of Texas Medical Branch at Galveston (UTMB), Texas, an Associated for Assessment and accreditation of Laboratory Animal Care International (AAALAC) accredited (November 24, 2020) and the Office of Laboratory Animal Welfare (OLAW) approved (February 26, 2021) high-containment National Laboratory, based on a protocol reviewed and approved by the Institutional Animal Care and Use Committee (IACUC) at UTMB Galveston (Protocol Number: 2205027).

### *In Vitro* Evaluation of Efficacy in SARS-CoV-2 Infection

Vero E6 cells [CRL:1586, ATCC], derived from African green monkey cells and human A549 cells stably transduced with the human ACE2 viral receptor (A549/ACE2) and selected for high ACE2 expression, were grown in Eagle’s minimal essential medium (EMEM) supplemented with 10% fetal bovine serum (FBS), and 100 U/mL penicillin and 100 μg/mL streptomycin (M-10 medium). SARS-CoV-2 (USA_WA1/2020 isolate), provided through the World Reference Center for Emerging Viruses and Arboviruses (WRCEVA), was used throughout the study. A modified cell-based standard microneutralization assay was used to rapidly evaluate the drug efficacy against SARS-CoV-2 infection. Briefly, confluent Vero E6 or A549/ACE2 cells grown in 96-well microtiter plates were pretreated with 78 nM–20 μM of the protease inhibitors (2-fold serially diluted) for 2 h, before infection with ∼100 or ∼500 infectious SARS-CoV-2 particles, respectively, in 100 μL of EMEM supplemented with 2% FBS (2-MEM). Samples contained 2 μM of the MDR inhibitor CP-100356, which was used for the Vero E6 assay.^13,17,23^ Cells pretreated with Vehicle (DMSO or DMSO/CP-100356), with or without virus, were included as positive and negative controls, respectively. After incubation at 37 °C for 3 days (Vero E6 cells) or 4 days (A549/ACE2 cells), individual wells were examined by microscopy to assess the virus-induced cytopathic effect (CPE) formation. The efficacy of individual drugs was calculated and expressed as the lowest concentration capable of completely preventing virus-induced CPE in 100% of the wells. The values of EC_50_ (the concentration of inhibitor that results in 50% growth of virus) were determined in two ways based on the presentation of the two replicates. If duplicate samples of a single compound dilution both displayed 100% CPE for both replicates and in which no CPE was observed at the next highest concentration of the inhibitor in duplicates, the assigned EC_50_ was the average of these two concentrations. In the event that duplicate concentrations result in one sample displaying CPE while the other does not, the value of EC_50_ was assigned to this concentration.

### *In Vivo* Analysis of an Antiviral Inhibitor in a Murine Model of SARS-CoV-2 Infection

Two groups of five Balb/c mice (15–16 weeks old, The Jackson Laboratory) were anaesthetized with isoflurane in oxygen and challenged intranasally (IN) with 10^5^ 50% tissue culture infectious dose (TCID_50_) of mouse-adapted SARS-CoV-2 (MA-10) diluted in 60 μL of MEM supplemented with 2% FBS, 2 h after intraperitoneal (IP) administration of 0.2 mL of vehicle DMSO:PEG400:PBS (v/v/v; 4.7%:47.7%:47.7%), or vehicle containing 40 mg/kg **VK-80** + 20 mg/kg ritonavir (RTV). An additional group of 5 mock-infected mice received identical doses of 40 mg/kg of **VK-80** + 20 mg/kg of RTV to test the tolerability to VK-80. Dosing was continued at 4 h postinfection on Day 0 and then BID on Days 1–4. Animals were monitored at least once daily for weight changes, other signs of illness, and mortality based on a 1–4 grading system (1: Healthy, 2: ruffled fur, lethargic, 3: hunched posture, orbital tightening, increased respiratory rate, >15% weight loss, and 4: dyspnea, cyanosis, reluctance to move when stimulated, >20% weight loss).

### Theory of Enzyme Inhibition vs Inactivation and Data Analysis

Scheme 9 depicts an inhibitor or inactivator **I**, which is competitive with substrate **A** in terms of binding to the free enzyme form **E**, along with the germane microscopic rate constants. The Michaelis complex **EA** and the initial inhibition complex **EI** are established rapidly (msec to sec, depending on the concentrations of the reagents) after the addition of enzyme **E** to a reaction mixture containing both **A** and **I**. In this scheme, *k*_cat_ is the enzymatic turnover number and *K*_m_ = (*k*_off_ + *k*_cat_)/*k*_on_. We define inhibition as the case in which the EI* complex may return to the free enzyme **E**. Inactivation occurs when the EI* complex does not reverse (when *k*_4_ = 0), regardless of whether covalent adduction has occurred between **E** and **I** as depicted in the *k*_5_ step. Time-dependent inhibition, whether reversible or irreversible, is observed when the **EI** complex isomerizes to a tight-binding complex EI* over seconds or longer and in which *k*_3_ ≫ *k*_4_. In Scheme 9, we consider a case in which the **EI** complex isomerizes to the EI* complex prior to a chemical step (*k*_5_) that results in a covalent adduct. We use the method of net rate constants^41^ to derive general expressions for enzyme inhibition and inactivation for this scheme. Covalent inactivation of the enzyme is described by the *k*_5_ step, but may be the *k*_3_ step when *k*_4_ = 0. In Scheme 9, net rate constants are *k*_5_′ = *k*_5_, *k*_3_′ = *k*_3_ *k*_5_/(*k*_4_ + *k*_5_), and *k*_1_′ = *k*_1_ *k*_3_′[I] = *k*_1_ *k*_3_ *k*_5_[I]/[*k*_2_(*k*_4_ + *k*_5_) + *k*_3_ *k*_5_]. Expressions for the rates of inactivation when [**I**] is very low or very high are given by eqs 1 and 2, respectively:

1 $$k_{\mathrm{inact}}/K_{I}=k_{1\prime }{\left[E\right]}_{t}/\left[I\right]=k_{1}k_{3}k_{5}{\left[E\right]}_{t}/\left[k_{2}\left(k_{4}+k_{5}\right)+k_{3}k_{5})\right]=K_{\mathrm{eq}3}k_{5}/(K_{i}\left[(1+\left(k_{5}/k_{4}\right)\left(1+k_{3}/k_{2}\right)\right]$$

2 $$k_{\mathrm{inact}}=k_{3}k_{5}{\left[E\right]}_{t}/\left(k_{3}+k_{4}+k_{5}\right)$$

3 $$K_{i}=k_{2}/k_{1}$$

4 $$K_{I}=K_{i}\left[k_{4}+k_{5}\left(1+k_{3}/k_{2}\right)\right]/\left(k_{3}+k_{4}+k_{5}\right)$$

When *k*_5_ = 0, *K*_I_ = *K*_i_ *k*_4_/(*k*_3_ + *k*_4_) which is the same as the tight-binding inhibition constant *K*_I_*.^42^

**Scheme 9:**
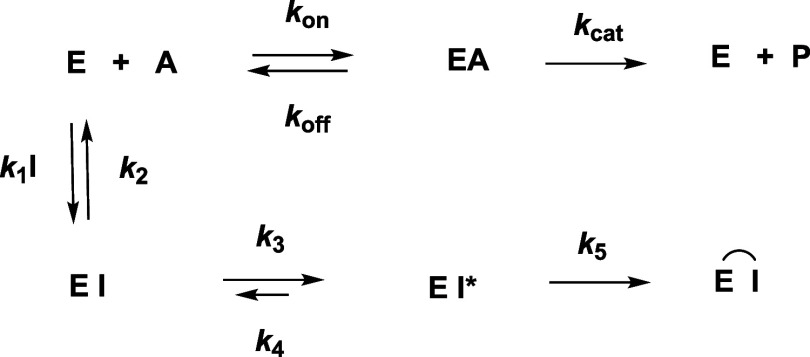


**Case 1**. **Reversible tight-binding inhibition**. When *k*_4_ > 0 and *k*_5_ = 0, the **EI** and EI* complexes achieve equilibrium over time. Time courses of product formation vs time at multiple concentrations of inhibitor present as a family of downward curvilinear lines that have decreased slopes at longer values of time. These plots may be characterized by the two inhibition constants: *K*_i_ = *k*_2_/*k*_1_ and *K*_i_* = (*k*_2_/*k*_1_)­(*k*_4_/(*k*_3_ + *k*_4_)).^42^ Kinetic evaluation of time-dependent kinetics gives true values for *K*_i_ and *K*_i_*.

**Case 2**. **Irreversible**, **noncovalent tight-binding inactivation**. When *k*_4_ = *k*_5_ = 0,^25^ the formation of the EI* complex is kinetically irreversible, and product vs time data are also downward curvilinear lines, whose slopes appear to be zero at higher values of time, symptomatic of enzyme inactivation. Here, measurable kinetic parameters become *k*_inact_ = *k*_3_ and *K*_I_ = (*k*_2_ + *k*_inact_)/*k*_1_, in which *k*_inact_ and *K*_I_ are similar to the catalytic parameters *k*_cat_ and the Michaelis constant *K*_m_ for substrate. Here, apparent inactivation may or may not involve the formation of a covalent adduct.

**Case 3**. **Irreversible**, **covalent inactivation**. When *k*_4_ and *k*_5_ ≥ 0,^22^ the formation of the EI* complex is ultimately kinetically irreversible, and product vs time data are also composed of downward curvilinear lines, whose slopes tend to zero at higher values of time, as in Case 2. While *k*_4_ may be greater than *k*_5_, over time, all of the enzymes will exist in a covalent complex.

In order to differentiate between the three cases above, we evaluate the kinetics of our compounds with three different methods. (1) Time courses of fluorescence data were acquired over 30 min, from which we ascertained initial velocities (***v***_**i**_) (*t* = 0–180 s) and steady-state velocities (***v***_**s**_) (*t* = 1600–1800 s) in the presence and absence of inhibitors, and ***v***_**i**_ and ***v***_**s**_ were used to determine the apparent inhibition constants *K*_i_ and *K*_i_*, respectively. Time courses were measured at a single concentration of substrate and variable concentrations of inhibitor, in which the reaction is initiated by the addition of enzyme [**E**_**t**_] ≤ [**I**]. As noted above, in cases in which covalent activation occurs over time, ***k***_**5**_ > 0, and *K*_i_* is no longer a true equilibrium constant. These data were evaluated as follows: Plots of ***v***_**i**_ **/** ***v***_**0**_ and ***v***_**s**_ **/** ***v***_**0**_ vs [inhibitor] were fitted to eqs 5 and 6, wherein ***v***_**i**_ and ***v***_**s**_ are velocities at, respectively, early and late stages of the time courses of inhibition as described above, ***v***_**0**_ is ***v***_**i**_ and ***v***_**s**_ when no inhibitor is present, [I] is variable concentrations of a competitive inhibitor, and *K*_m_ is the Michaelis constant of the substrate A.

5 $$\frac{v_{i}}{v_{0}}=\frac{1}{\left\{1+\frac{\left[I\right]}{\left[K_{i}\left(1+\frac{\left[A\right]}{K_{m}}\right)\right]}\right\}}$$

6 $$\frac{v_{s}}{v_{0}}=\frac{1}{\left\{1+\frac{\left[I\right]}{\left[K_{i}^{*}\left(1+\frac{\left[A\right]}{K_{m}}\right)\right]}\right\}}$$

7 $$\frac{v_{x}}{v_{0}}=1-\left\{{[E}_{t}]+\left[I\right]+K_{i}\times \left(1+\frac{\left[A\right]}{K_{m}}\right)-\sqrt{{\left\{\left[E_{t}\right]+\left[I\right]+K_{i}\times \left(1+\frac{\left[A\right]}{K_{m}}\right)\right\}}^{2}-4\times \left[E_{t}\right]\times \left[I\right]}\right\}/\left\{2\times \left[E_{t}\right]\right\}$$

Inhibitors for which *K*_i_* and/or *K*_i_ ∼ [E_t_] and [I] were fitted to eq 7, in which ***v***_**x**_ is the velocity of the enzymatic reaction at different inhibitor concentrations, ***v***_**0**_ is the velocity of enzymatic reaction in the absence of inhibitor, E_t_ is the concentration of enzyme in the assay, [I] is the inhibitor concentration, *K*_i_ is the inhibition constant when ***v***_**x**_ is ***v***_**0**_, and *K*_i_* is the inhibition constant when ***v***_**x**_ is ***v***_**s**_, [A] is the substrate concentration used in the assay and *K*_m_ is the Michaelis–Menten constant of the substrates as detailed above. Equation 7 reduces to eqs 5 and 6 when [E_t_] ≪ [I].

Time-course data for compounds that exhibited time-dependent inhibition were also fitted to eq 8, for which *P* is the concentration of the peptidolytic product, ***v***_**i**_ and ***v***_**s**_ are as described above, *k*_obs_ is the rate constant of conversion of ***v***_**i**_ to ***v***_**s**_, *t* is time in seconds, and *C* is a background constant.

8 $$P=v_{s}t+\left(\frac{v_{i}-v_{s}}{k_{\mathrm{obs}}}\right)\left(1-e^{-k_{\mathrm{obs}}t}\right)+C$$

For Scheme 9, *k*_obs_ = *k*_3_′[I] + *k*_4_ = *k*_inact_[I] + *k*_4_ for which *k*_3_′ is mechanism-dependent. Resulting values of *k*_obs_ vs [I] were replotted and fitted to eq 9, in which *k*_inact_ (*k*_3_′) and *k*_4_ are described in Scheme 9, for which A is the fixed substrate concentration and *K*_m_ is the Michaelis constant.

9 $$k_{\mathrm{obs}}=k_{4}+\frac{k_{\mathrm{inact}}\left[I\right]}{K_{I}\left(1+\frac{\left[A\right]}{K_{m}}\right)+\left[I\right]}$$

Linearity of *k*_obs_ vs [I] of inhibitor compounds will be observed when *K*_i_ ≫ *K*_i_*. Under these conditions, the concentration of inhibitor required to observe time-dependent inhibition would be much lower than the value of *K*_i_ for the **EI** complex. In this case, eq 9 reduces to eq 10, which is a linear function with a slope = *k*_inact_/*K*_I_ and a *y*-intercept = *k*_4_.

10 $$k_{\mathrm{obs}}=k_{4}+\frac{k_{\mathrm{inact}}\left[I\right]}{K_{I}\left(1+\frac{\left[A\right]}{K_{m}}\right)}$$

A useful study to ascertain the value of *k*_4_ is that of “jump dilution” in which inhibitors are preincubated with enzyme prior to a 100-fold dilution into reaction mixtures containing high concentrations of substrate. When inhibition is reversible (*k*_4_ > 0), time courses of [P] vs time appear as upward curvilinear traces, characterized by an absent initial rate (***v***_**i**_), followed by recovery of enzyme activity (***v***_**s**_) upon dissociation of inhibitor over time. Data from these plots were fitted to eq 11, in which *k*_obs_ is equal to *k*_4_ in Scheme 9.

11 $$P=v_{s}t+\left(\frac{-v_{s}}{k_{\mathrm{obs}}}\right)\left(1-e^{-k_{\mathrm{obs}}t}\right)+C$$

Throughout this paper, we refer to kinetic data as enzyme inhibition until sufficient data is acquired to indicate that kinetic inactivation is present.

### Native Mass Spectrometry

Proteins for native mass spectrometry (MS) study were prepared as previously reported.^44^ In brief, 20 μL of hCatL protein (20 μM) in 1× CatL buffer with 10% (v/v) DMSO was buffer-exchanged into 200 mM ammonium acetate at pH 7.0 with a centrifugal desalting column (Micro Bio-Spin 6, Bio-Rad). For the sample containing 2.5 × molar excess of inhibitors, **VK-80** and **VK-261** (50 μM final concentration) were added to the sample and incubated for 1 h prior to the buffer exchange. Excess inhibitors not bound to the protein were removed by using a centrifugal desalting column (Micro Bio-Spin 6, Bio-Rad). Upon buffer exchange, samples were diluted to 2 μM with 200 mM ammonium acetate (200 mM) for native MS analysis. Native MS data were collected on a Thermo Exactive Plus with an Ultra High Mass Range Orbitrap mass spectrometer (Thermo Scientific) with source DC offset at 21.0 V, Injection flatapole DC at 5.0 V, inter flatapole lens at 5.0 V, bent flatapole DC at 1.0–4.0 V, trapping gas pressure at 5.0, in-source CID at 20.0–30.0 eV, CE at 30.0–150.0 V, Desolvation voltage at −10.0 to −30.0 V, spray voltage at 1.2–1.6 kV, capillary temperature at 250 °C, scanning from 350 to 6000 *m*/*z* at resolution 12,500. Native MS data were deconvoluted using UniDec.^45^ Native MS data were collected at two different collision energies (30.0 and 150.0 V).

## Supplementary Material









## Notes

Kinetic parameters of inactivation of hCatL and hCatB; Cheng-Prusoff plots of time-dependent inhibition of CPs hCatL, hCatB, and 3CL-PR; anti-SARS-CoV-2 data in infected Vero E6 and A549/ACE2 cells; selectivity of hCatL inhibitors vs hCatB; covalent molecular docking of compounds **VK-80**, **VK-166**, **VK-168**, and **VK-172** with hCatL; analytical characterization of inhibitors (NMR, HPLC traces, and mass spectrometry); and native mass spectrometry of apo hCatL and bound hCatL showing charge states (*m*/*z*) (PDF)
Molecular formula strings (CSV)
Ligand docking of VK-80 with hCatL (PDB)
Ligand docking of VK-80 with hCatB (PDB)

## Abbreviations Used

AMC: 7-amino-4-methylcoumarin
Burgess reagent: methyl *N*-(triethylammoniumsulfonyl)­carbamate
CHAPS: 3-[(3-cholamidopropyl)­dimethylammonio]-1-propanesulfonate
DABCYL: 4-(4-dimethylaminophenylazo)­benzoic acid
DIPEA: *N*,*N*-diisopropylethylamine
DTT: dithiothreitol
EDANS: (5-((2-aminoethyl)­amino)­naphthalene-1-sulfonic acid)
HATU: hexafluorophosphate azabenzotriazole tetramethyl uranium
IPTG: isopropyl β-d-1-thiogalactopyranoside
LHMDS: lithium bis­(trimethylsilyl)­amide or lithium hexamethyldisilazide
LiAlH_4_: lithium aluminum hydride
nickel-NTA: nickel-nitrilotriacetic acid
TFA: trifluoroacetic acid
