# Supplementary material for: Peptidomimetic α,β-Unsaturated Ethyl Esters Are Irreversible Inactivators of Human Cathepsin L and Are Potent Inhibitors of SARS-CoV‑2 in Cellular Models of COVID-19
Source: J Med Chem. 2026 Apr 21;69(10):11797–818. doi: 10.1021/acs.jmedchem.5c03172 (PMC13224093; doi:10.1021/acs.jmedchem.5c03172)
Supplement: Supplementary file 1 [file jm5c03172_si_001.pdf]

SUPPLEMENTARY INFORMATION

Peptidomimetic α,β-Unsaturated Ethyl Esters are Irreversible Inactivators of Human Cathepsin L and are Potent Inhibitors of SARS-CoV-2 in Cellular Models of COVID-19

Vivek Kumar,[1,] Aleksandra Drelich[3] , Bala C. Chenna[1] Andrew Rademacher[1], Alexandria M. Kemp,[1] Anthony C. Bishop,[1] Panatda Saenkham-Huntsinger,[3] Elena Scott[2], A. Joshua Wand,[1,2] Arthur Laganowsky[2], Chien-Te Tseng[3], and Thomas D. Meek[1,2*]

[1]Department of Biochemistry and Biophysics, Texas A&M University, 301 Old Main Drive, College Station, Texas 77843, United States

[2]Department of Chemistry, Texas A&M University, 580 Ross St. College Station, TX 77843-3255 United States

[3]Department of Microbiology & Immunology Centers for Biodefense and Emerging Diseases, The University of Texas Medical Branch at Galveston, 301 University Boulevard, Galveston, Texas 77555, United States

*Email: Thomas.Meek@ag.tamu.edu

Table of Contents

Kinetic parameters of inactivation of hCatL and hCatB ……………………….S2 - S3

Cheng-Prusoff and Inactivation Kinetics…..……………………….…………..S4 - S12

Preincubation Kinetics………………………………………………………….S12 - S14

Anti-CoV-2 Data of hCatL Inhibitors…………………………………………..S15

Selectivity of hCatL Inhibitors vs. hCatB……………………………………..S16 - S17

Molecular Docking……………………………………………………………….S17 – S19

NMR, MS and HPLC Traces of Final Compounds……………………………..S20 - S45

Native Mass Spectrometry…………………………………………..………S45 - S46

Table S1. Kinetic Parameters of Ethyl Enoate and Vinyl Sulfone Inhibitors or Inactivators of Human Cathepsin L.[a]

Compound	Structure	Equation Fitted	*app* K_i (nM)	*app* K_i* (nM)	K_I (nM)	k_{inact}/K_I ($M^{-1}s^{-1}$)	k_{inact} x 10^{-2} (s^{-1})	k_4 x 10^{-4} (s^{-1})
**VK-80**	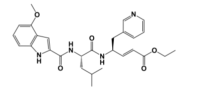	9	53 ± 9	6.4 ± 0.5	320 ± 90	(7 ± 2) x 10^4	2.1 ± 0.5	1.9 ± 0.9
**VK-303**	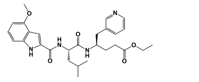	none	>25,000	ND	ND	ND	ND	ND
**VK-192**	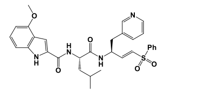	10	420 ± 40	35 ± 4	---	(2.4 ± 0.7) x 10^4	---	2.3 ± 0.6
**BC-787**	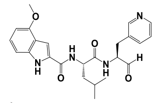	9	1.7 ± 0.2[c]	6200 ± 200[c]	1.7 ± 0.2[c]	6200 ± 200[c]	130 ± 4[c]	---
**VK-224**	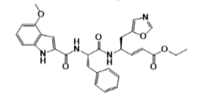	9	51 ± 6	3.8 ± 0.4	150 ± 20	(1.3 ± 0.2) x 10^5	2.0 ± 0.2	1.0 ± 0.4
**VK-225**	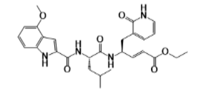	9	2000 ± 200	170 ± 20	12,000 ± 2000	(2.5 ± 0.5) x 10^3	3.0 ± 0.4	0.9 ± 0.1
**VK-291**	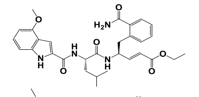	9	1100 ± 100	56 ± 5	1900 ± 300	(6.3 ± 0.5) x 10^3	1.2 ± 0.2	2.0 ± 0.4
**VK-172**	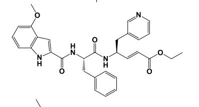	9	27 ± 5	3.2 ± 0.2	100 ± 30	(1.7 ± 0.7) x 10^5	1.7 ± 0.4	1.8 ± 0.4
**VK-187**	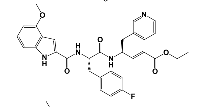	9	77 ± 6	6.2 ± 0.6	120 ± 50	(9 ± 5) x 10^4	1.1 ± 0.3	3 ± 1
**VK-188**	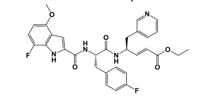	9	38 ± 3	3.1 ± 0.3	450 ± 60	(1.2 ± 0.2) x 10^5	5.6 ± 0.6	ND
**VK-166**	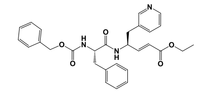	9	26 ± 2	2.6 ± 0.2	50 ± 10	(2.6 ± 0.7) x 10^5	1.2 ± 0.2	ND
**VK-178**	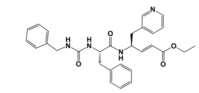	9	66 ± 7	5.0 ± 0.5	180 ± 60	(1.10 ± 0.05) x 10^4	1.9 ± 0.5	1.4 ± 0.8
**VK-223**	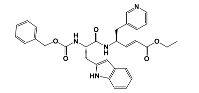	9	280 ± 20	49 ± 4	370 ± 50	(2.4 ± 0.3) × 10^4	0.88 ± 0.04	1.0 ± 0.5
**VK-168**	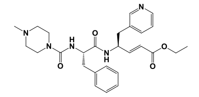	9	6.8 ± 0.6	0.58 ± 0.03	14 ± 4	(8 ± 3) × 10^5	1.1 ± 0.2	1.0 ± 0.5
**VK-261**	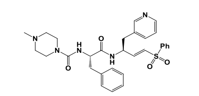	9	9 ± 2	**0.38 ± 0.06**	15 ± 8	(1.0 ± 0.2) × 10^6	1.5 ± 0.7	1.6 ± 0.7
**K11777**	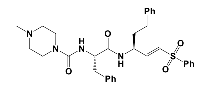	9	--	**0.15 ± 0.02**	--	(2.87 ± 0.04) × 10^6	--	--

The apparent inhibition constants (app K_i) and apparent tight-binding inhibition constants (app K_i*) were obtained at t = 0-3 min and 27-30 min, respectively. K_i* values in **bold** represent apparent steady-state inhibition constants calculated using equation 9 which includes the concentration of the enzyme since $E_t \sim I_t$. Plots of k_{obs} vs [I] plots were fitted to eqs 4 and 5, and kinetic parameters from the best fitted equation is included here. The second-order rate constants of inactivation (k_{inact}/K_I) were either from fitting to eq 4, or for fitting to eq 5, were calculated from the resulting values of k_{inact} and K_I. ND, not determinable. NF, not fitted. The average value of k_4 in the absence of added inhibitor was 1.2 ± 0.3 x 10^{-4} s^{-1}.

Table S2. Kinetic Parameters of Ethyl Enoate and Vinyl Sulfone Inactivators of Human Cathepsins B and L.[a]

Compound	Structure	hCat L 1-hr		hCat L 12-hr		hCat B 12-hr	
		k_4 x 10^{-4} (s^{-1})	v_s/E_t (s^{-1})	k_4 x 10^{-4} (s^{-1})	v_s/E_t (s^{-1})	k_4 x 10^{-4} (s^{-1})	v_s/E_t (s^{-1})
**Control (DMSO)**		NF	8.07 ± 0.02	NF	4.85 ± 0.04	NF	2.30 ± 0.01
**VK-303**	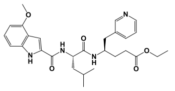	NF	7.40 ± 0.02	NF	4.93 ± 0.05	NF	2.36 ± 0.01
**VK-192**	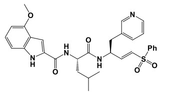	20.7 ± 0.08	0.044 ± 0.007	~0	~0	~0	2.52 ± 0.007
**BC-787**	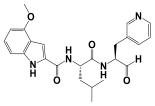	23.2 ± 0.3	2.20 ± 0.05	106 ± 1	0.291 ± 0.003	NF	1.90 ± 0.004
**VK-224**	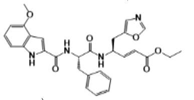	4.0 ± 0.2	0.46 ± 0.2	26.2 ± 0.02	0.0872 ± 0.002	199 ± 4	0.871 ± 0.0008
**VK-187**	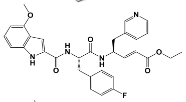	~0	2.64 ± 0.03	~0	NF	310 ± 20	2.19 ± 0.005
**VK-188**	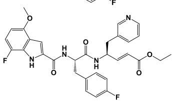	7.5 ± 0.2	0.351 ± 0.006	16.1 ± 0.4	0.084 ± 0.001	NF	2.30 ± 0.007
**VK-178**	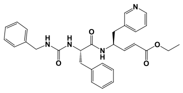	1.5 ± 0.2	1.2 ± 0.1	6.5 ± 0.2	0.187 ± 0.003	81 ± 2	0.734 ± 0.002
**VK-223**	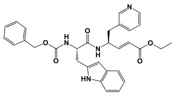	NF	6.2 ± 0.5	21.0 ± 0.2	0.287 ± 0.001	NF	2.26 ± 0.005
**VK-166**	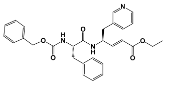	NF	NF	NF	NF	42.0 ± 0.9	0.635 ± 0.0003

[a] Data from Figures **3**, **S7** and **S8** and results of fitting to eq 11.

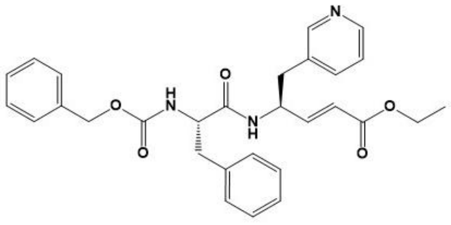

VK-166

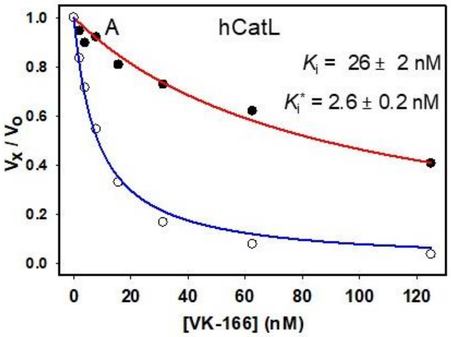

A
hCatL
K_i = 26 ± 2 nM
K_i^* = 2.6 ± 0.2 nM
v_x / v_o
[VK-166] (nM)

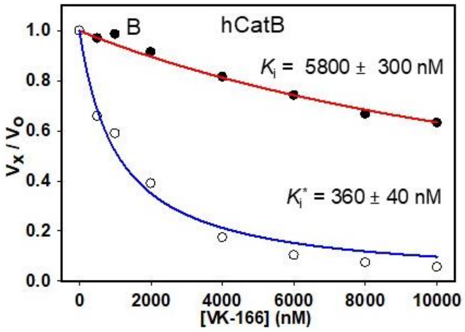

B
hCatB
K_i = 5800 ± 300 nM
K_i^* = 360 ± 40 nM
v_x / v_o
[VK-166] (nM)

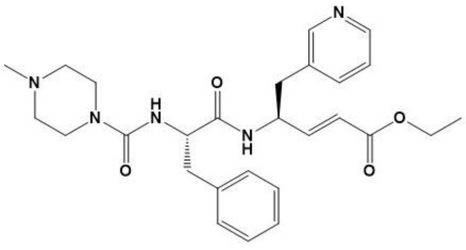

VK-168

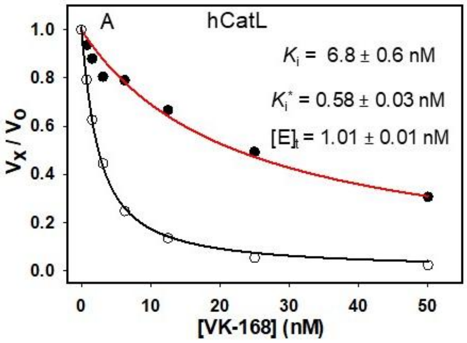

A
hCatL
K_i = 6.8 ± 0.6 nM
K_i^* = 0.58 ± 0.03 nM
$[E]_t$ = 1.01 ± 0.01 nM
v_x / v_o
[VK-168] (nM)

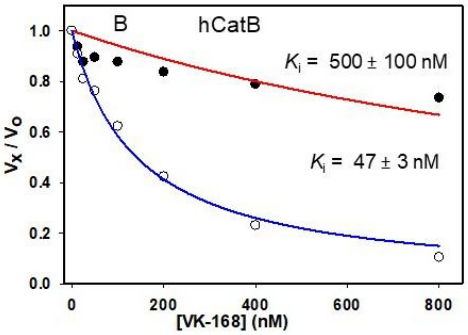

B
hCatB
K_i = 500 ± 100 nM
K_i = 47 ± 3 nM
v_x / v_o
[VK-168] (nM)

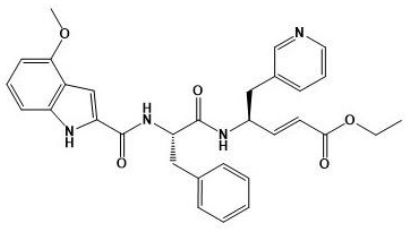

VK-172

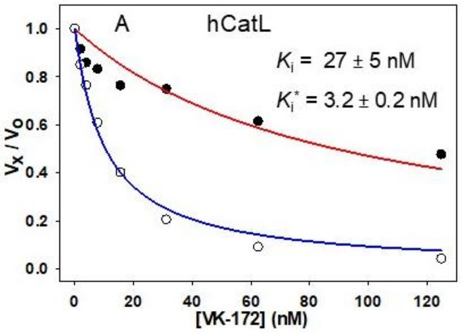

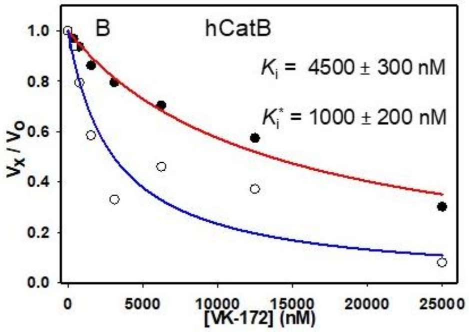

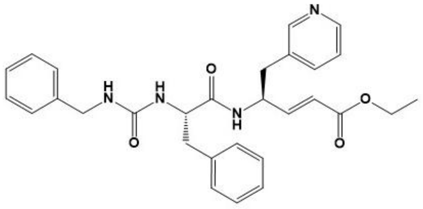

VK-178

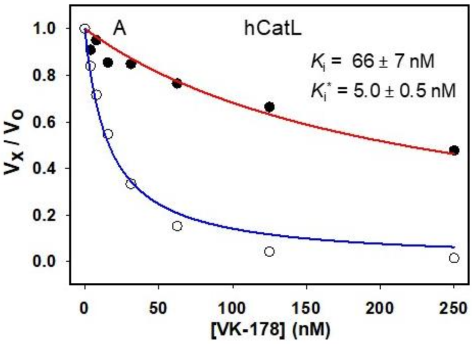

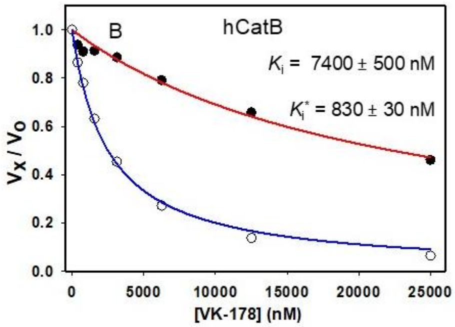

Figure S1. Compound structures and Cheng-Prusoff plots of **VK-166**, **K-168, VK-172**, and **VK-178**. Plotting of the fraction of remaining enzyme activity (v_x/v_0) vs. inhibitor concentrations at initial rates (t = 0-180 s, (v_i / v_0)) and steady-state rates (t = 1820-2000 s, (v_s / v_0)) for hCatL and hCatB. The lines drawn through the experimental data points resulted from fitting of data to eq 7 and 8, from which the inhibition constants were obtained.

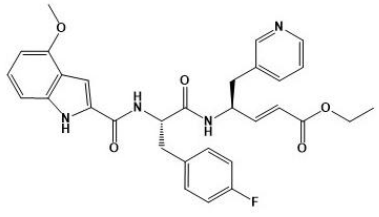

VK-187

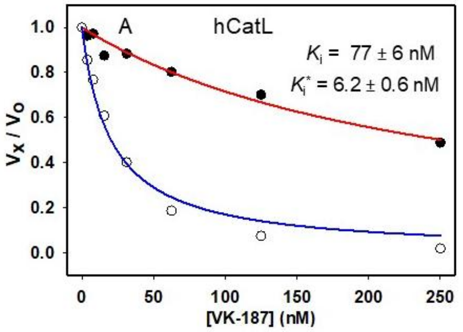
A
hCatL
K_i = 77 ± 6 nM
K_i^* = 6.2 ± 0.6 nM
V_X / V_O
[VK-187] (nM)

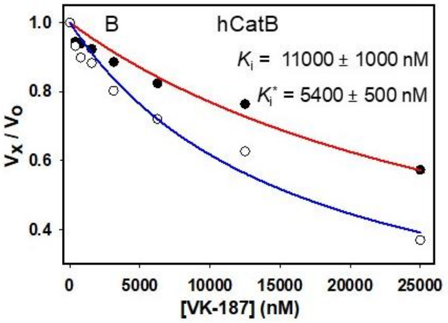
B
hCatB
K_i = 11000 ± 1000 nM
K_i^* = 5400 ± 500 nM
V_X / V_O
[VK-187] (nM)

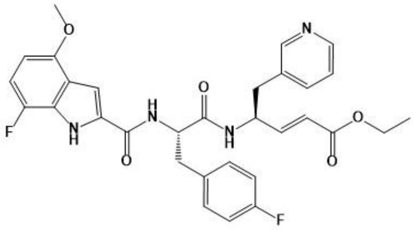

VK-188

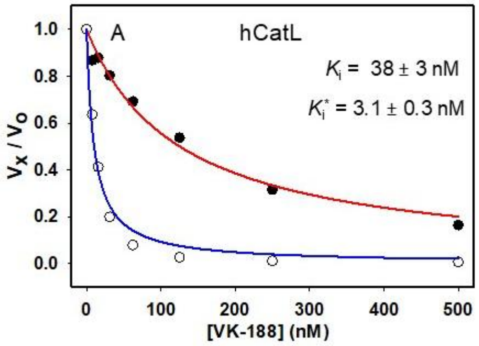
A
hCatL
K_i = 38 ± 3 nM
K_i^* = 3.1 ± 0.3 nM
V_X / V_O
[VK-188] (nM)

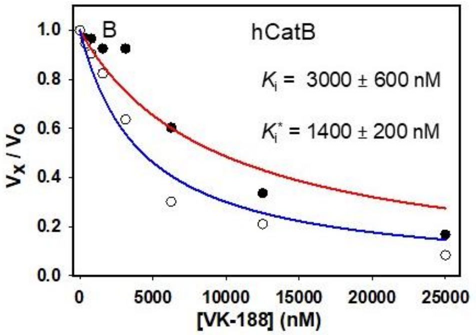
B
hCatB
K_i = 3000 ± 600 nM
K_i^* = 1400 ± 200 nM
V_X / V_O
[VK-188] (nM)

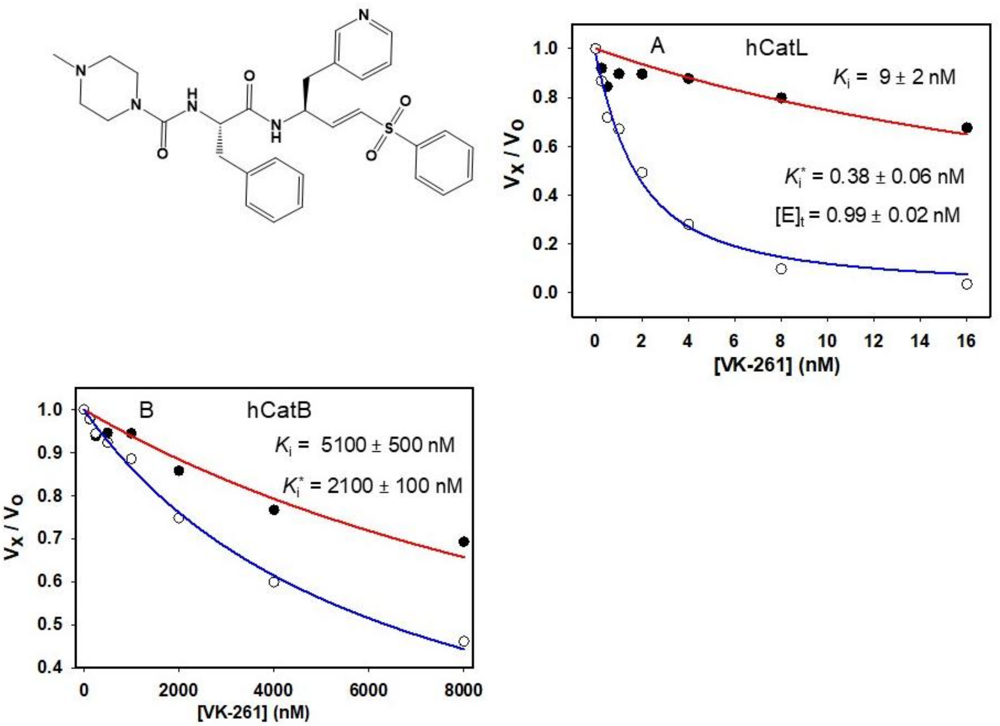

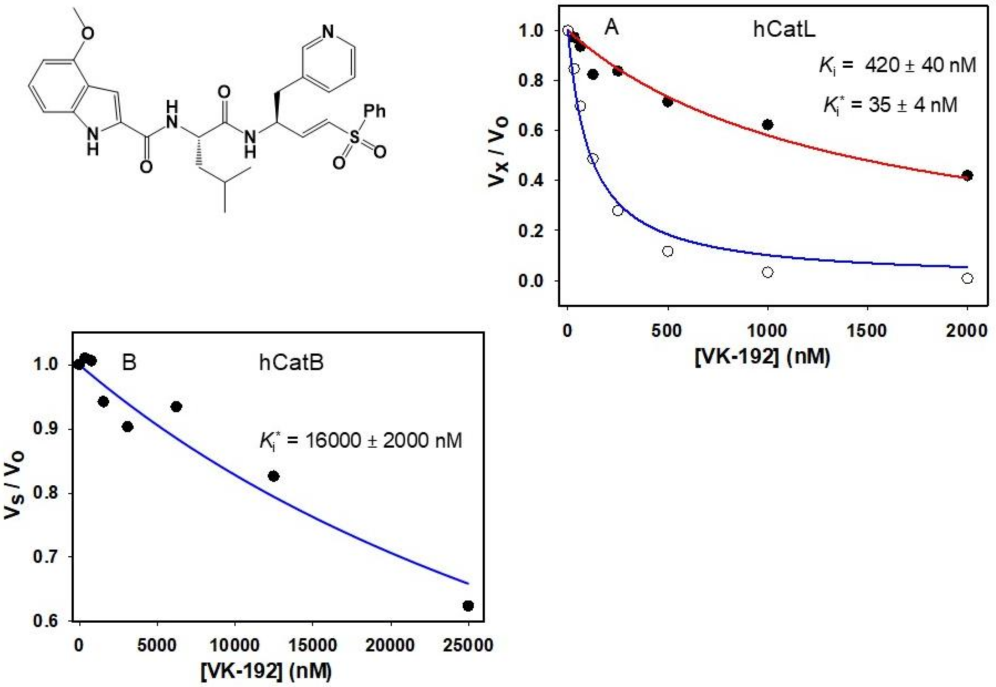

Figure S2. Compound structures and Cheng-Prusoff plots of **VK-187**, **K-188, VK-261**, and **VK-192**. Plotting of the fraction of remaining enzyme activity (v_x/v_0) vs. inhibitor concentrations at initial rates (t = 0-180 s, (v_i / v_0)) and steady-state rates (t = 1820-2000 s, (v_s / v_0)) for hCatL and hCatB**.** The lines drawn through the

experimental data points resulted from fitting of data to eq 7 and 8, from which the inhibition constants were obtained.

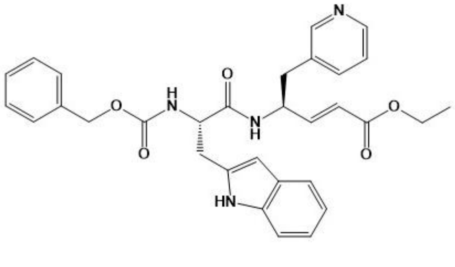

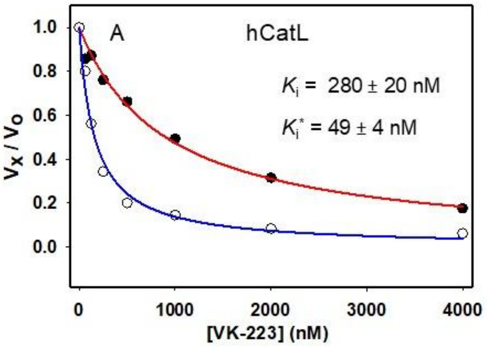

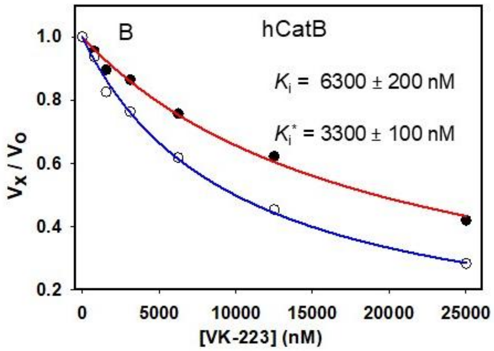

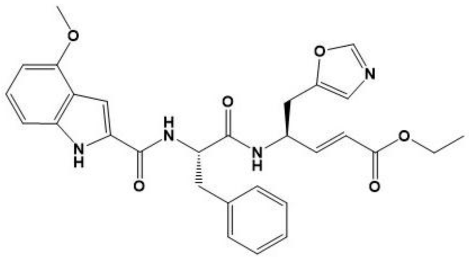

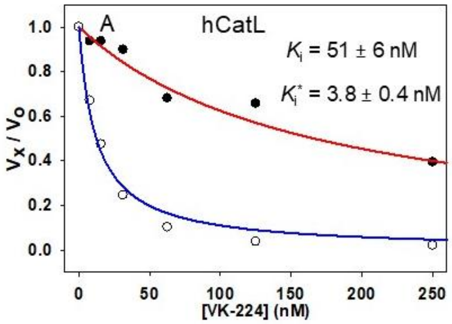

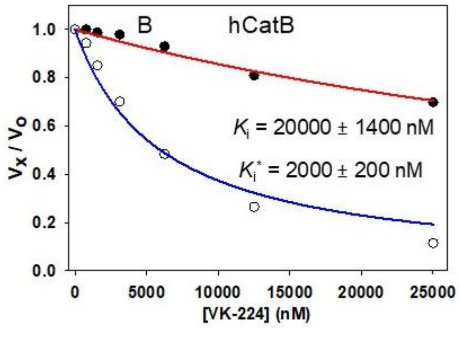

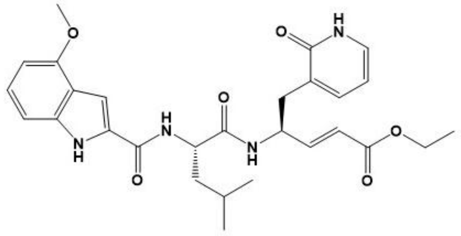

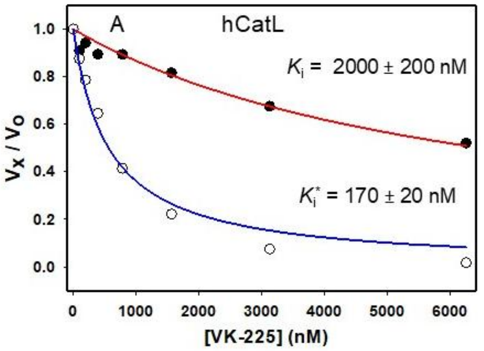

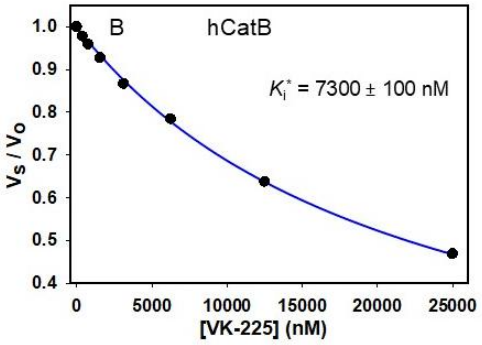

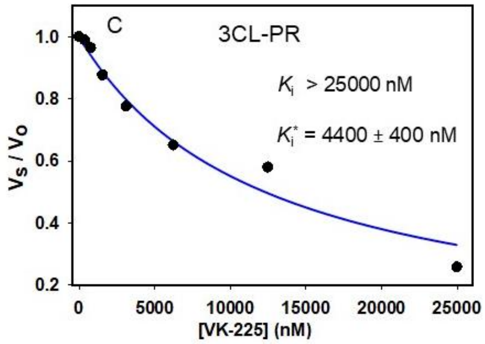

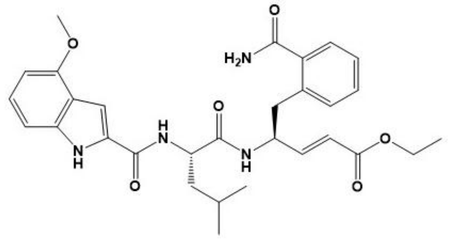

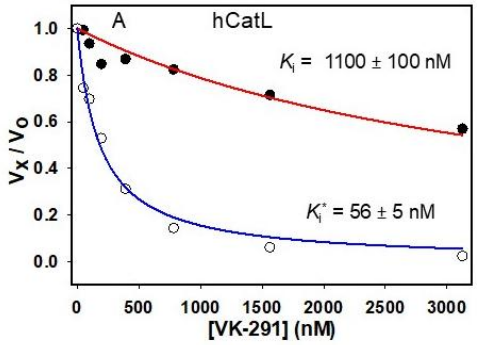

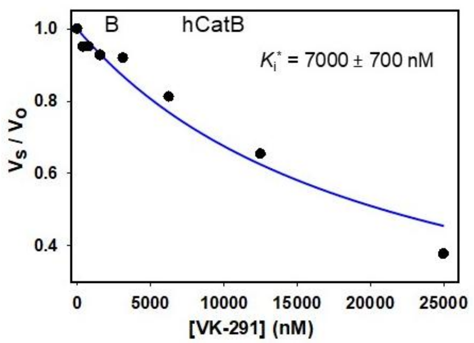

Figure S3. Compound structures and Cheng-Prusoff plots of **VK-223**, **K-224, VK-225**, and **VK-291**. Plotting of the fraction of remaining enzyme activity (v_x/v_0) vs. inhibitor concentrations at initial rates (t = 0-180 s, (v_i / v_0)) and steady-state rates (t = 1820-2000 s, (v_s / v_0)) for hCatL and hCatB**.** The lines drawn through the

experimental data points resulted from fitting of data to eq 7 and 8, from which the inhibition constants were obtained.

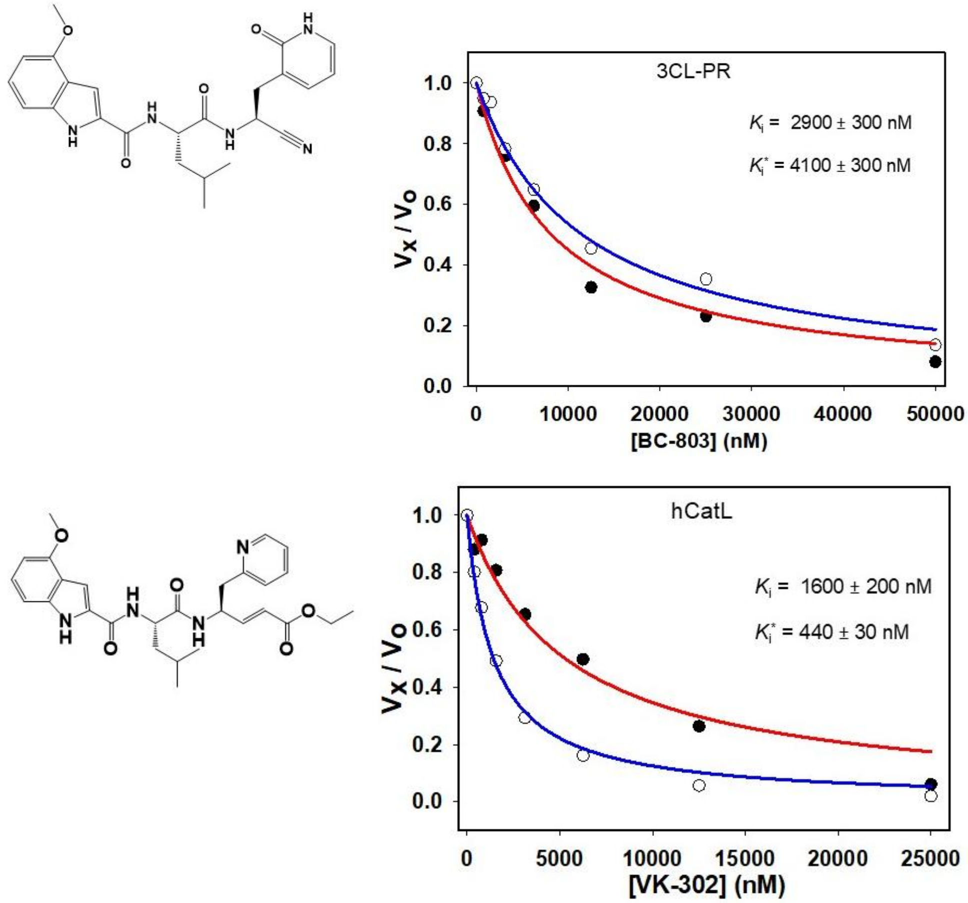

Figure S4. Compound structures and Cheng-Prusoff plots of **BC-803** vs 3CL-PR and **VK-302** vs hCatL. Plotting of the fraction of remaining enzyme activity (v_x/v_0) vs. inhibitor concentrations at initial rates (t = 0-180 s, (v_i / v_0)) and steady-state rates (t = 1820-2000 s, (v_s / v_0)) for 3CL-PR and hCatL**.** The lines drawn through the experimental data points resulted from fitting of data to eq 7 and 8, from which the inhibition constants were obtained.

Inhibition of VK-225 and BC-803 vs 3CL-PR. **VK-225** and **BC-803** share same compositions of P_1, P_2 and P_3 subgroups and differs in their P_1' covalent warheads. **VK-225** which has vinyl ester displayed time-dependent inactivation of 3CL-PR with more than 10-fold lowering of K_i in 30-min assay with K_i^* = 4400 ± 400 nM. Most likely, poor K_i^* indicates poor binding of **VK-225** at 3CL-PR active site and/or poor stabilization of enolate intermediate by S_1' pocket of 3CL-PR. **BC-803** with nitrile warhead displayed reversible inhibition of 3CL-PR **(Fig. 4)**. Unlike nirmatrelvir (K_i^* = 2.9 ± 0.1 nM), **BC-803** is poor inhibitor of 3CL-PR with K_i^* = 4100 ± 300 nM most likely resulting from poor binding of **BC-803** unlike nirmatrelvir.

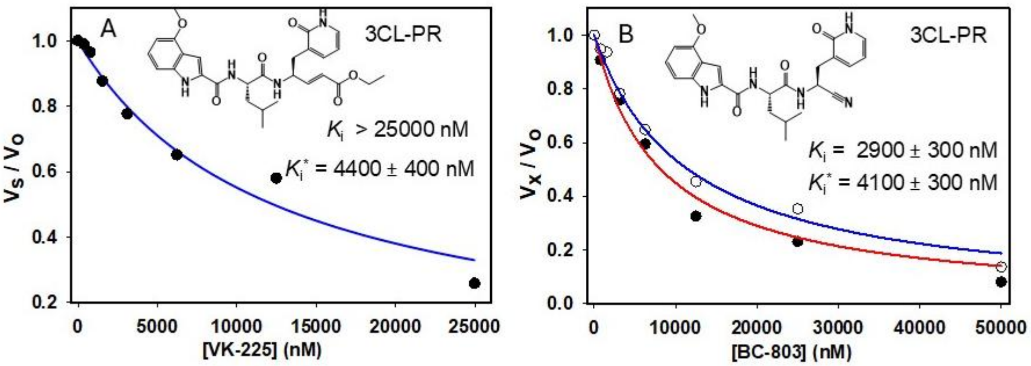

Figure S5: Inhibition of 3CL-PR by compounds VK-225 **(A)** and BC-803 **(B)**. Plotting of the fraction of remaining enzyme activity (v_x/v_0) vs. inhibitor concentrations at initial rates (t = 0-180 s, (v_i / v_0)) and steady-state rates (t = 1820-2000 s, (v_s / v_0)) for 3CL-PR.The lines drawn through the experimental data points resulted from fitting of data to eq 6 and 7, from which the inhibition constants were obtained.

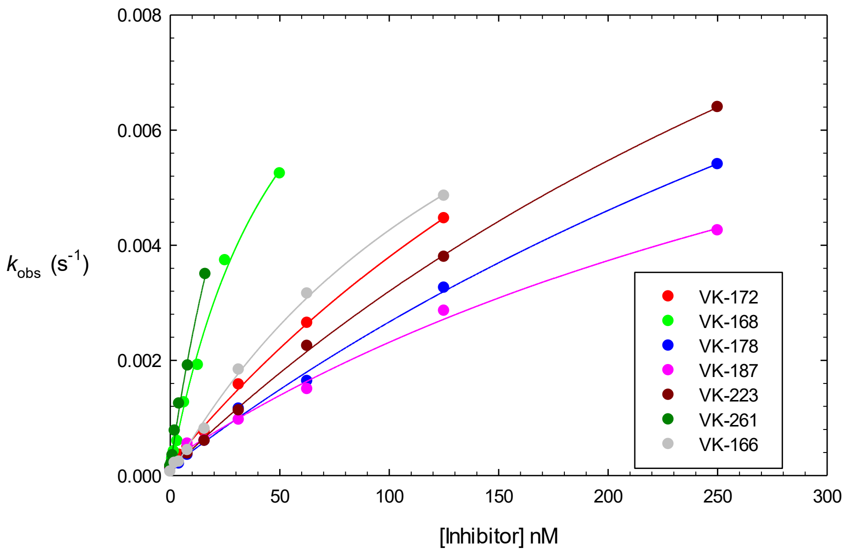

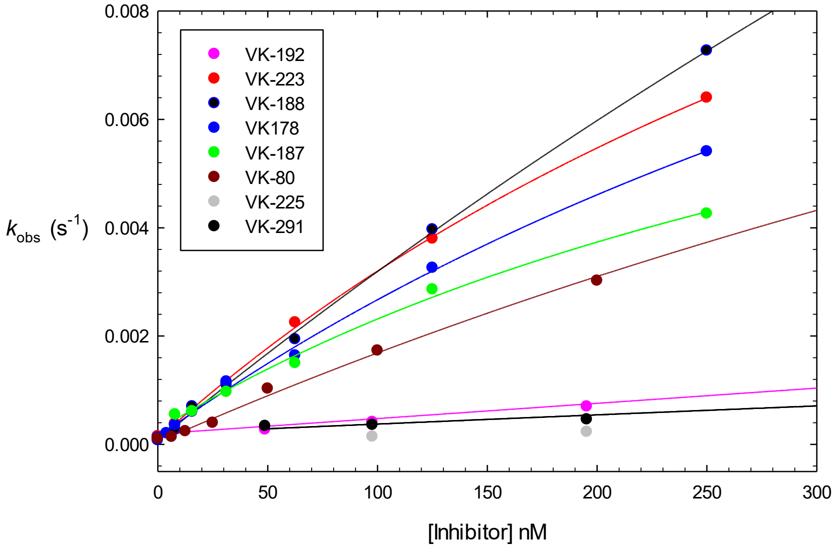

Figure S6. Re-plots of k_{obs} vs. [inhibitor]. All data were fitted to both eqs 9 and 10, with the best fittings found in **Table S1**. (**Top**) The lines drawn through the data points were from fitting of data to eq 9, with results of fittings found in **Table S1**; (**Bottom**). The lines drawn through the data points were from fitting of data to eq 9, with the exception of **VK-192**, which was best fitted to eq 10, with results of fittings found in **Table S1**.

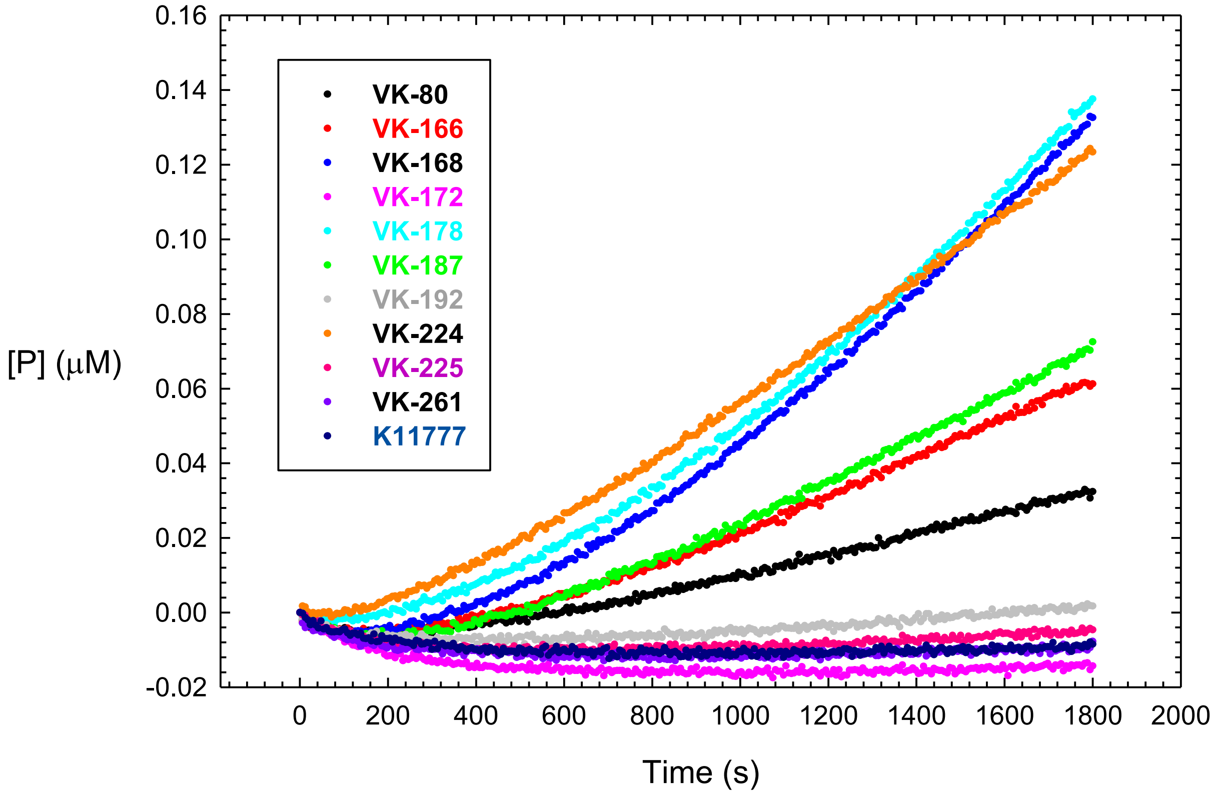

Figure S7. Time courses of residual enzyme activities of cathepsin L (100 nM in pre-incubation mixture) following 12-hour incubation with excess concentrations of inhibitors (500 nM) prior to 100-fold dilution into a reaction mixture containing 20 mM Cbz-Leu-Arg-AMC. **VK-80** (black), **VK-166** (red), **VK-166** (blue), **VK-172** (magenta), **VK-178** (cyan), **VK-187** (light green), **VK-192** (gray), **VK-224** (orange), **VK-225** (maroon), **VK-261** (dark magenta), and **K11777** (dark blue).

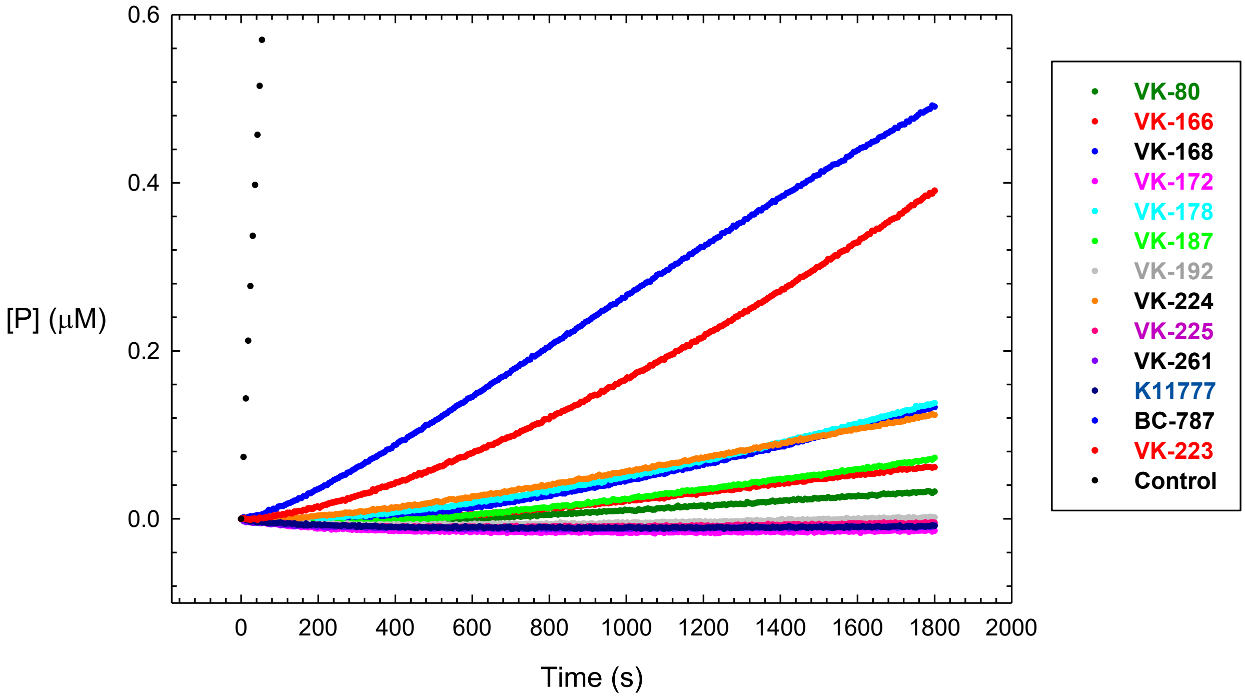

Figure S8. Time courses of residual enzyme activities of cathepsin L (100 nM in pre-incubation mixture) following 12-hour incubation with excess concentrations of inhibitors (500 nM) prior to 100-fold dilution into a reaction mixture containing 20 mM Cbz-Leu-Arg-AMC. **Control** (black points) **VK-80** (dark green), **VK-166** (red), **VK-166** (blue), **VK-172** (magenta), **VK-178** (cyan), **VK-187** (light green), **VK-192** (gray), **VK-224** (orange), **VK-225** (maroon), **VK-261** (dark magenta), and **K11777** (dark blue).

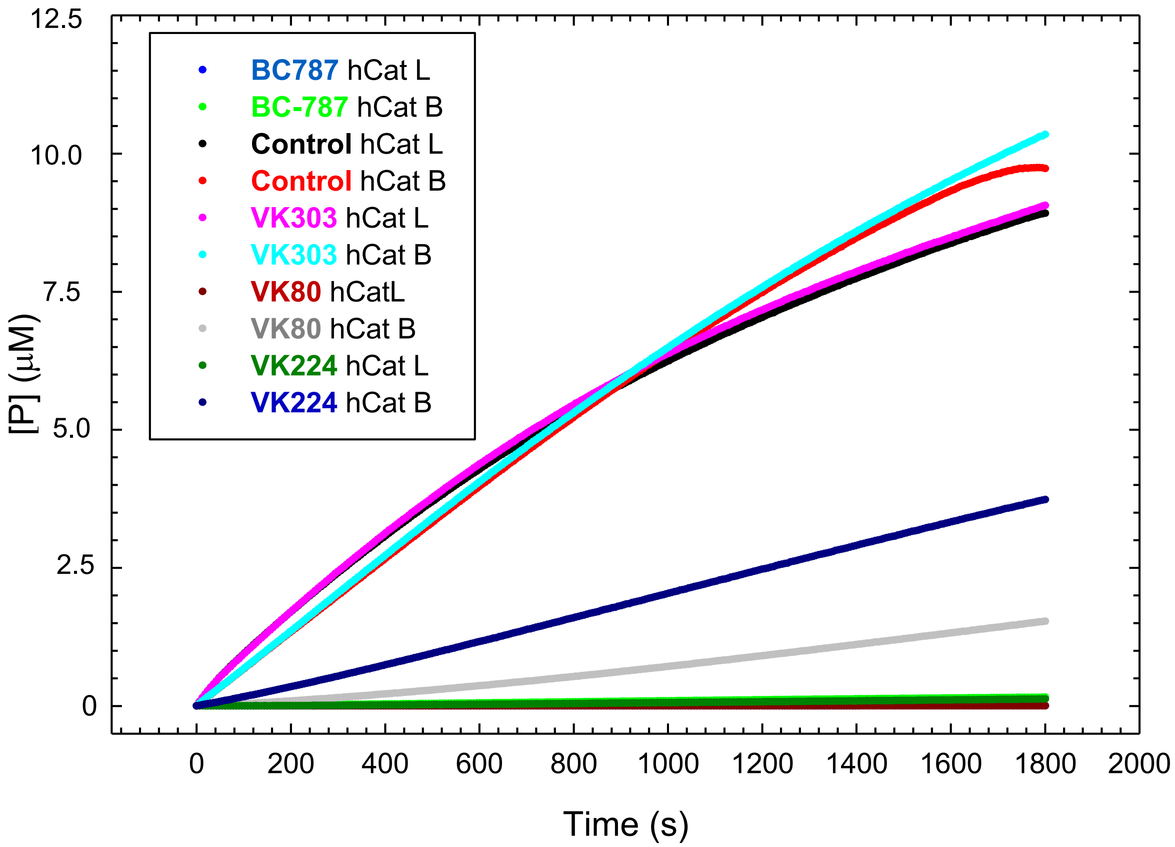

Figure S9. Time courses of residual enzyme activities of cathepsin L (100 nM in pre-incubation mixture) and cathepsin B (250 nM in pre-incubation mixture) following 12-hour incubation with excess concentrations of inhibitors (500 nM for Cat L and 2500 nM for Cat B), followed by 100-fold dilution into a reaction mixture containing 20 mM (Cat L) and 50 mM (Cat B) Cbz-Leu-Arg-AMC. **Control sample** (DMSO): Cat L (black), Cat B (red); **BC-787**: Cat L (blue), Cat B (light green); **VK-303** Cat L (magneta), Cat B (cyan).**VK-224**: Cat L (dark green), Cat B (dark blue).

Figure S10. Anti-CoV-2 Data for hCatL Inhibitors in SARS-CoV-2-Infected Vero E6 Cells and A549/ACE2 cells. [a]

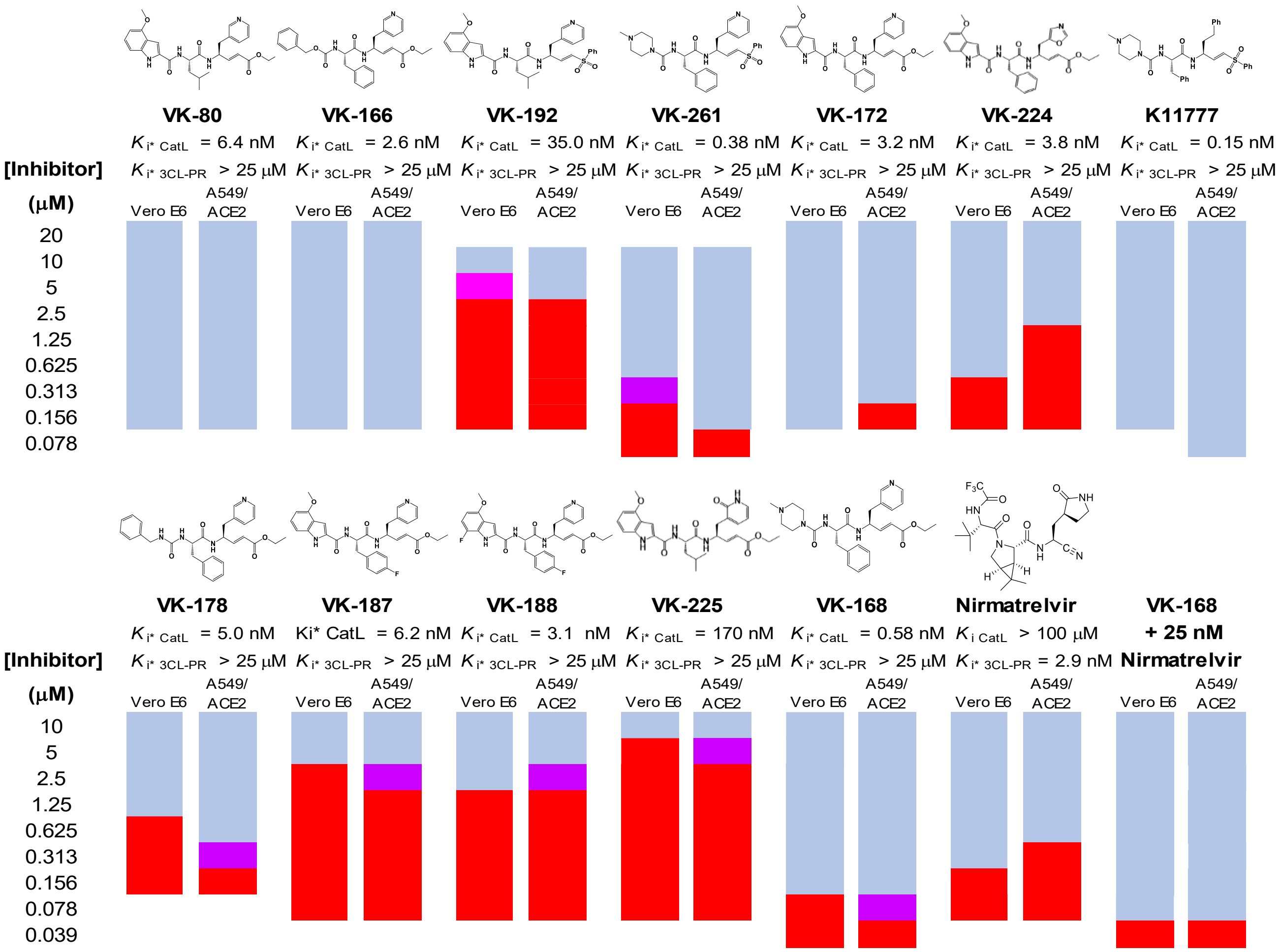

[a]Samples contained 1% (v/v) DMSO. 2 µM CP-100356 inhibitor was included for Vero-E6 cells. Red indicates the presence of the viral cytopathic effect while blue indicates its absence. The experiment was done in duplicate. Purple indicates CPE and no CPE at the same concentration.

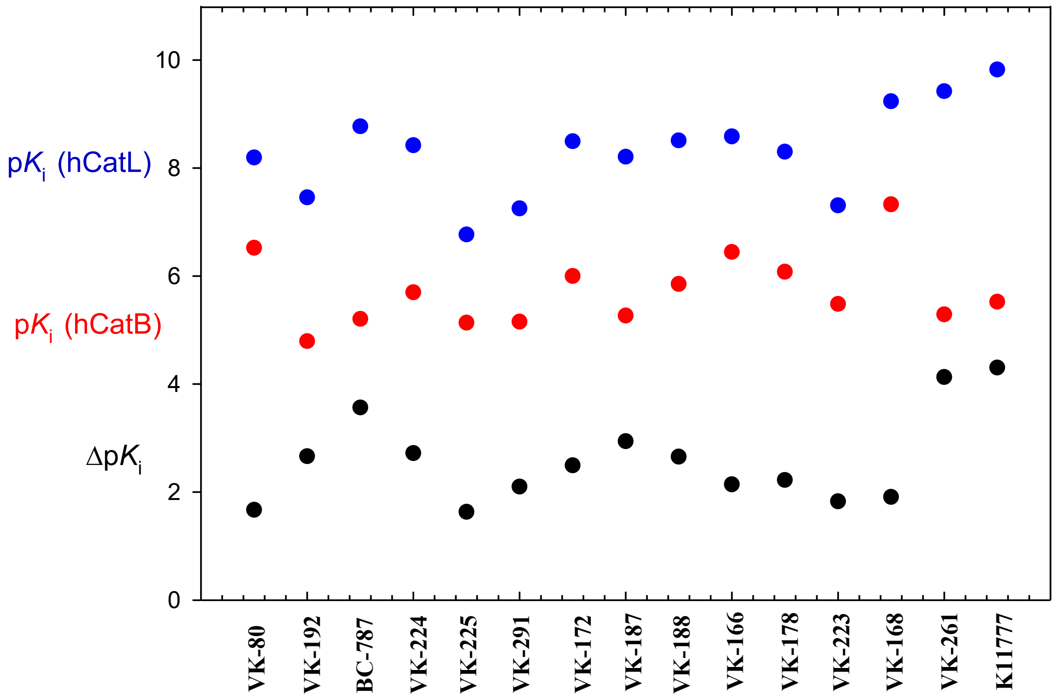

Figure S11. Values of pK_i for hCatL (blue), hCatB (red) and pK_i (hCatL) - pK_i(hCatB) (DpK_i) vs. compounds found in **Table 1**.

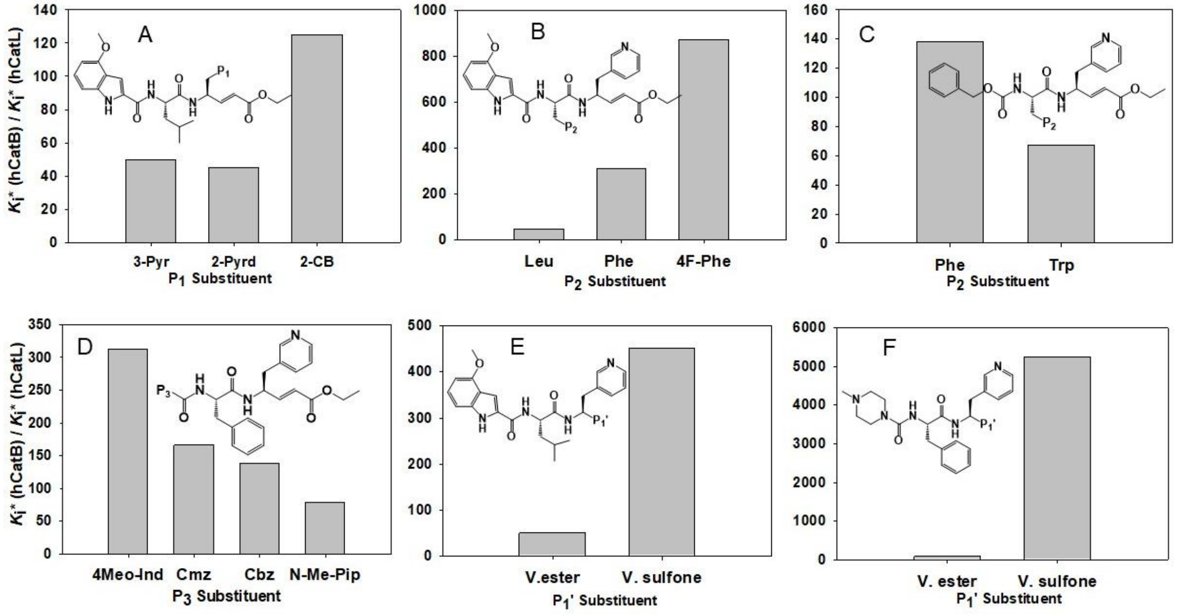

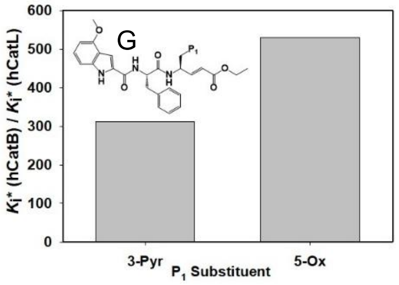

Figure S12. Bar graphs showing the comparison of selectivity ratios (K_i^*(hCatB) / K_i^*(hCatL) of P_1, P_2, P_3 and P_1' groups while keeping other groups of the peptidomimetics constant.

Molecular Docking:

Structures of the compounds **VK-80**, **VK-166**, **VK-168**, and **VK-172** in the active site of hCatL resulted in docking scores of -5.659, -5.634, -6.347, and -5.967 respectively. Sidechains from each of the bound ligands occupied the $S_{1'}$, S_1, S_2, and S_3 binding subsites. Hydrogen bonds observed between the active-site residues and ligand are as seen in **Fig. 6**. Five hydrogen bonds defined the binding of **VK-80** (Gln19-O5, Trp189-O5, Asp162-N3, Gly68-N1, and Gly68-O3) and **VK-172** (Gln19-O5, Trp189-O5, Asp162-N3, Gly68-N1, and Gly68-O3) (**Figs. 6A**, **6B**), with similar hydrogen bonding for **VK-166** (Gln19-O5, Trp189-O5, Asp162-N2, Gly68-O3, and Gly68-N1 (**Fig. 6C**) The 5 hydrogen bonds observed with bound **VK-168** were Gln19-O4, Trp189-O4, Asp162-N3, Gly68-N2 and Gly68-O2 (**Fig. 6D**). Non-bonded contacts and representative hydrophobic interactions are included in **Fig. S12**.

Modeled structures bound similarly into all sub-sites with variations in rotation and minor changes in position. In the oxyanion hole of the $S_{1'}$ position the structures of **VK-80**, **VK-166**, **VK-168**, and **VK-172** were overlapping, with differences in the orientations of the terminal carbons of **VK-80**, **VK-166**, and **VK-172**. **VK-168** is positioned with its terminal carbon, C17, toward solvent. For all compounds the carbonyl oxygens of the α,β-unsaturated esters were found in the oxyanion hole of Gln19, Trp189, and His163, in which hydrogen bonds were indicated for all three compounds (**VK-80**:Gln19-O5 2.86 Å, Trp189-O5 3.32 Å; **VK-166**: Gln19-O5 2.91 Å, Trp189-O5 3.26 Å; **VK-168**: Gln19-O5 2.99 Å, Trp189-O4 3.30 Å; **VK-172**: Gln19-O5 2.93 Å, Trp189-O5 3.27 Å) (**Fig. S11**).

The 3-pyridine groups of all compounds occupied the S_1 sub-site, situated above Cys_{25}, with the ring positioned above Gly_{23}, with the exception of **VK-166**. **VK-168** was flipped, positioning N4 toward Asn_{66} with a proposed hydrophobic interaction not seen in the other compounds. The ligand 3-pyridine backbone nitrogen was positioned at the entrance of the S_2 subsite with a proposed hydrogen bond to the backbone oxygen of Asp162 (**VK-80**: 2.67 Å, **VK-166**: 2.85 Å, **VK-168**: 2.70 Å, **VK-172**: 2.67 Å). (**Fig. S11**)

The S_2 subsite of hCatL generally accommodates bulky, hydrophobic sidechains. The P_2 groups in the ligands, Leu in **VK-80**, Phe in **VK-166**, **VK-168**, and **VK-172**, all bound into a hydrophobic pocket with primary interactions with Leu69, Ala135, Met161, Gly164, and Ala214 (**Fig. S11**).

Binding of the P_3 groups consisted of hydrophobic interactions (Trp_{26} and Tyr_{72}) and a preference for hydrogen bonds along the backbone of residues Gly_{67}-Gly_{68}-Leu_{69}. For **VK-80**, the 4-methoxy-tryptophan was positioned laterally with the methoxy group pointed towards solvent. **VK-166** has its Cbz group positioned deep inside the S_3 pocket with interactions indicated between the Cbz carbons and Tyr_{72}, Glu_{63}, and Gly_{61}. The 4-methyl-piperazine of **VK-168** was positioned at the entrance of the S_3 pocket, with the N5 side of the piperazine positioned toward the solvent, and interactions with its terminal carbon and the residues Glu_{63} and Gly_{61}. **VK-172** has its 4-methoxy-tryptophan positioned deeper than **VK-80**, with interactions from residues Trp_{26}, Gly_{61}, Glu_{63}, Leu_{69}, and Tyr_{72}. The backbone oxygens and nitrogens of the S_2 and S_3 compounds S2-S3 are proposed to hydrogen bond with Gly_{68} (**VK-80**: O- 2.95 Å, N- 2.78 Å; **VK-166**: O-2.91 Å, N-2.78 Å; **VK-168**: O-3.02 Å, N- 2.83 Å; **VK-172**: O-3.07 Å, N-2.80 Å) (**Fig. S11**).

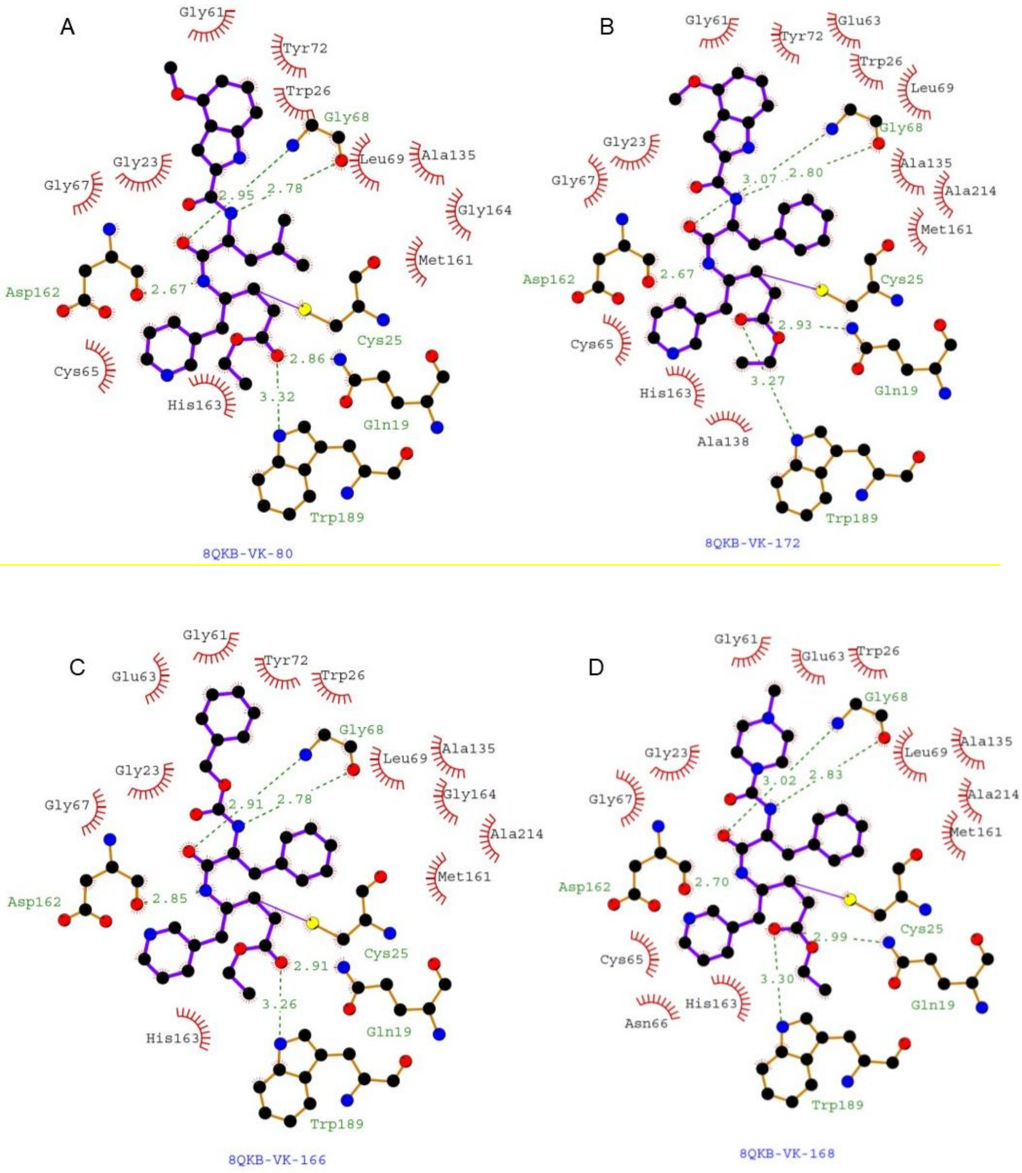

Figure S13: LigPlot diagrams of covalently-docked compounds bound to hCatL structure 8QKB. Hydrogen and covalent bonds are indicated as dotted green and solid purple lines, respectively. Distances are indicated as distances in Å on their respective bonds. Residues that hydrogen bond to the docked compound are labeled in green while residues with hydrophobic interactions are labeled in black. (A) **VK-80**, (B) **VK-172**, (C) **VK-166**, (D) **VK-168**.

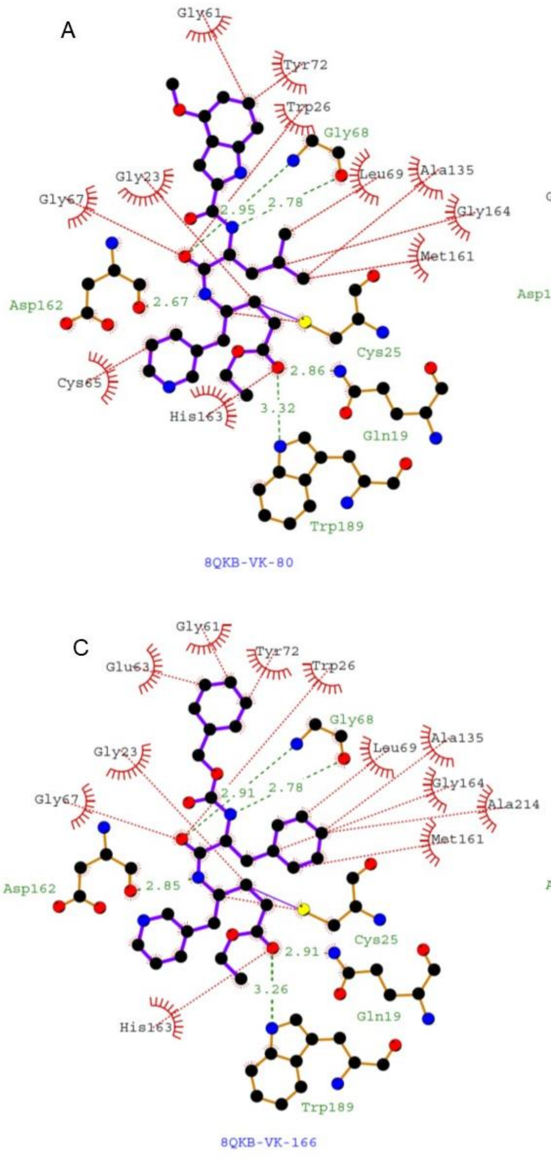

Figure S14: LigPlot diagrams of covalently docked compounds to hCatL structure 8QKB. Hydrogen and covalent bonds are indicated as dotted green and solid purple lines respectively. Distances are indicated in Å on their respective bonds. Representative hydrophobic interactions are shown as solid red lines. Residues that hydrogen bond to the docked compound are labeled in green while residues with hydrophobic interactions are labeled in black. (A) **VK-80**, (B) **VK-172**, (C) **VK-166**, (D) **VK-168**.

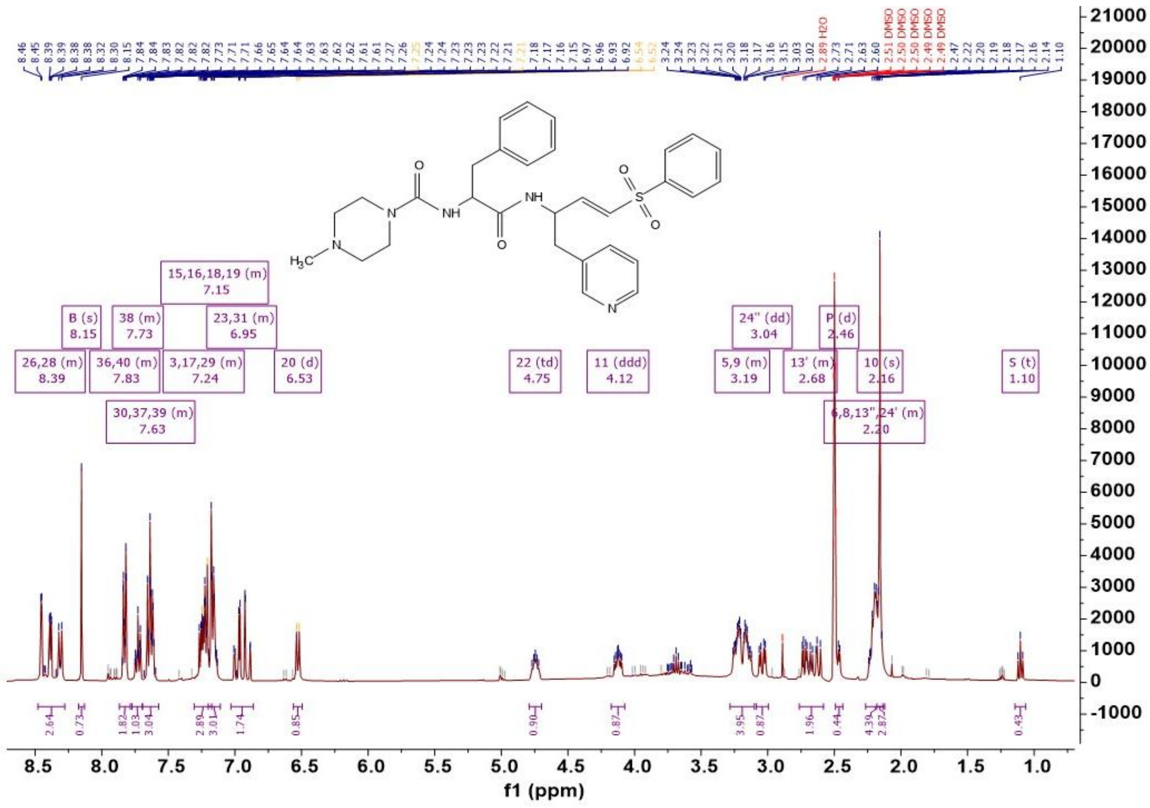

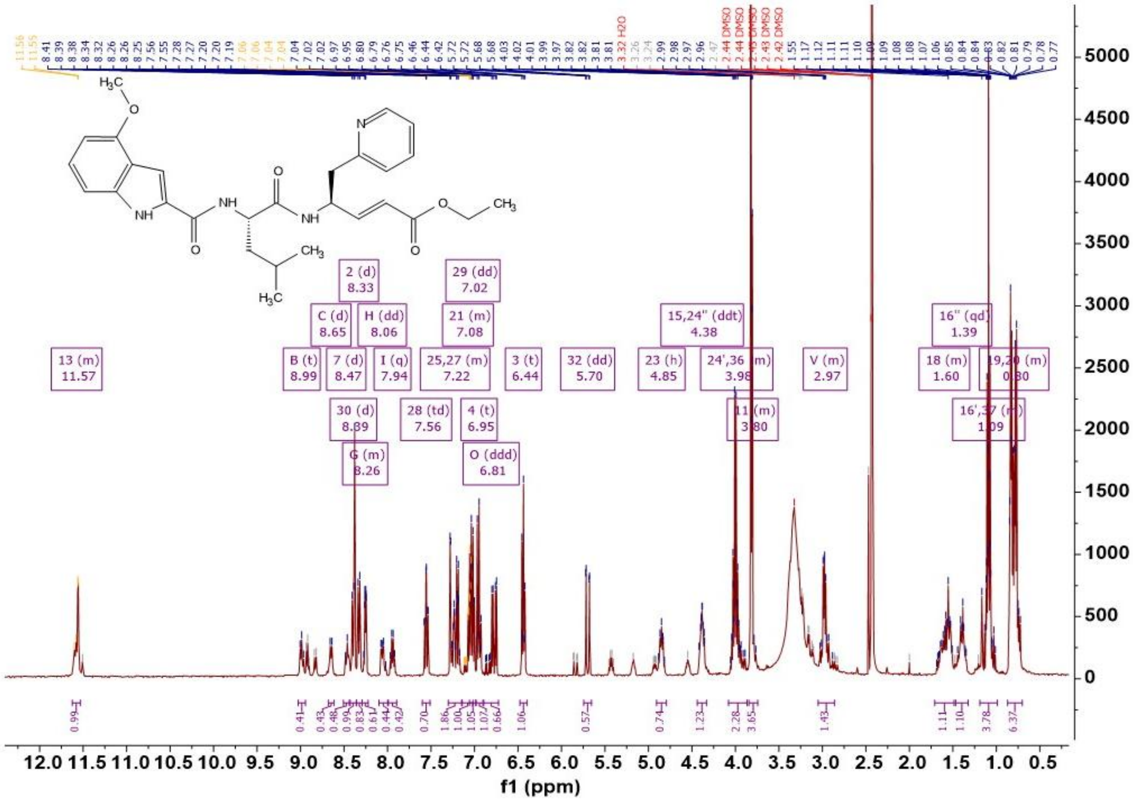

f1 (ppm)

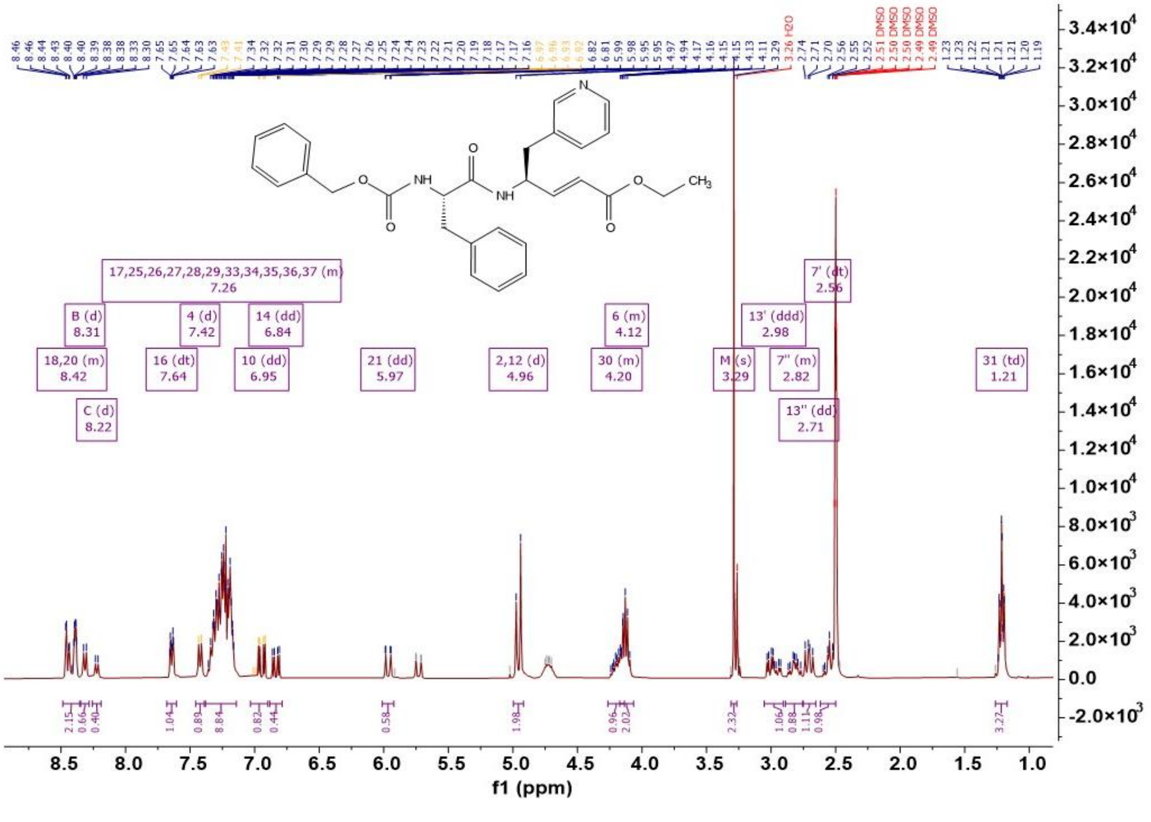

f1 (ppm)

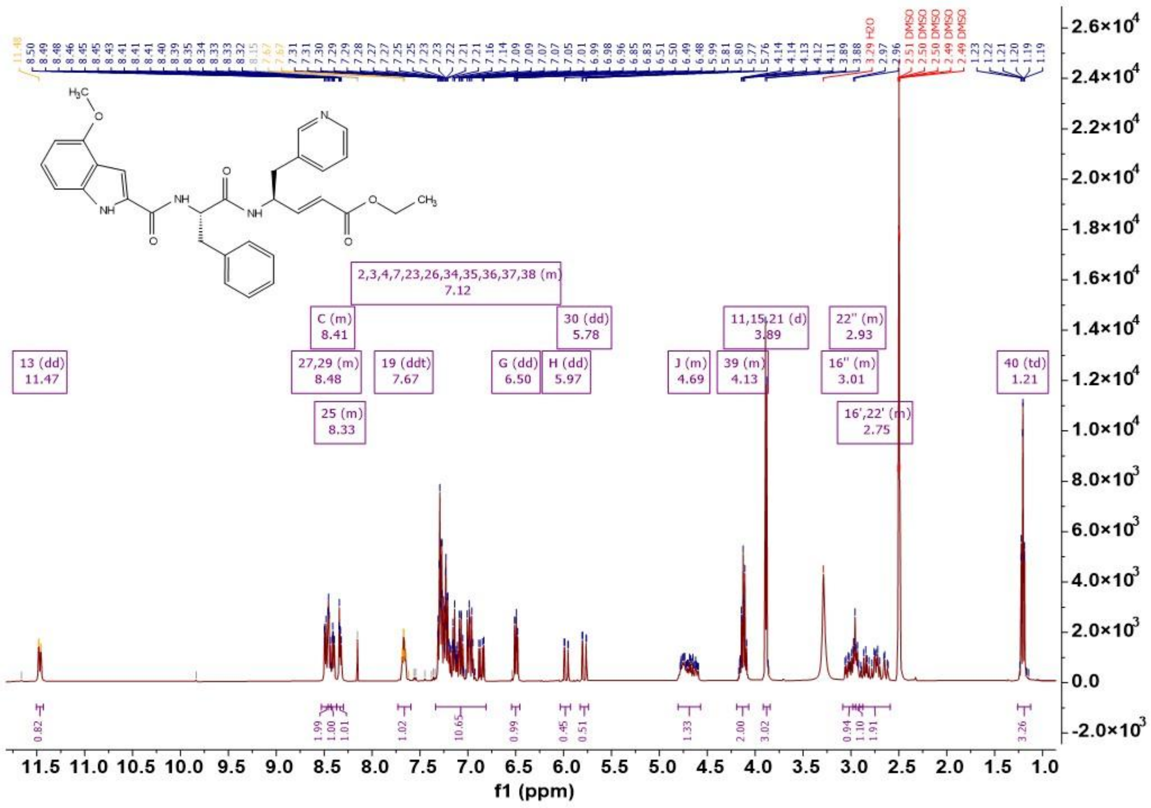

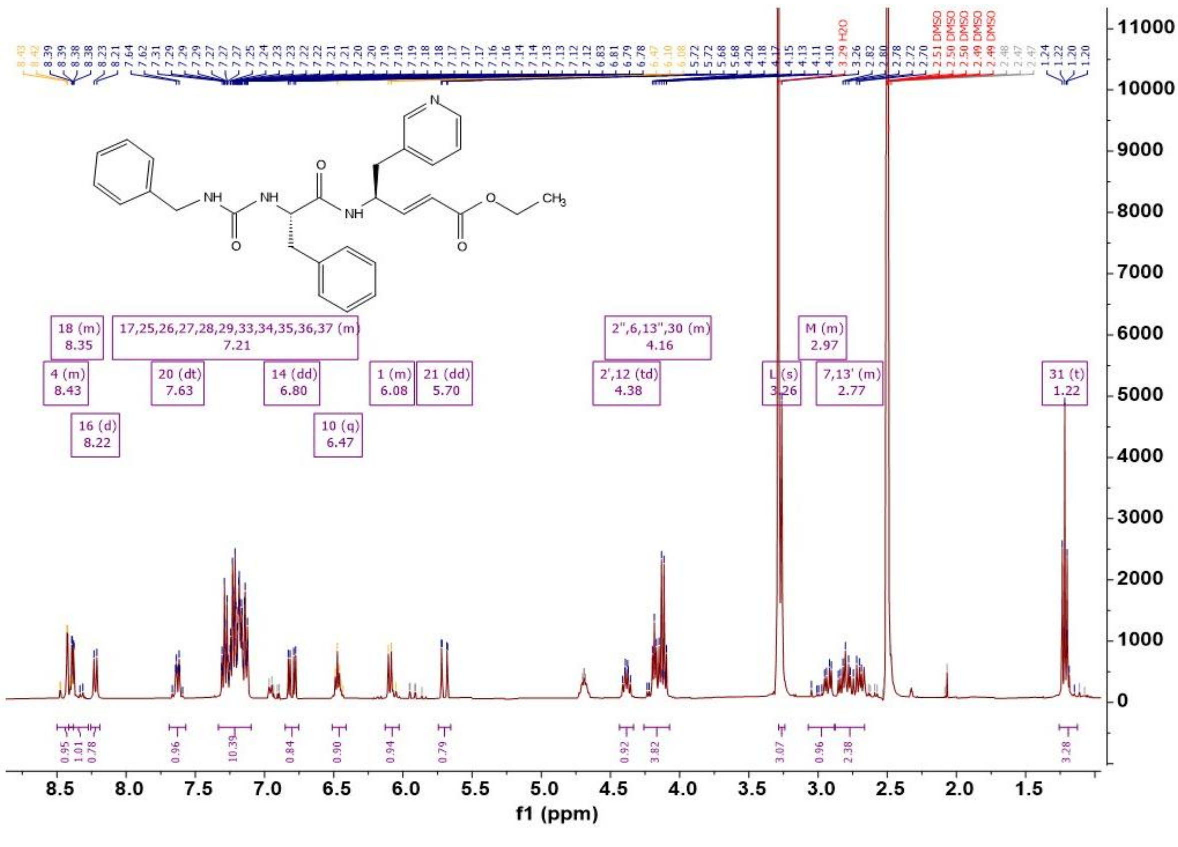

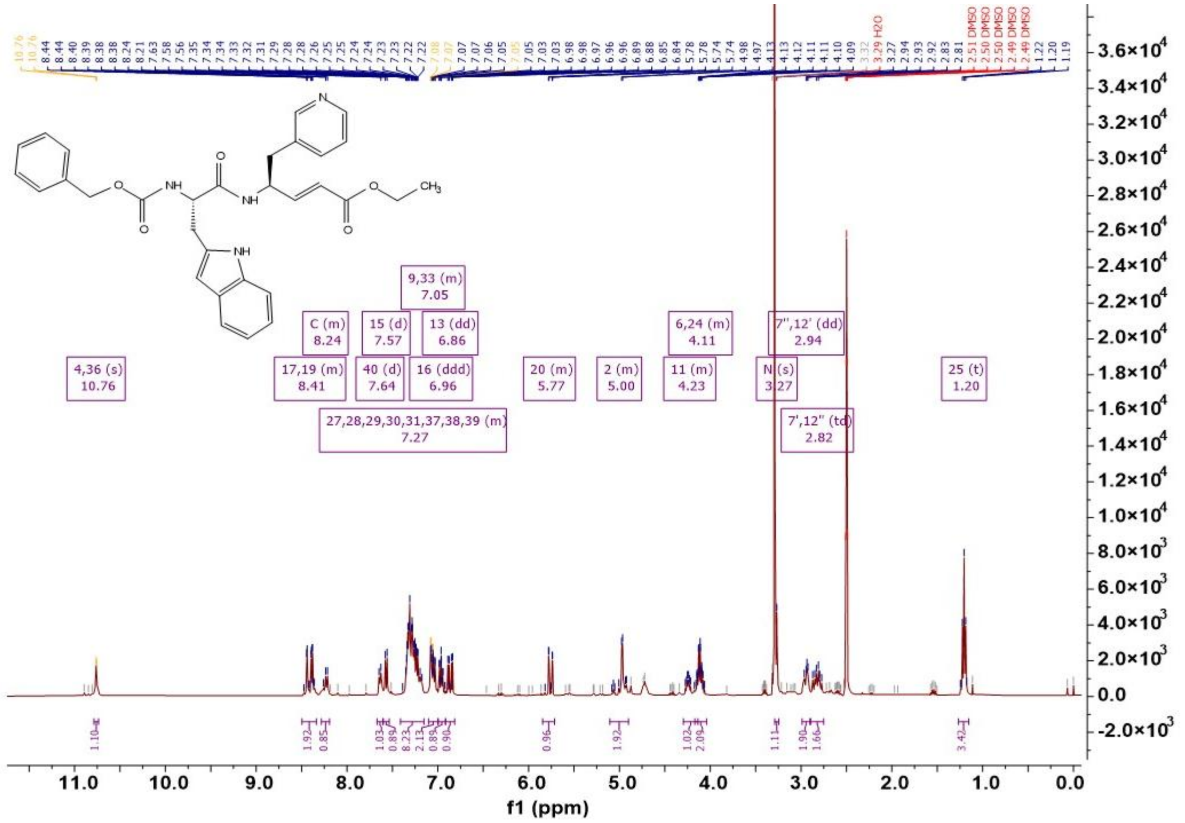
9,33 (m)
7.05
C (m)
8.24
15 (d)
7.57
13 (dd)
6.86
6,24 (m)
4.11
7'',12' (dd)
2.94
4,36 (s)
10.76
17,19 (m)
8.41
40 (d)
7.64
16 (ddd)
6.96
20 (m)
5.77
2 (m)
5.00
11 (m)
4.23
N (s)
3.27
25 (t)
1.20
27,28,29,30,31,37,38,39 (m)
7.27
7',12'' (td)
2.82
f1 (ppm)

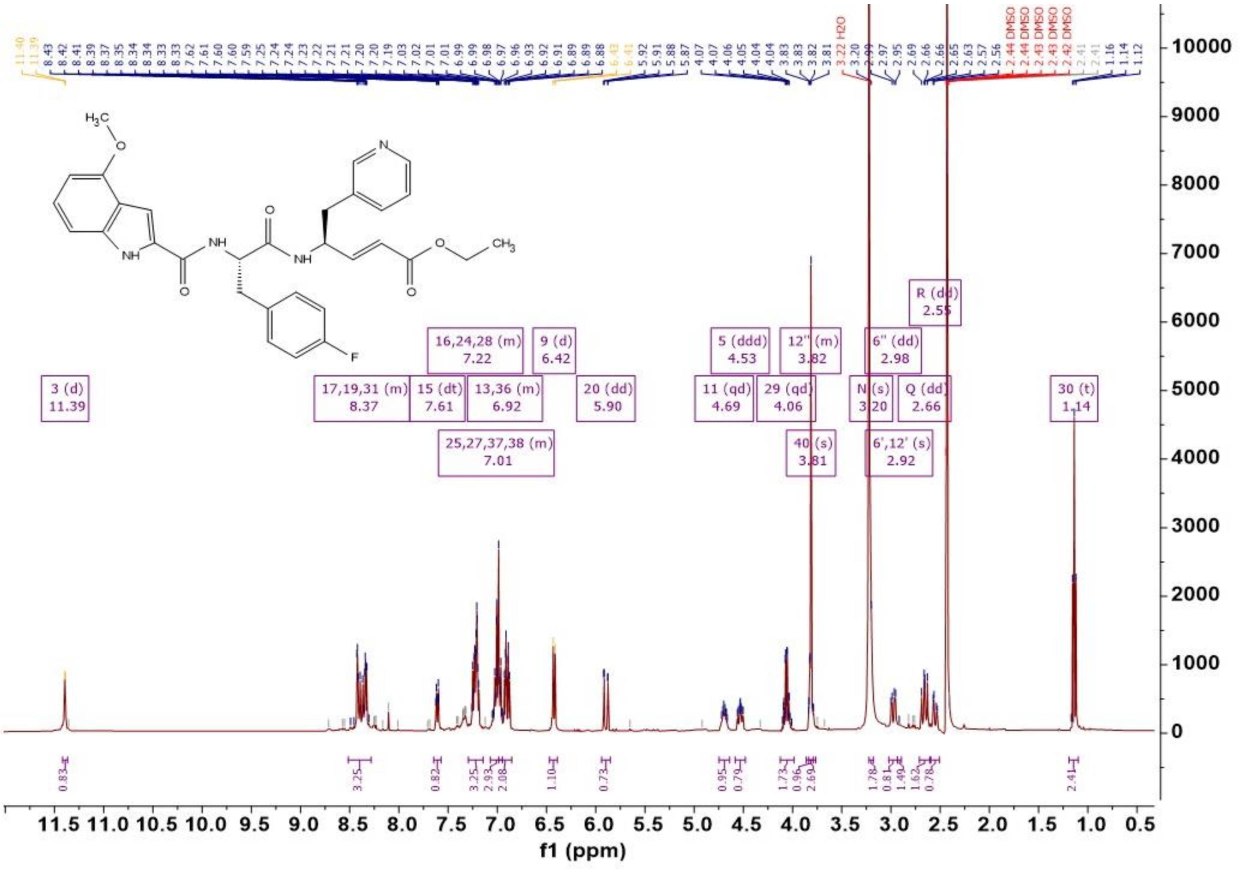
R (dd)
2.55
16,24,28 (m)
7.22
9 (d)
6.42
5 (ddd)
4.53
12'' (m)
3.82
6'' (dd)
2.98
3 (d)
11.39
17,19,31 (m)
8.37
15 (dt)
7.61
13,36 (m)
6.92
20 (dd)
5.90
11 (qd)
4.69
29 (qd)
4.06
N (s)
3.20
Q (dd)
2.66
30 (t)
1.14
25,27,37,38 (m)
7.01
40 (s)
3.81
6',12' (s)
2.92
f1 (ppm)

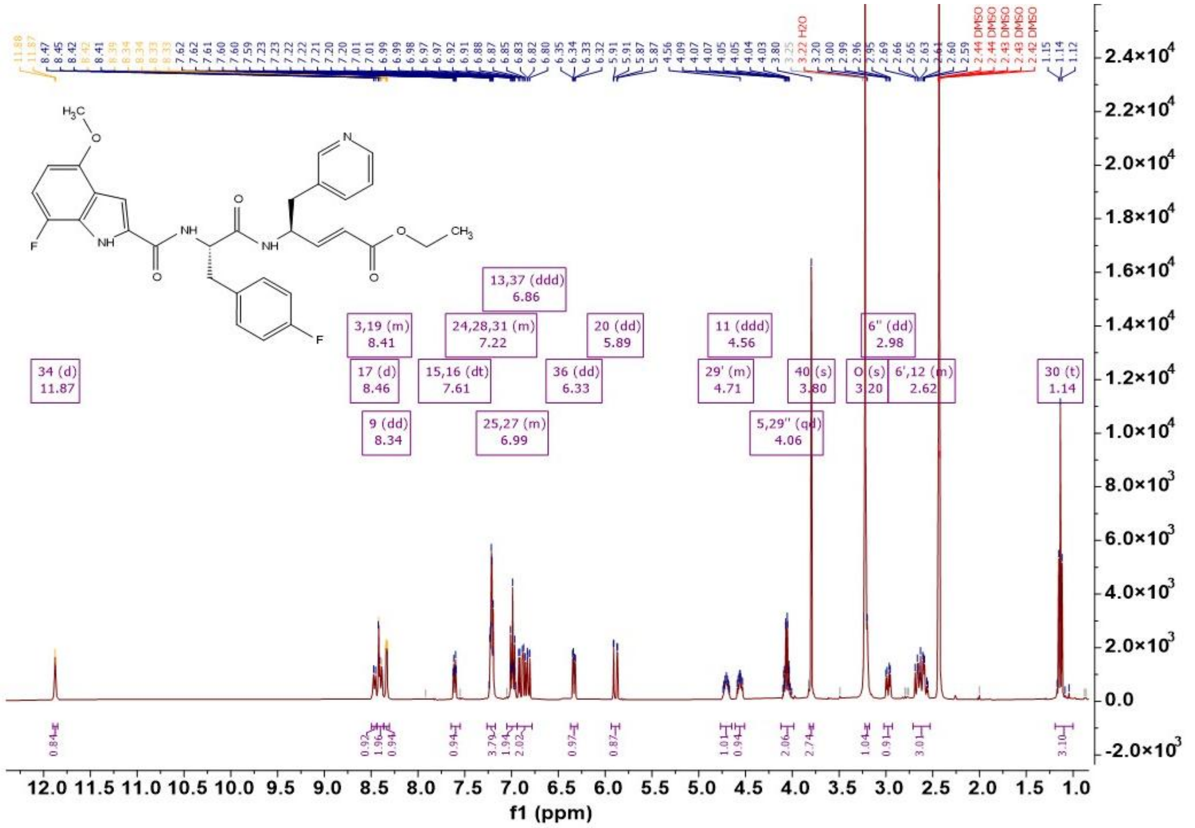
34 (d)
11.87
3,19 (m)
8.41
17 (d)
8.46
9 (dd)
8.34
15,16 (dt)
7.61
13,37 (ddd)
6.86
24,28,31 (m)
7.22
25,27 (m)
6.99
36 (dd)
6.33
20 (dd)
5.89
11 (ddd)
4.56
29' (m)
4.71
5,29'' (qd)
4.06
40 (s)
3.80
O (s)
3.20
6'' (dd)
2.98
6',12 (m)
2.62
30 (t)
1.14
f1 (ppm)

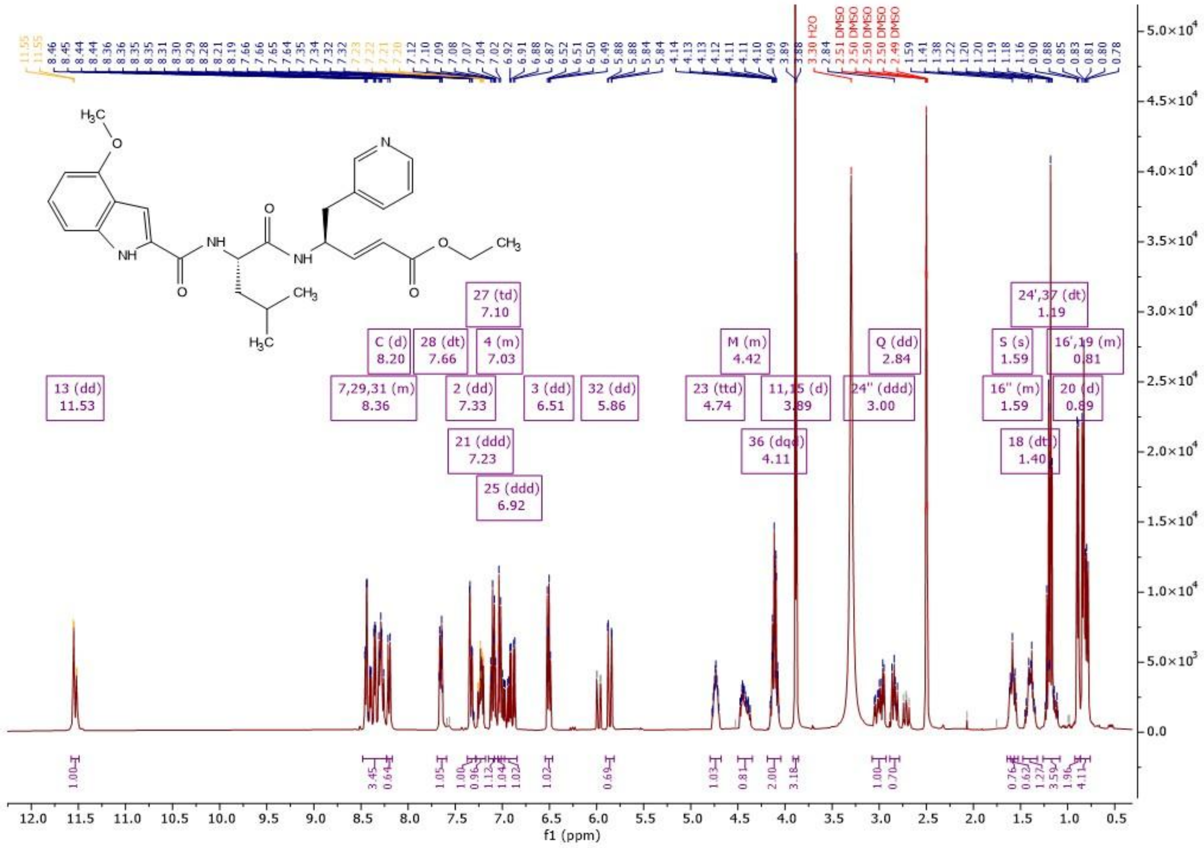
13 (dd)
11.53
C (d)
8.20
7,29,31 (m)
8.36
28 (dt)
7.66
27 (td)
7.10
4 (m)
7.03
2 (dd)
7.33
21 (ddd)
7.23
25 (ddd)
6.92
3 (dd)
6.51
32 (dd)
5.86
M (m)
4.42
23 (ttd)
4.74
11,15 (d)
3.89
36 (dqd)
4.11
Q (dd)
2.84
24'' (ddd)
3.00
S (s)
1.59
16'' (m)
1.59
18 (dt)
1.40
24',37 (dt)
1.19
16',19 (m)
0.81
20 (d)
0.89
f1 (ppm)

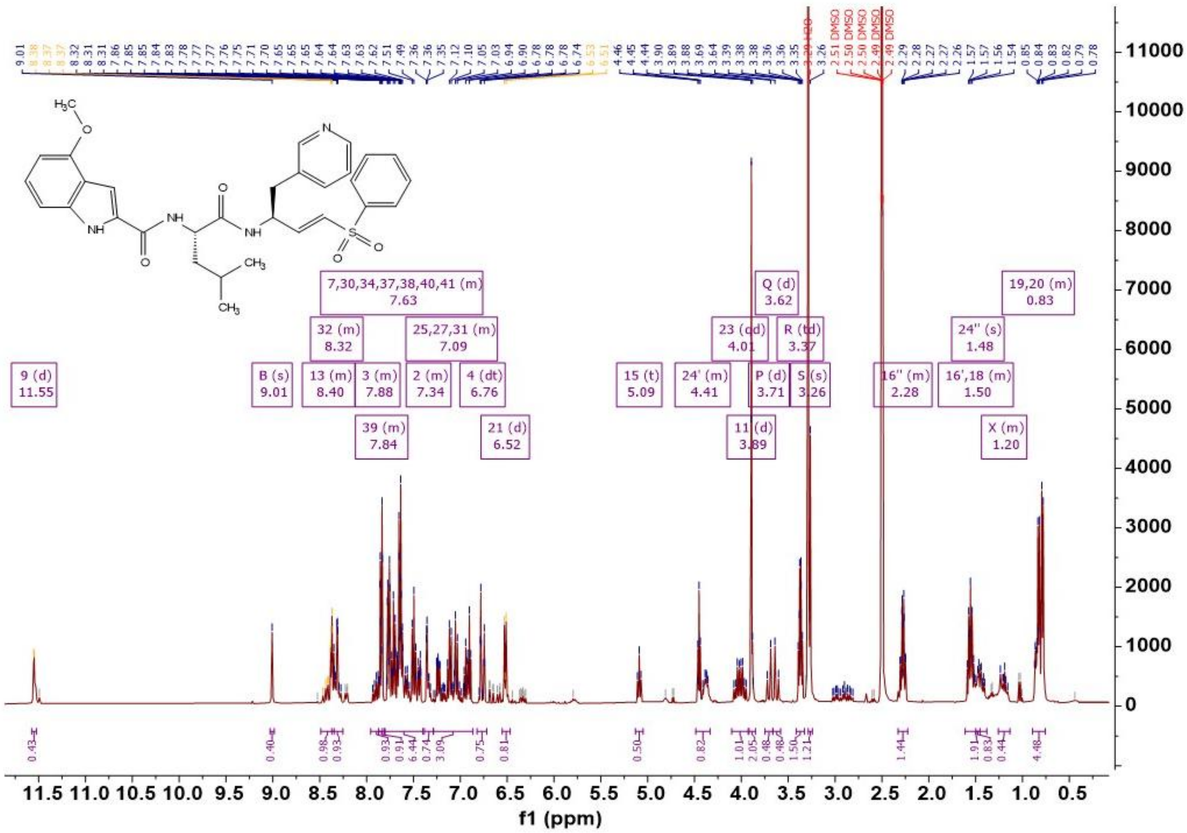

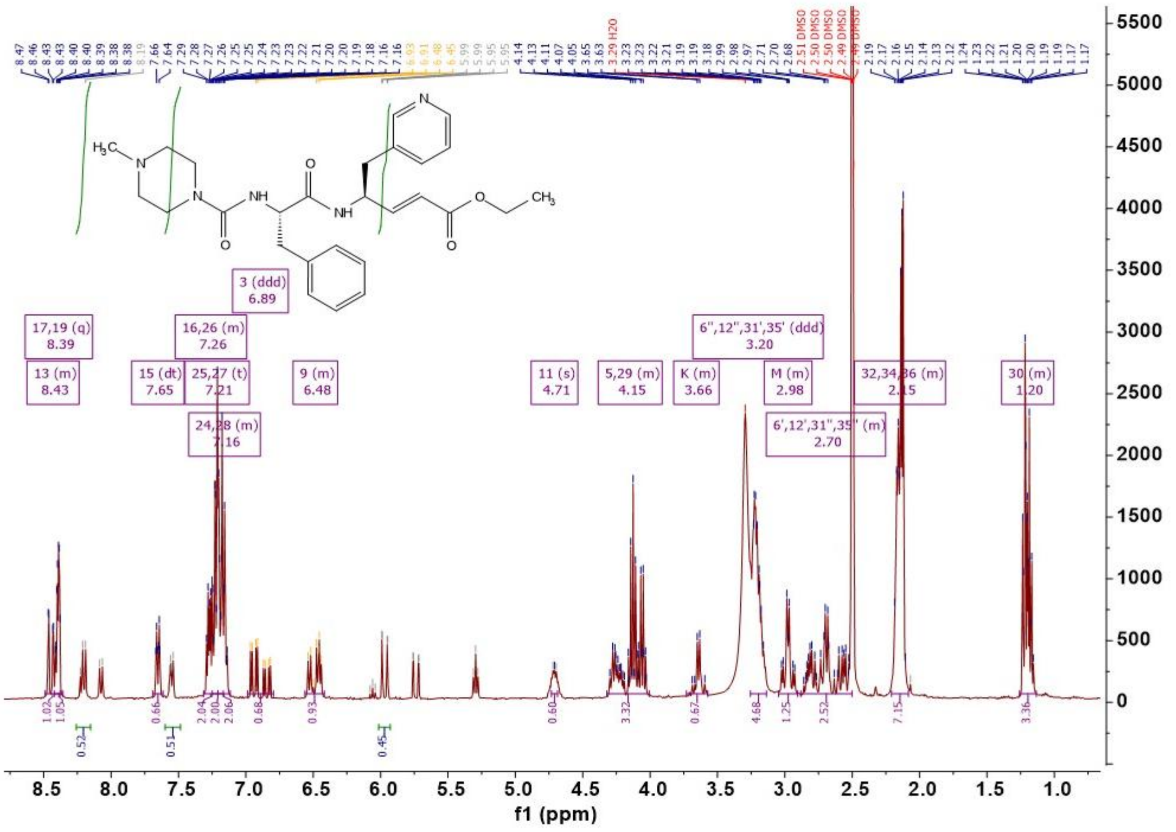

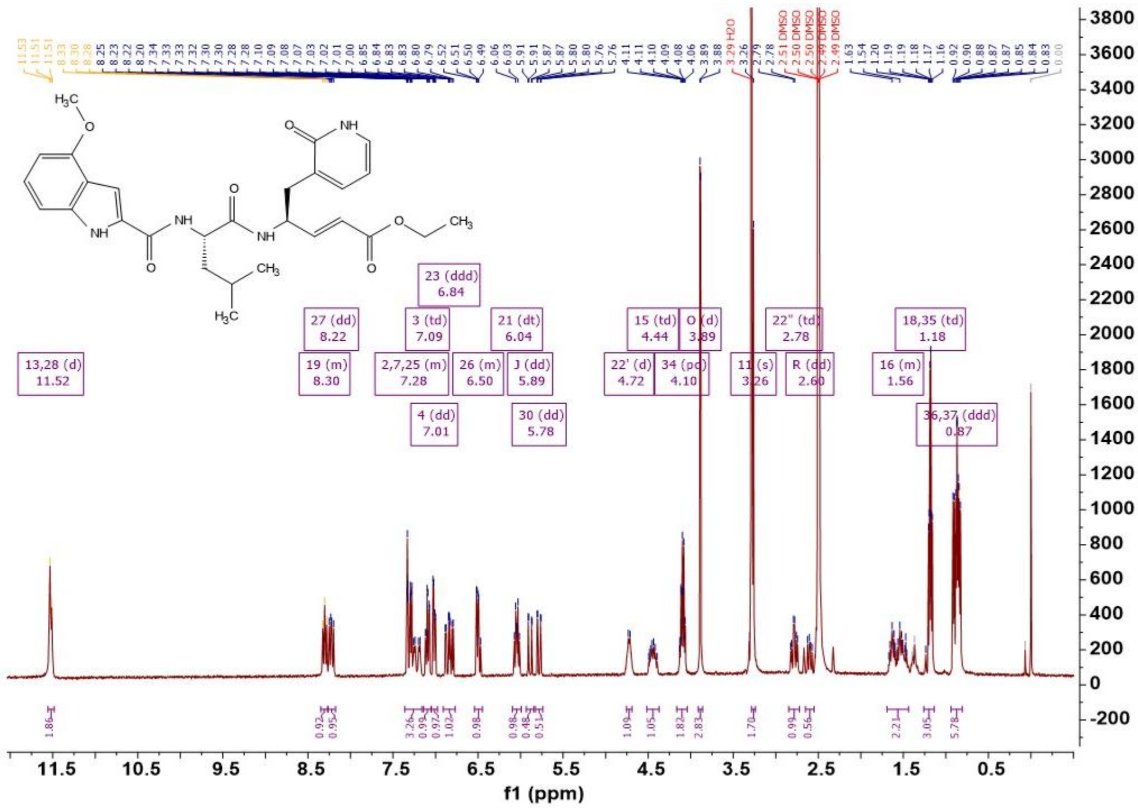
13,28 (d) 11.52
27 (dd) 8.22
19 (m) 8.30
23 (ddd) 6.84
3 (td) 7.09
2,7,25 (m) 7.28
4 (dd) 7.01
26 (m) 6.50
21 (dt) 6.04
J (dd) 5.89
30 (dd) 5.78
15 (td) 4.44
O (d) 3.89
22' (d) 4.72
34 (pd) 4.10
11 (s) 3.26
22'' (td) 2.78
R (dd) 2.60
16 (m) 1.56
18,35 (td) 1.18
36,37 (ddd) 0.87
f1 (ppm)

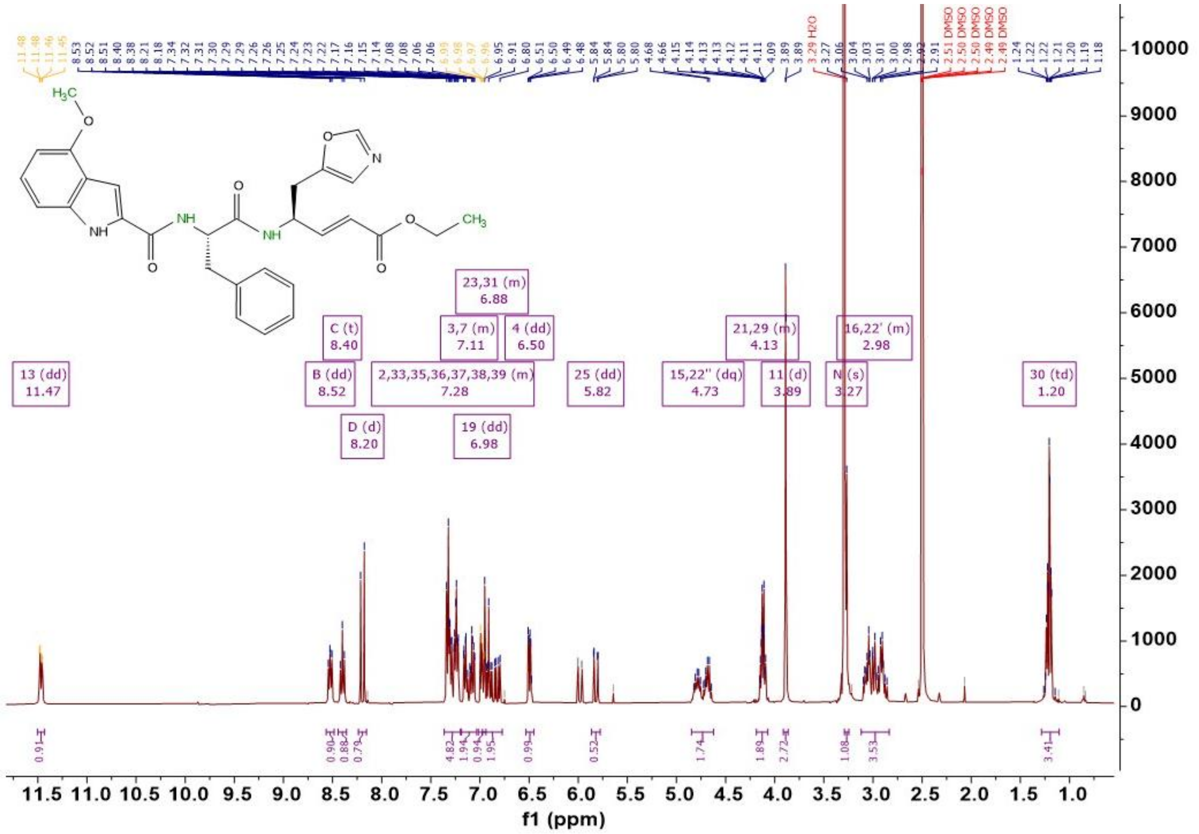
13 (dd) 11.47
C (t) 8.40
B (dd) 8.52
D (d) 8.20
23,31 (m) 6.88
3,7 (m) 7.11
4 (dd) 6.50
2,33,35,36,37,38,39 (m) 7.28
19 (dd) 6.98
25 (dd) 5.82
15,22'' (dq) 4.73
21,29 (m) 4.13
11 (d) 3.89
16,22' (m) 2.98
N (s) 3.27
30 (td) 1.20
f1 (ppm)

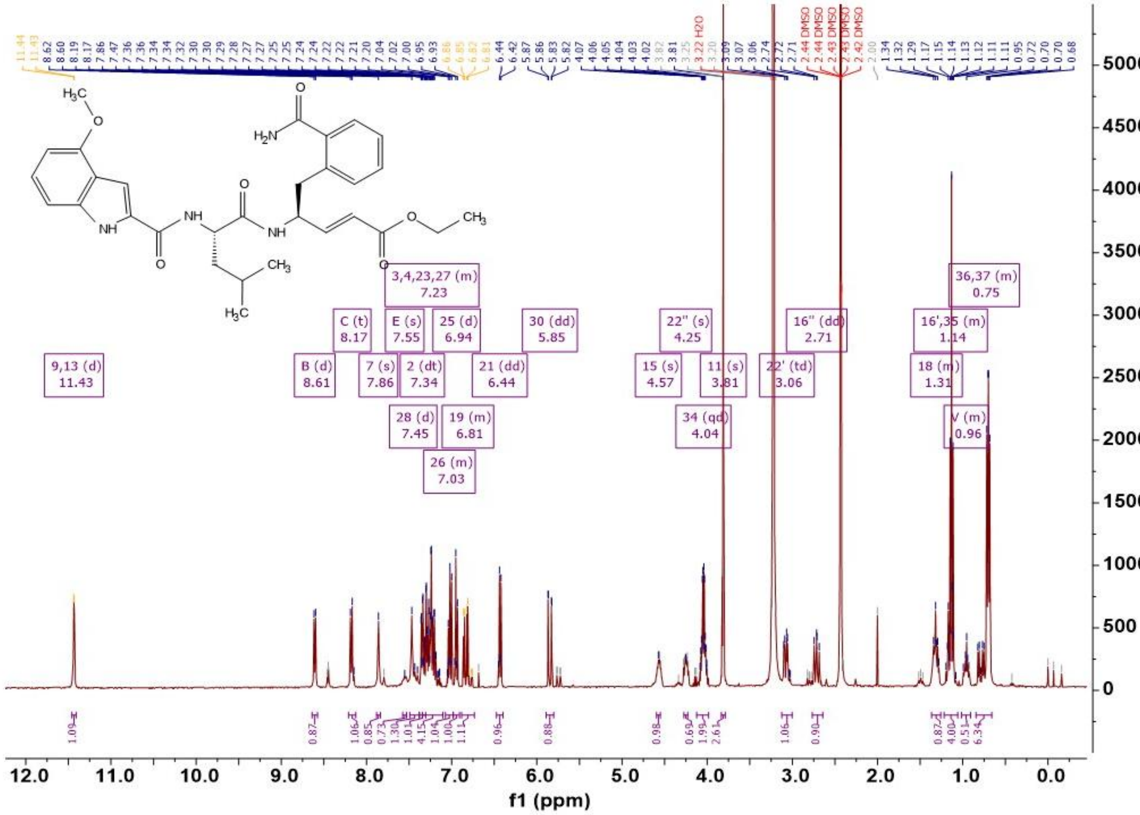

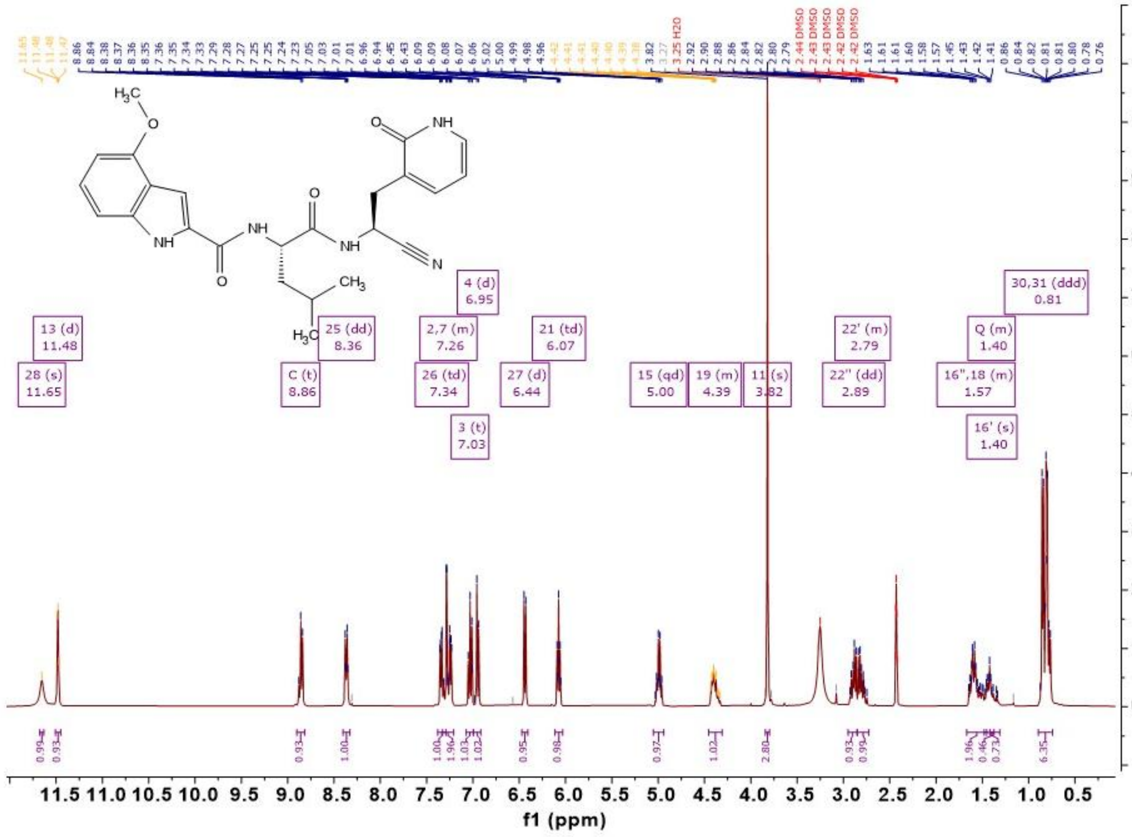

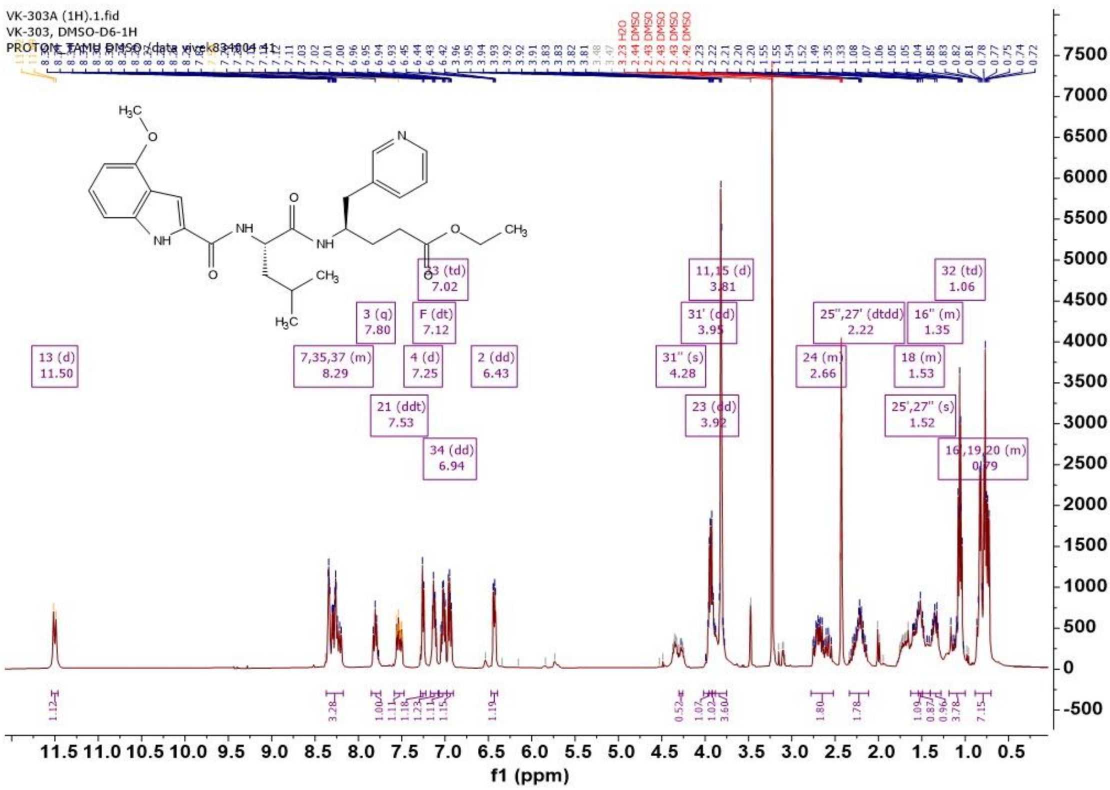

VK-303A (1H).1.fid
VK-303, DMSO-D6-1H
f1 (ppm)

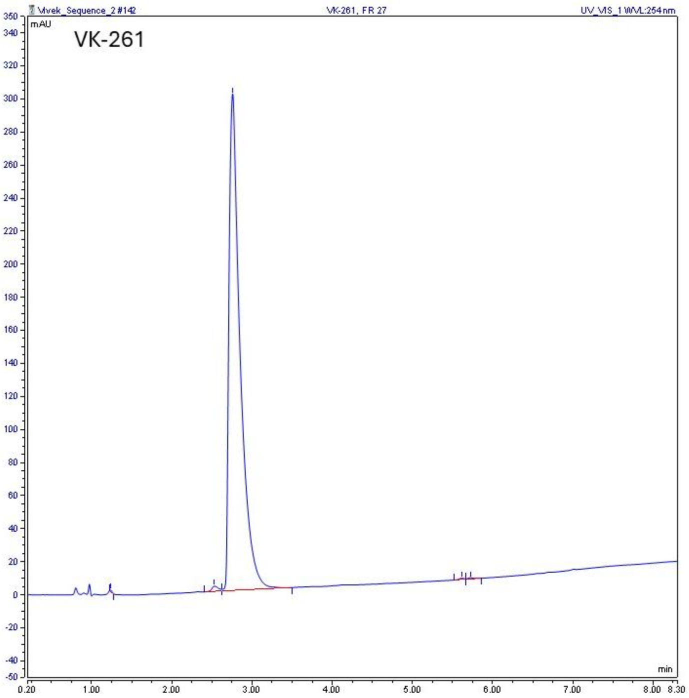

VK-261
mAU
min

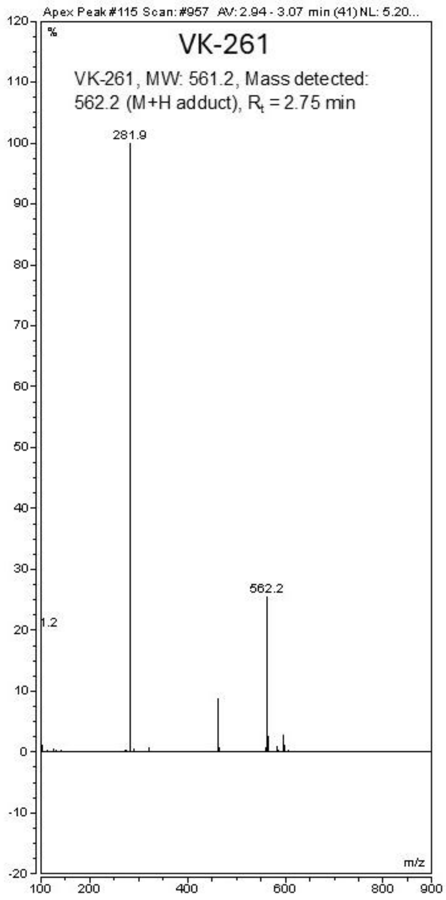
Apex Peak #115 Scan: #957 AV: 2.94 - 3.07 min (41) NL: 5.20...
VK-261
VK-261, MW: 561.2, Mass detected:
562.2 (M+H adduct), R_t = 2.75 min
281.9
562.2
m/z

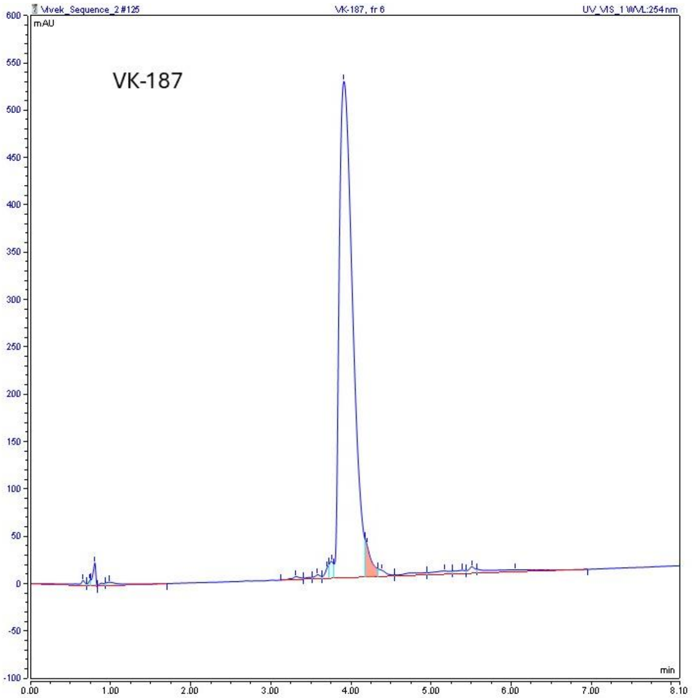
Vivek_Sequence_2 #125
VK-187, fr 6
UV_VIS_1 WVL:254 nm
mAU
VK-187
min

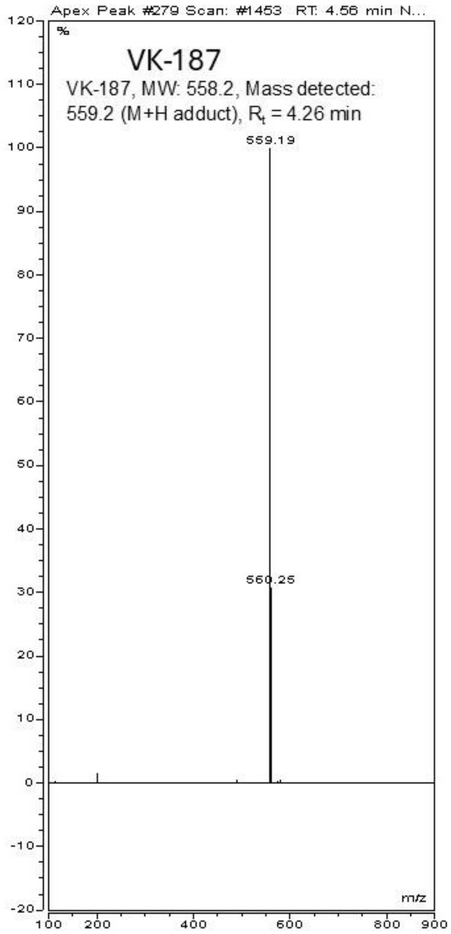
Apex Peak #279 Scan: #1453 RT: 4.56 min N...
%
VK-187
VK-187, MW: 558.2, Mass detected:
559.2 (M+H adduct), R_t = 4.26 min
559.19
560.25
m/z

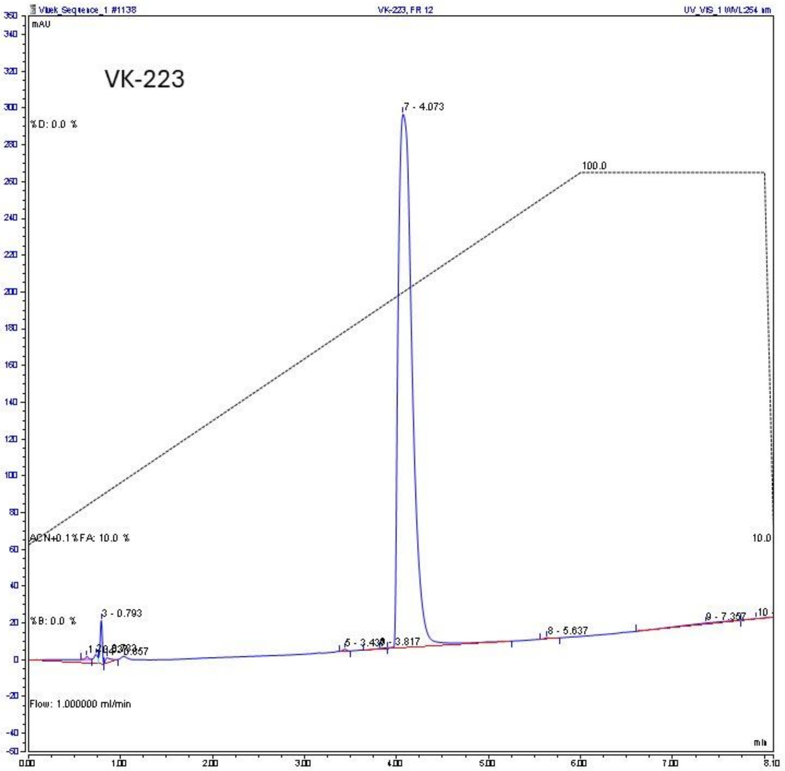
VK-223
7 - 4.073
3 - 0.793
8 - 5.637
9 - 7.357
100.0
10.0
%D: 0.0 %
%B: 0.0 %
ACN+0.1%FA: 10.0 %
Flow: 1.000000 ml/min
mAU
min

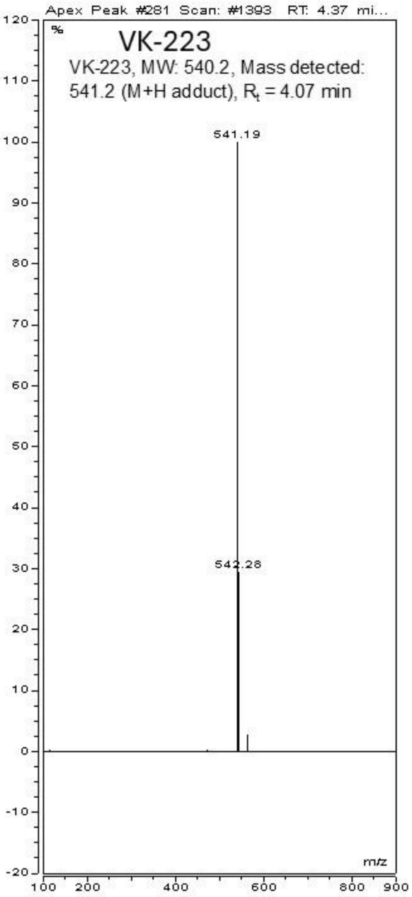
VK-223
VK-223, MW: 540.2, Mass detected: 541.2 (M+H adduct), R_t = 4.07 min
541.19
542.28
m/z

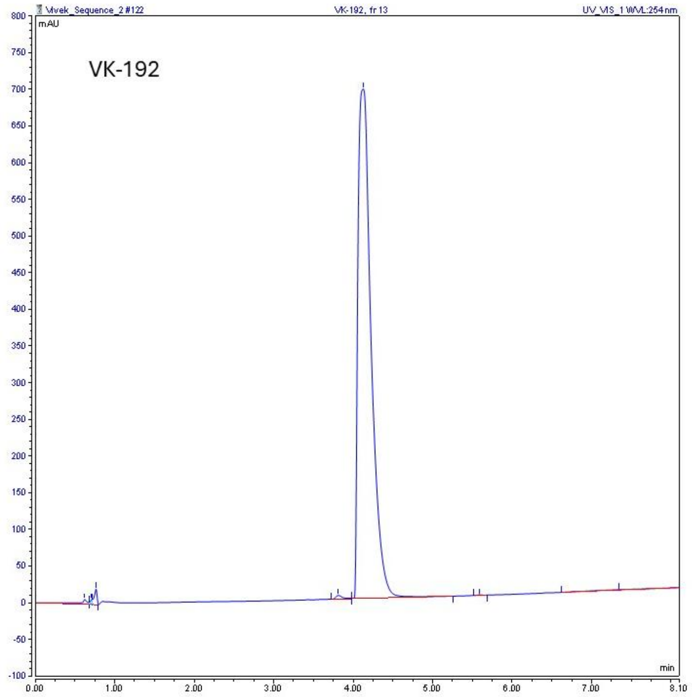
Vivek_Sequence_2 #122
VK-192, fr 13
UV_VIS_1 WVL:254 nm
mAU
VK-192
min

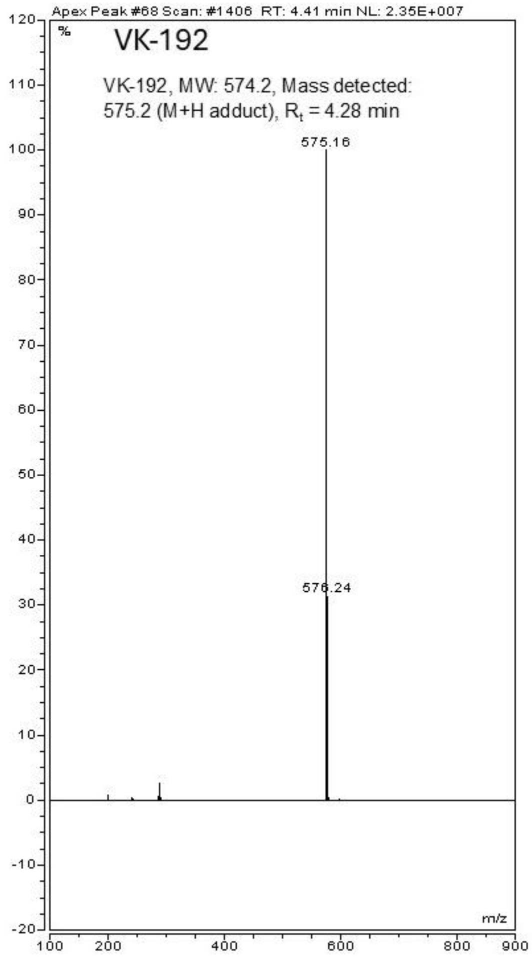
Apex Peak #68 Scan: #1406 RT: 4.41 min NL: 2.35E+007
%
VK-192
VK-192, MW: 574.2, Mass detected:
575.2 (M+H adduct), R_t = 4.28 min
575.16
576.24
m/z

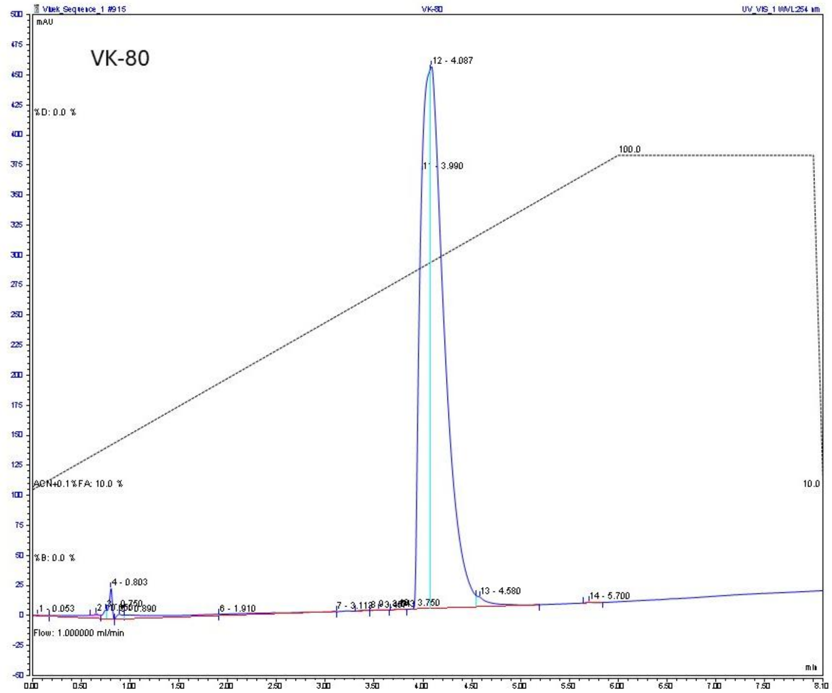
VK-80
12 - 4.087
11 - 3.990
13 - 4.580
14 - 5.700
4 - 0.803
6 - 1.910
1 - 0.053
100.0
10.0
%D: 0.0 %
ACN+0.1%FA: 10.0 %
%B: 0.0 %
Flow: 1.000000 ml/min
mAU
min

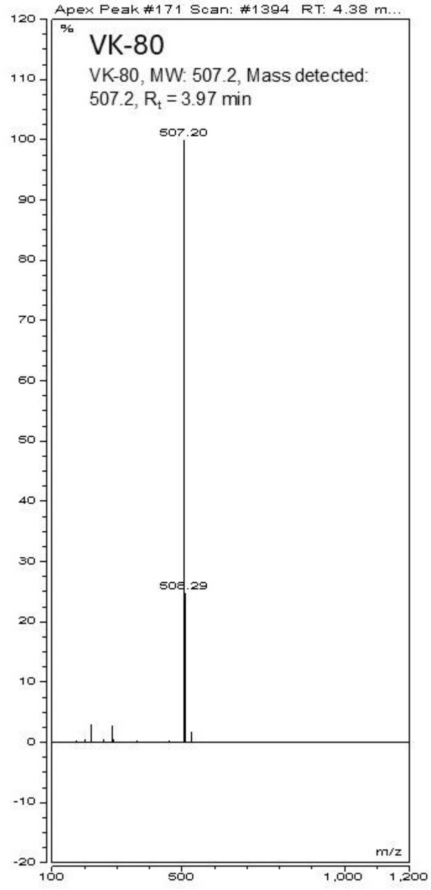
Apex Peak #171 Scan: #1394 RT: 4.38 m...
VK-80
VK-80, MW: 507.2, Mass detected: 507.2, R$_t$ = 3.97 min
507.20
508.29
m/z

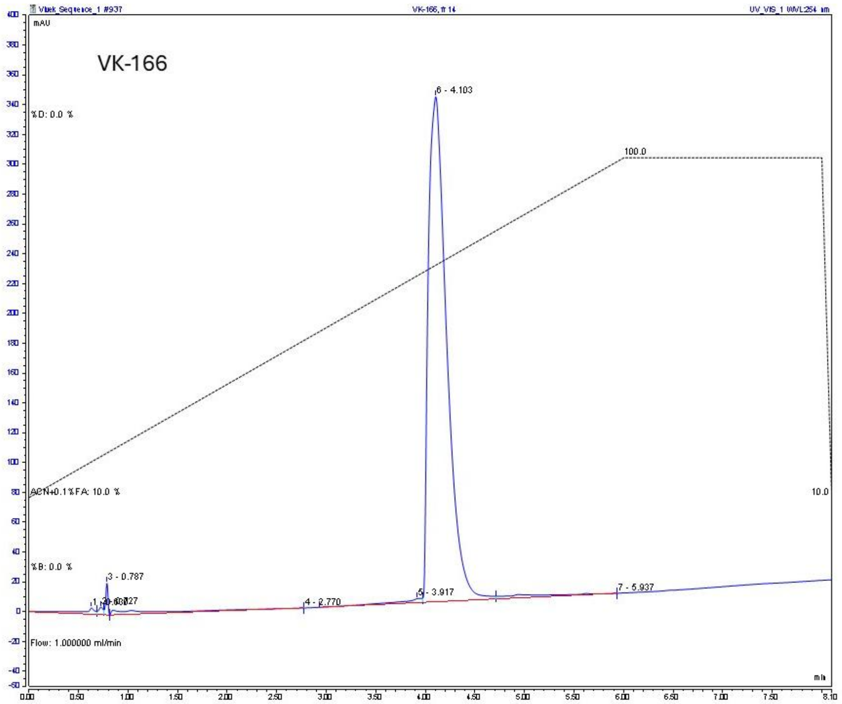
VK-166
6 - 4.103
3 - 0.787
4 - 2.770
5 - 3.917
7 - 5.937
100.0
10.0
%D: 0.0 %
ACN+0.1%FA: 10.0 %
%B: 0.0 %
Flow: 1.000000 ml/min
mAU
min

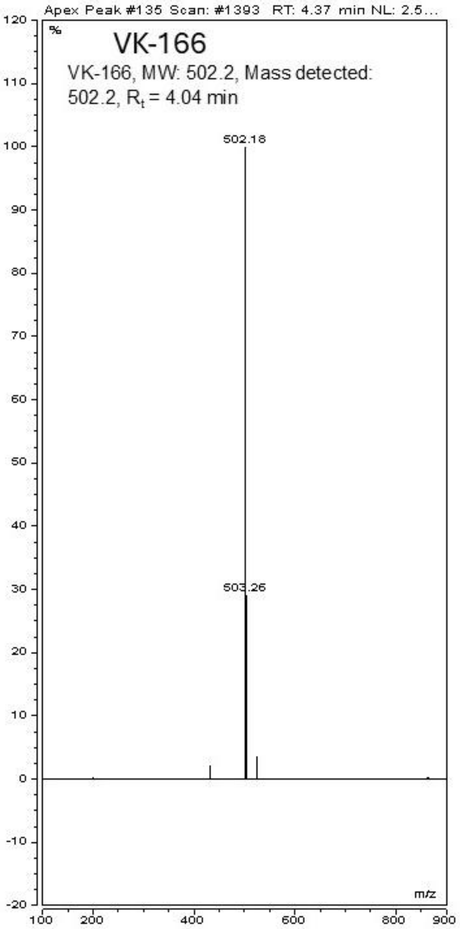
Apex Peak #135 Scan: #1393 RT: 4.37 min NL: 2.5...
VK-166
VK-166, MW: 502.2, Mass detected: 502.2, R_t = 4.04 min
502.18
503.26
m/z

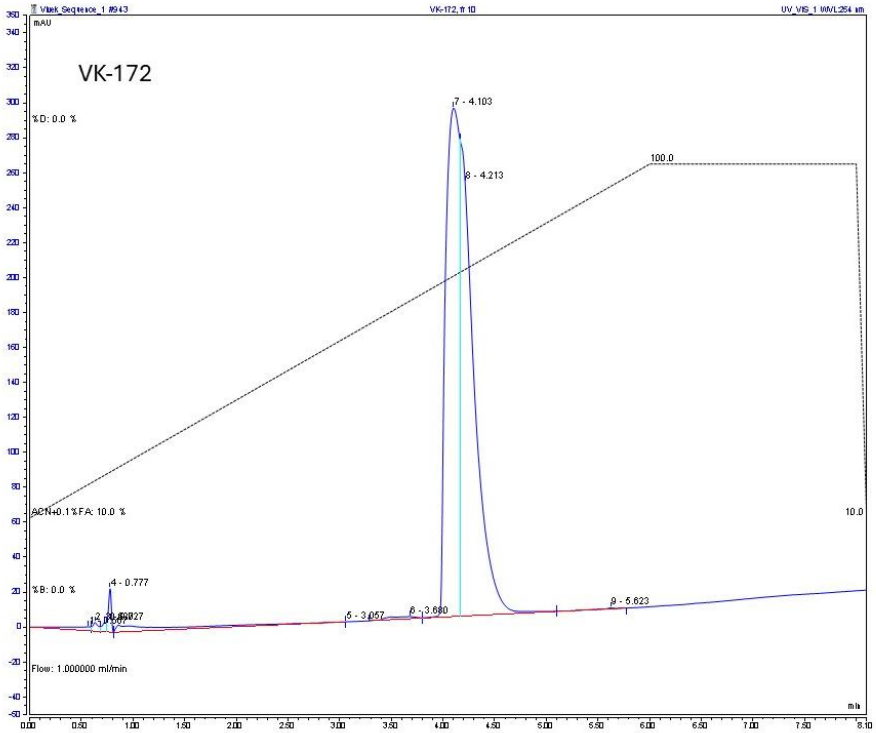
Vitek_Sequence_1 #943
VK-172, ft 10
UV_VIS_1 WVL:254 nm
mAU
VK-172
%D: 0.0 %
7 - 4.103
8 - 4.213
100.0
ACN+0.1%FA: 10.0 %
10.0
4 - 0.777
%B: 0.0 %
5 - 3.057
6 - 3.680
9 - 5.623
Flow: 1.000000 ml/min
min

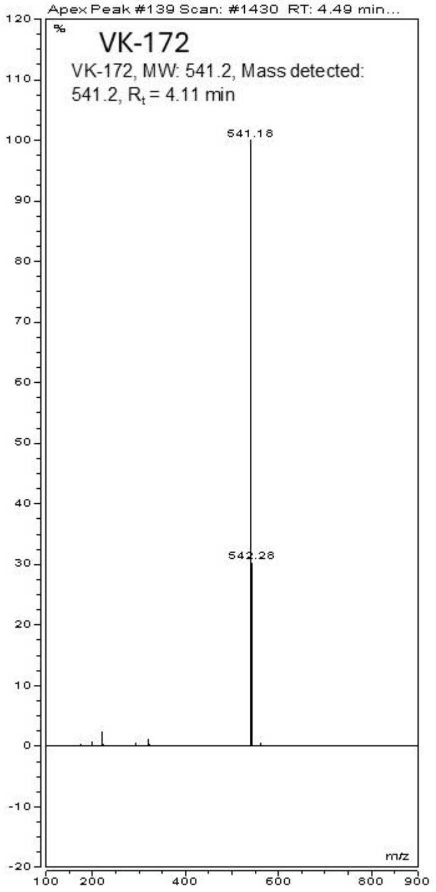
Apex Peak #139 Scan: #1430 RT: 4.49 min...
%
VK-172
VK-172, MW: 541.2, Mass detected: 541.2, R$_t$ = 4.11 min
541.18
542.28
m/z

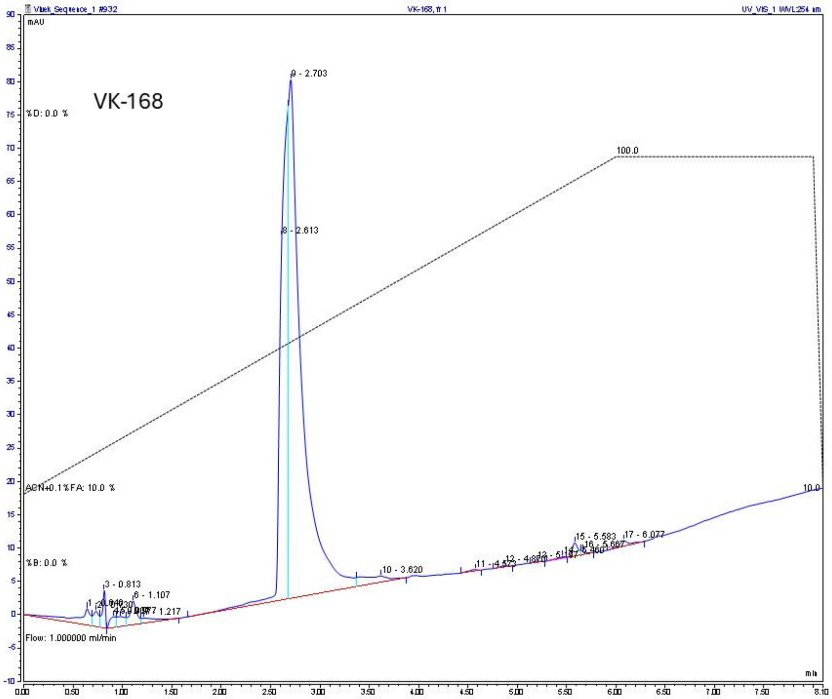
VK-168
9 - 2.703
8 - 2.613
100.0
10.0
10 - 3.620
3 - 0.813
6 - 1.107
%D: 0.0 %
ACN+0.1%FA: 10.0 %
%B: 0.0 %
Flow: 1.000000 ml/min
mAU
min

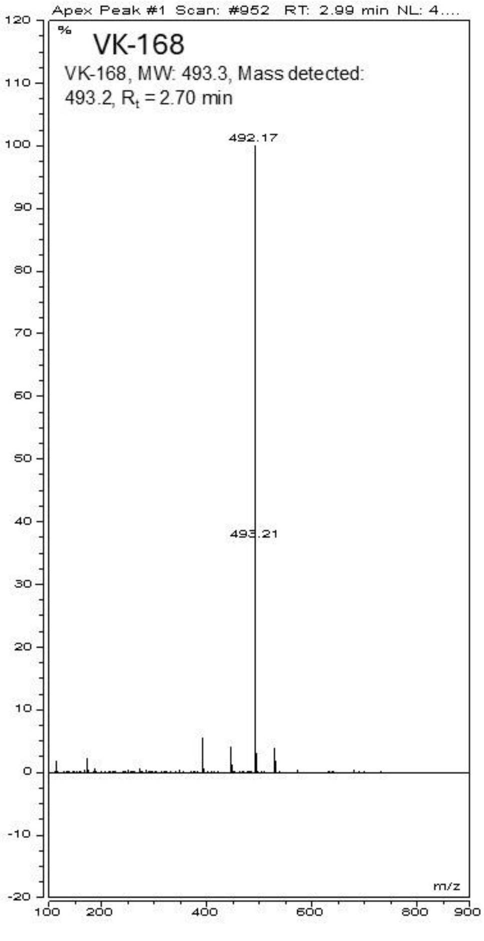
Apex Peak #1 Scan: #952 RT: 2.99 min NL: 4....
VK-168
VK-168, MW: 493.3, Mass detected: 493.2, R_t = 2.70 min
492.17
493.21
m/z

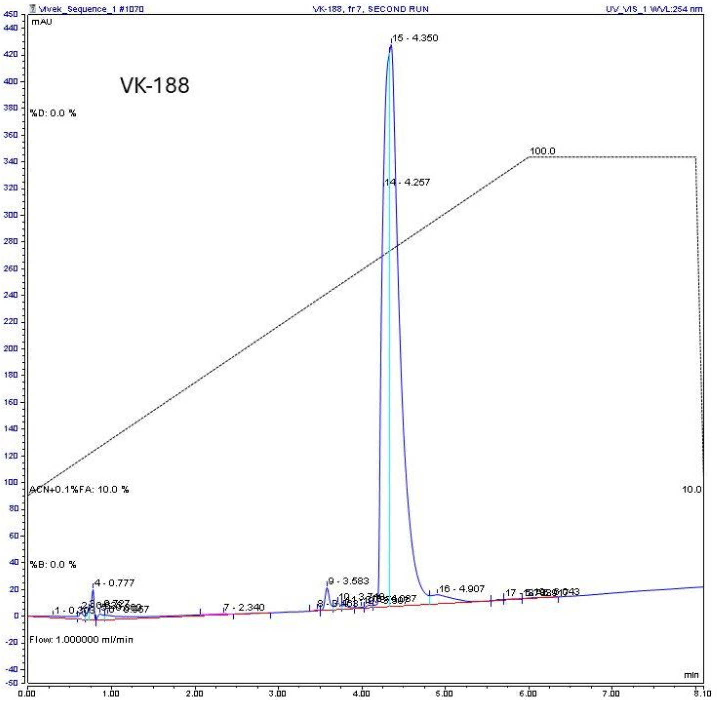
Vivek_Sequence_1 #1070
VK-188, fr 7, SECOND RUN
UV_VIS_1 WVL:254 nm
mAU
VK-188
%D: 0.0 %
100.0
15 - 4.350
14 - 4.257
ACN+0.1%FA: 10.0 %
10.0
%B: 0.0 %
4 - 0.777
9 - 3.583
16 - 4.907
7 - 2.340
Flow: 1.000000 ml/min
min

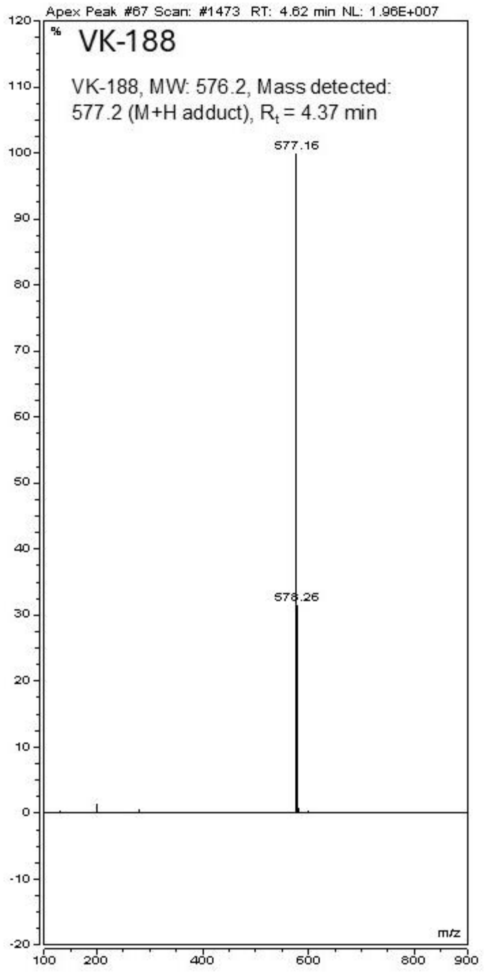
Apex Peak #67 Scan: #1473 RT: 4.62 min NL: 1.96E+007
%
VK-188
VK-188, MW: 576.2, Mass detected:
577.2 (M+H adduct), Rt = 4.37 min
577.16
578.26
m/z

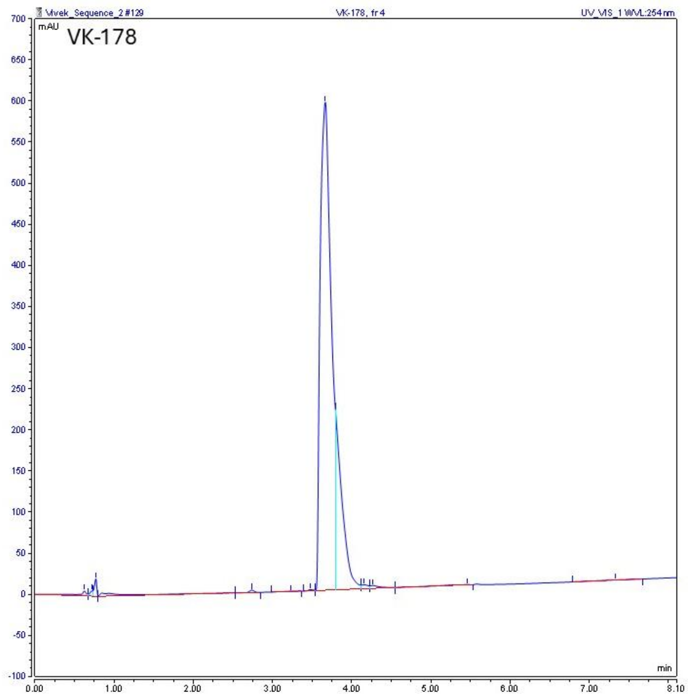
Vivek_Sequence_2 #129
VK-178, fr 4
UV_VIS_1 WVL:254 nm
mAU
VK-178
min

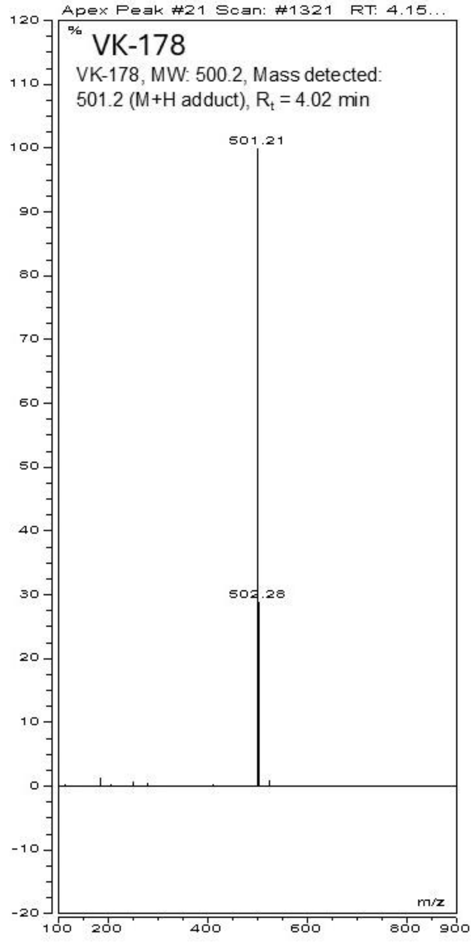
Apex Peak #21 Scan: #1321 RT: 4.15...
%
VK-178
VK-178, MW: 500.2, Mass detected: 501.2 (M+H adduct), R$_t$ = 4.02 min
501.21
502.28
m/z

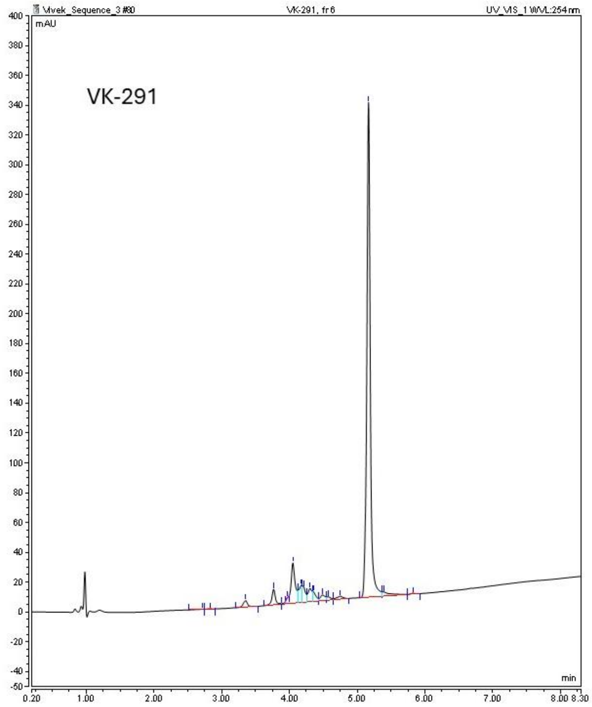
Vivek_Sequence_3 #80
VK-291, fr 6
UV_VIS_1 WVL:254 nm
mAU
VK-291
min

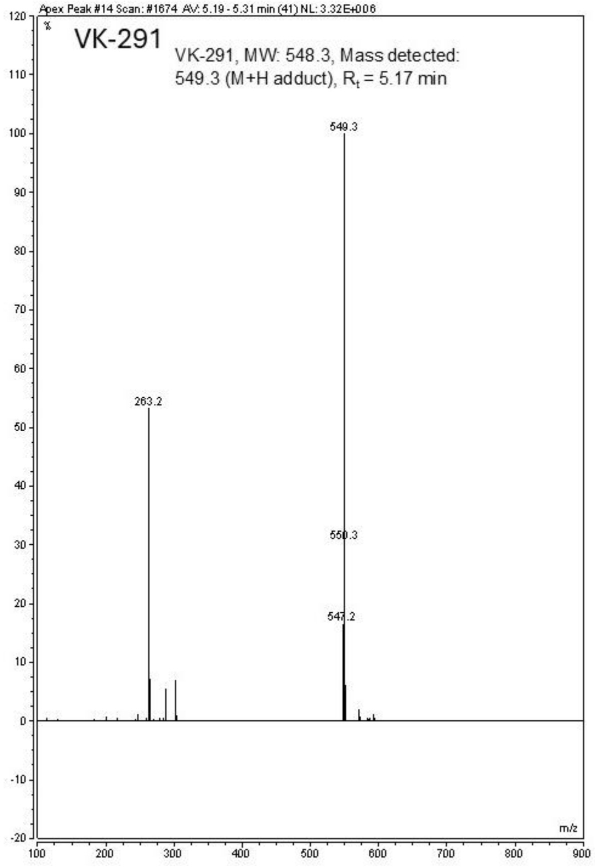
Apex Peak #14 Scan: #1674 AV: 5.19 - 5.31 min (41) NL: 3.32E+006
%
VK-291
VK-291, MW: 548.3, Mass detected: 549.3 (M+H adduct), R_t = 5.17 min
549.3
263.2
550.3
547.2
m/z

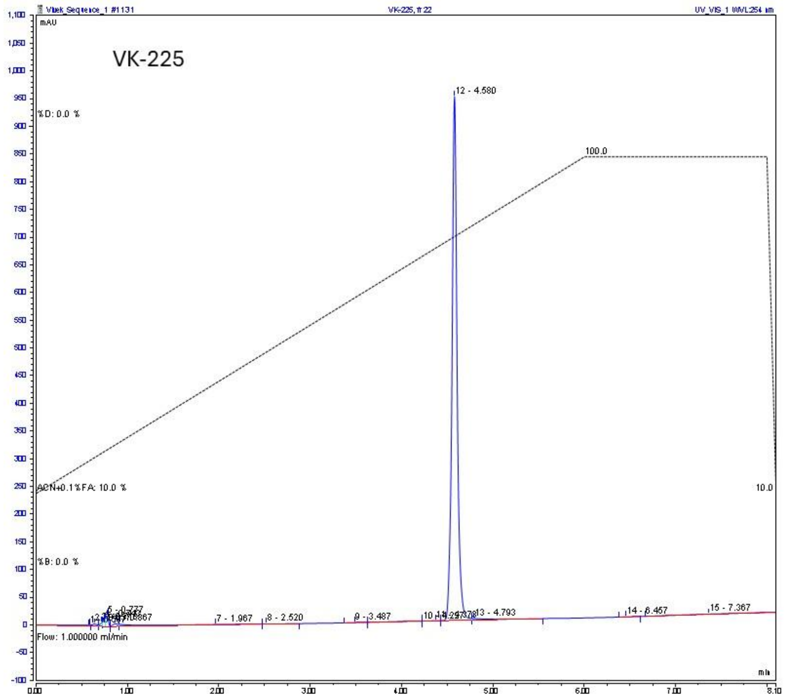
VK-225
12 - 4.580
%D: 0.0 %
100.0
ACN+0.1%FA: 10.0 %
10.0
%B: 0.0 %
Flow: 1.000000 ml/min
7 - 1.967
8 - 2.520
9 - 3.487
13 - 4.793
14 - 6.457
15 - 7.367

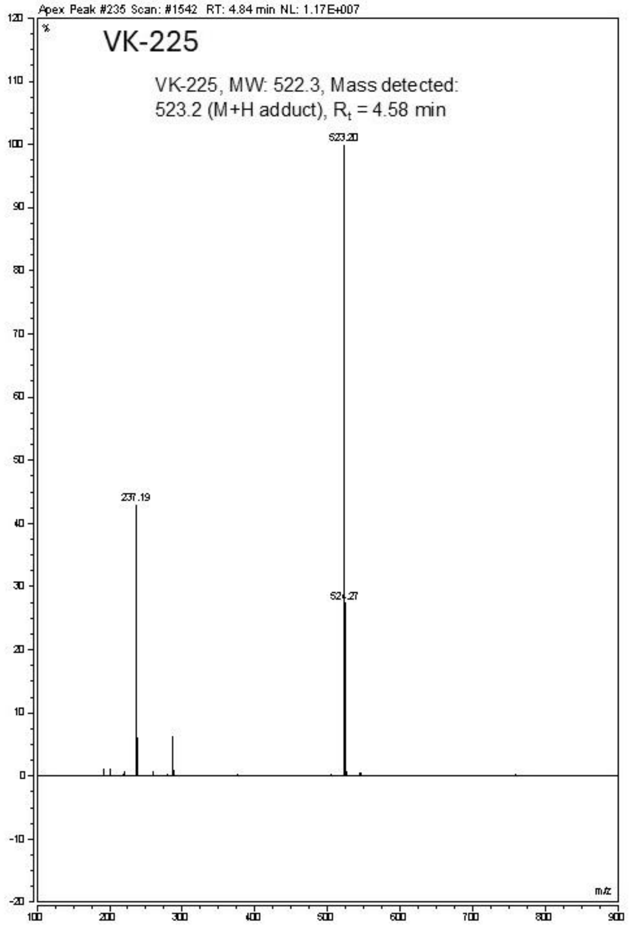
Apex Peak #235 Scan: #1542 RT: 4.84 min NL: 1.17E+007
VK-225
VK-225, MW: 522.3, Mass detected:
523.2 (M+H adduct), R_t = 4.58 min
523.20
237.19
524.27
m/z

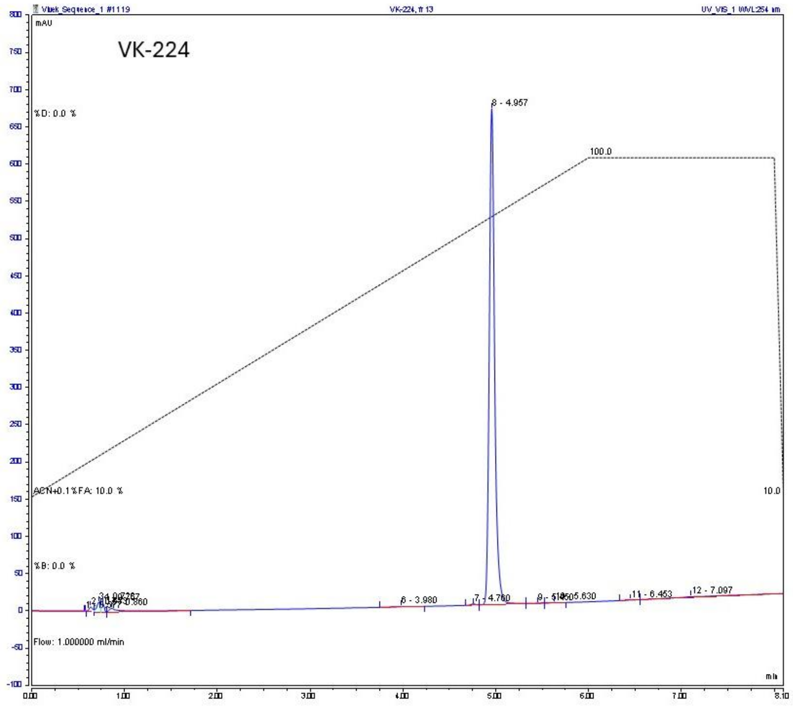
VK-224
Vlasek_Sequence_1 #1119
VK-224, tf 13
UV_VIS_1 WVL:254 nm
mAU
min
%D: 0.0 %
ACN+0.1%FA: 10.0 %
%B: 0.0 %
Flow: 1.000000 ml/min
8 - 4.957
100.0
10.0
6 - 3.980
12 - 7.097

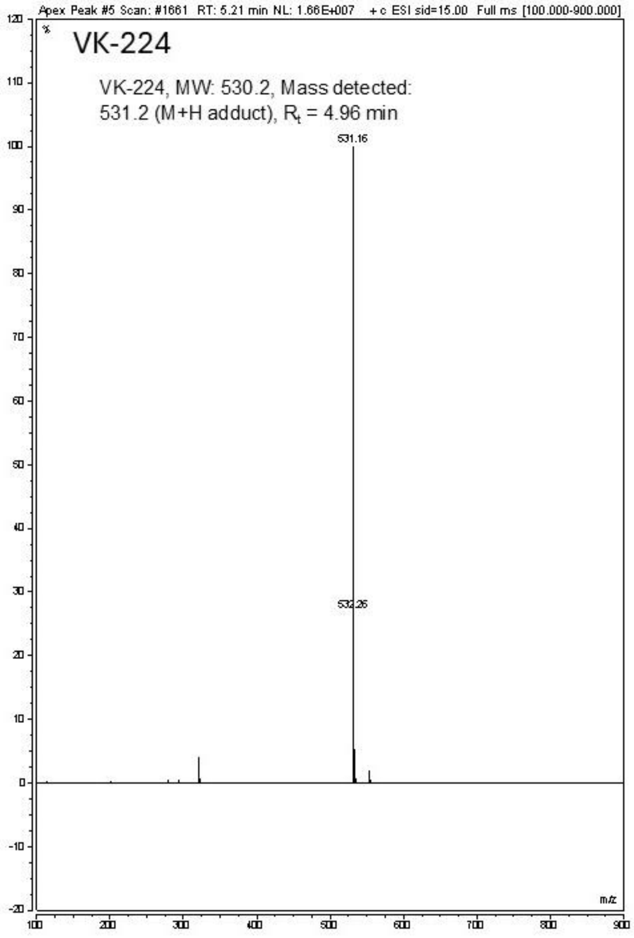
Apex Peak #5 Scan: #1661 RT: 5.21 min NL: 1.66E+007 + c ESI sid=15.00 Full ms [100.000-900.000]
VK-224
VK-224, MW: 530.2, Mass detected:
531.2 (M+H adduct), R_t = 4.96 min
531.16
532.26
m/z

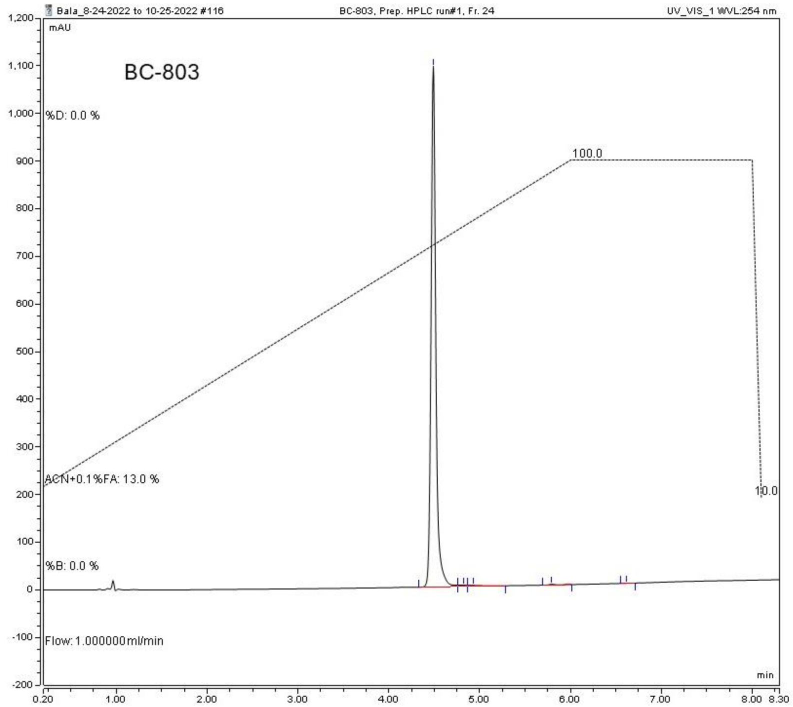
Bala_8-24-2022 to 10-25-2022 #116
BC-803, Prep. HPLC run#1, Fr. 24
UV_VIS_1 WVL:254 nm
mAU
BC-803
%D: 0.0 %
100.0
ACN+0.1%FA: 13.0 %
10.0
%B: 0.0 %
Flow: 1.000000 ml/min
min

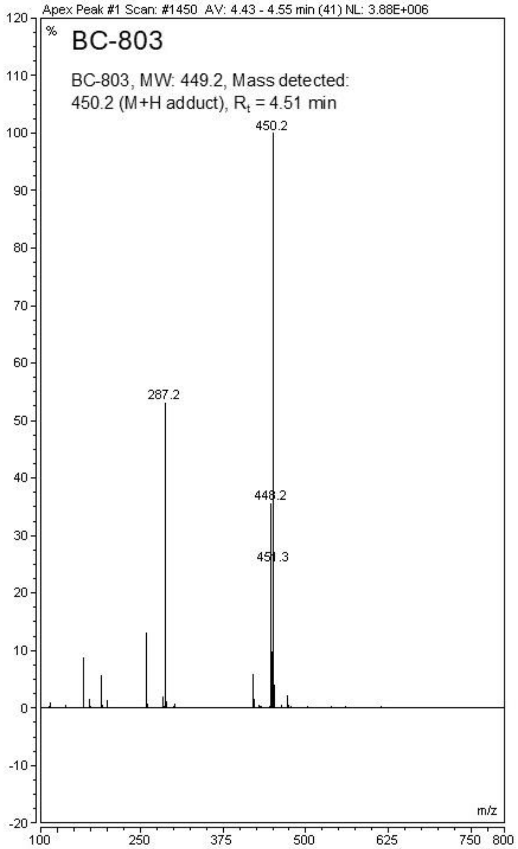
Apex Peak #1 Scan: #1450 AV: 4.43 - 4.55 min (41) NL: 3.88E+006
%
BC-803
BC-803, MW: 449.2, Mass detected:
450.2 (M+H adduct), R_t = 4.51 min
450.2
287.2
448.2
451.3
m/z

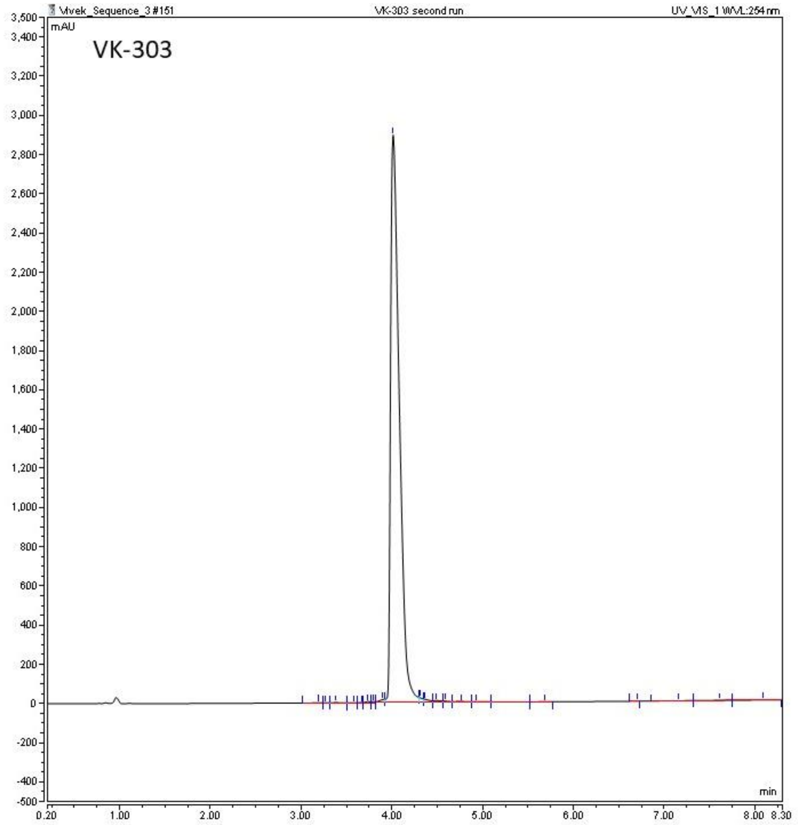
Vivek_Sequence_3 #151
VK-303 second run
UV_VIS_1 WVL:254 nm
mAU
VK-303
min

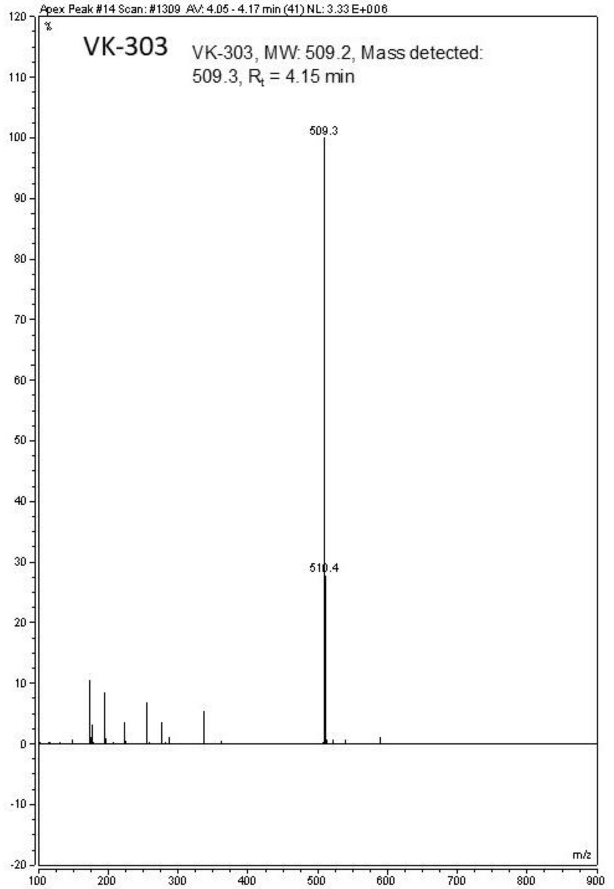
Apex Peak #14 Scan: #1309 AV: 4.05 - 4.17 min (41) NL: 3.33 E+006
%
VK-303
VK-303, MW: 509.2, Mass detected: 509.3, R_t = 4.15 min
509.3
510.4
m/z

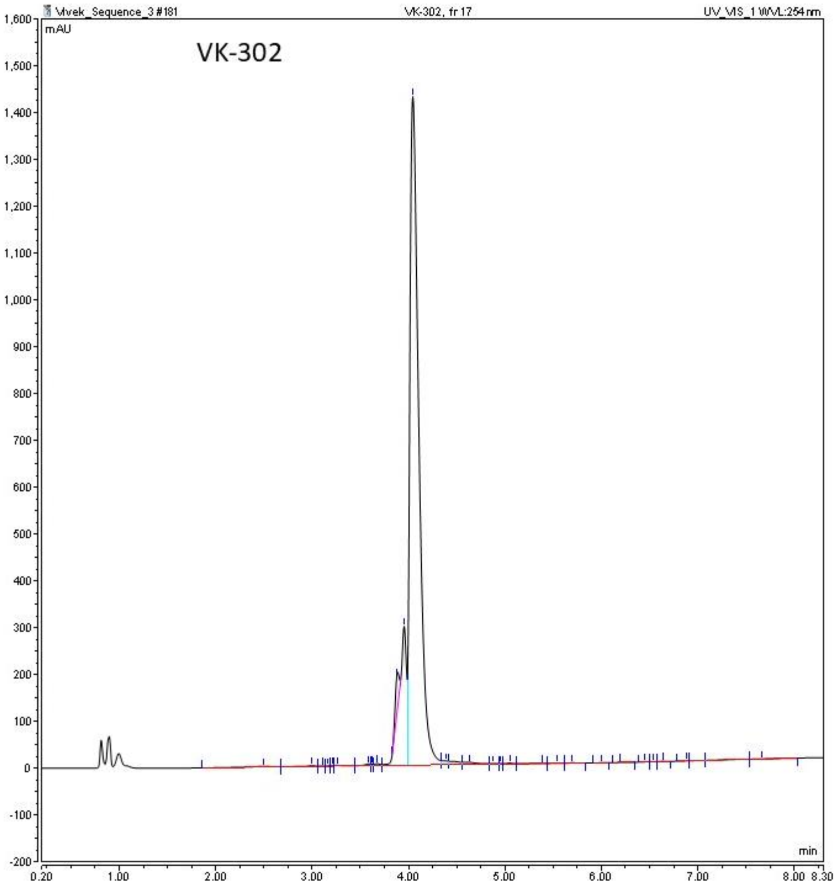

Vivek_Sequence_3 #181
VK-302, fr 17
UV_VIS_1 WVL:254 nm
mAU
VK-302
min

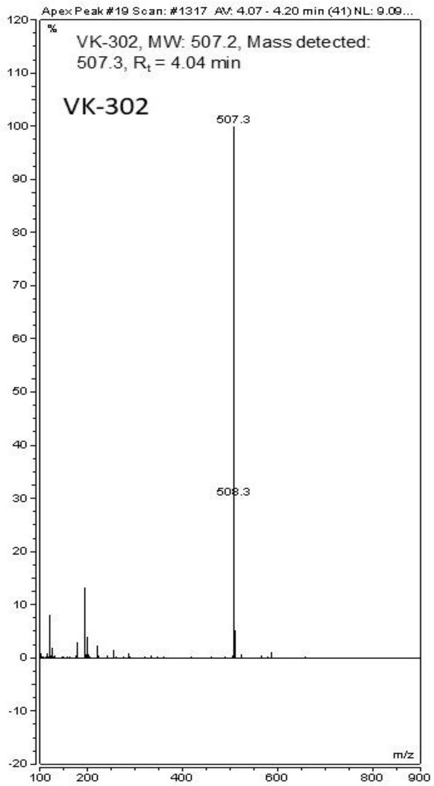

Figure S15: NMR spectra, mass spectra, and HPLC chromatograms of compounds prepared as described in the text

Table S3. UHMR instrument parameters.

Parameters	*Values*
m/z range	**350-6000**
Resolution	**6250**
In-source CID (V)	**30**
In-Source CE (eV)	**30-150**
Source temperature (°C)	**250**
Capillary voltage (kV)	**16**
Source DC offset (V)	**21**
Inject flatapole DC (V)	**5**
Inter flatapole lens (V)	**5**
Bent flatapole DC (V)	**4**
Transfer multipole DC (V)	**0**
C-Trap Entrance Lens Tune Offset (V)	**1.8**
Pressure (mbar)	**5**

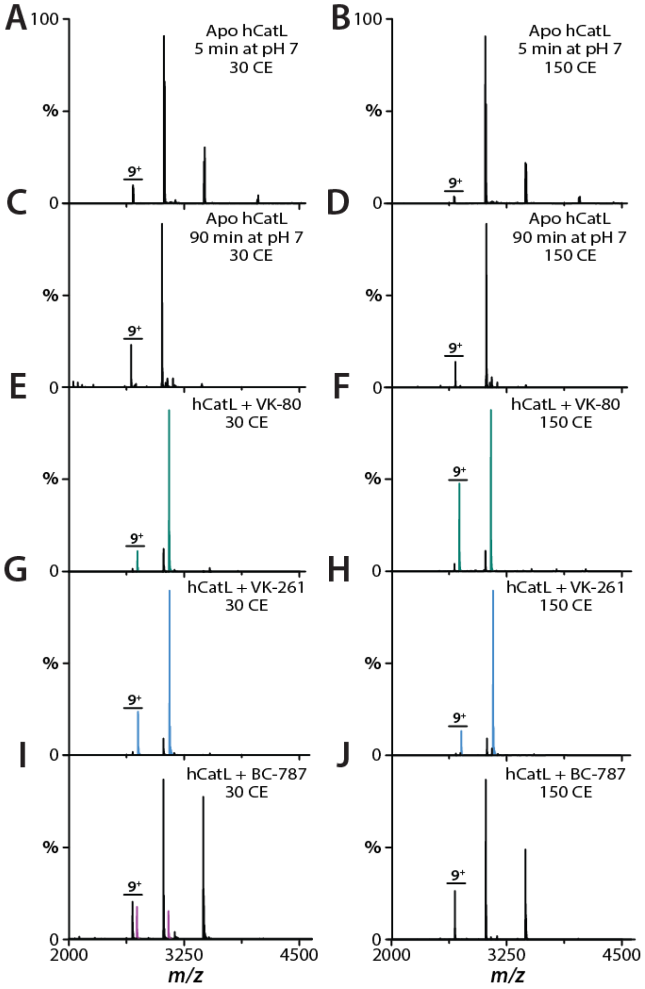

Figure S16. Native MS analysis of hCatL with VK-80, VK-261, and BC-787. (A) Native mass spectrum of apo hCatL 5 minutes after exchanged into 200 mM ammonium acetate (pH 7.0) at 30 CE and (B) at 150 CE. (C) Native mass spectrum of apo hCatL 90 minutes after exchanged into 200 mM ammonium acetate (pH 7.0) at 30 CE and (D) at 150 CE. (E) Native mass spectrum of hCatL with a 5-fold molar excess of VK-80 inhibitor showed a shift in peaks (green) at 30 CE and (F) at 150 CE. (G) Native mass spectrum of hCatL with a 5-fold molar excess of VK-261 inhibitor showed a shift in peaks (blue) at 30 CE and (H) at 150 CE. (I) Native mass spectrum of hCatL with a 5-fold molar excess of VK-787 inhibitor showed a shift in peaks (pink) at 30 CE and (J) a lack of shift of peaks with increased energy at 150 CE.
